# Using a Network Model to Assess Risk of Forest Pest Spread via Recreational Travel

**DOI:** 10.1371/journal.pone.0102105

**Published:** 2014-07-09

**Authors:** Frank H. Koch, Denys Yemshanov, Robert A. Haack, Roger D. Magarey

**Affiliations:** 1 United States Department of Agriculture, Forest Service, Southern Research Station, Eastern Forest Environmental Threat Assessment Center, Research Triangle Park, North Carolina, United States of America; 2 Natural Resources Canada, Canadian Forest Service, Great Lakes Forestry Centre, Sault Ste. Marie, Ontario, Canada; 3 United States Department of Agriculture, Forest Service, Northern Research Station, East Lansing, Michigan, United States of America; 4 Center for Integrated Pest Management, North Carolina State University, Raleigh, North Carolina, United States of America; CSIRO, Australia

## Abstract

Long-distance dispersal pathways, which frequently relate to human activities, facilitate the spread of alien species. One pathway of concern in North America is the possible spread of forest pests in firewood carried by visitors to campgrounds or recreational facilities. We present a network model depicting the movement of campers and, by extension, potentially infested firewood. We constructed the model from US National Recreation Reservation Service data documenting more than seven million visitor reservations (including visitors from Canada) at campgrounds nationwide. This bi-directional model can be used to identify likely origin and destination locations for a camper-transported pest. To support broad-scale decision making, we used the model to generate summary maps for 48 US states and seven Canadian provinces that depict the most likely origins of campers traveling from outside the target state or province. The maps generally showed one of two basic spatial patterns of out-of-state (or out-of-province) origin risk. In the eastern United States, the riskiest out-of-state origin locations were usually found in a localized region restricted to portions of adjacent states. In the western United States, the riskiest out-of-state origin locations were typically associated with major urban areas located far from the state of interest. A few states and the Canadian provinces showed characteristics of both patterns. These model outputs can guide deployment of resources for surveillance, firewood inspections, or other activities. Significantly, the contrasting map patterns indicate that no single response strategy is appropriate for all states and provinces. If most out-of-state campers are traveling from distant areas, it may be effective to deploy resources at key points along major roads (e.g., interstate highways), since these locations could effectively represent bottlenecks of camper movement. If most campers are from nearby areas, they may have many feasible travel routes, so a more widely distributed deployment may be necessary.

## Introduction

Long-distance dispersal is a critical factor in the spread of invasive alien species. Many long-distance dispersal pathways are associated with human activities [1], [2]. For instance, the importance of international trade in facilitating invasions is well recognized [3]–[5]. However, the growing invasive alien species problem is more broadly associated with increased human mobility in a variety of contexts, including recreational travel [1], [2], [6]–[8]. Regrettably, pathways of human-mediated dispersal not directly related to trade, such as recreational travel, have often been overlooked.

Travel for the purpose of outdoor recreation is popular in North America, and total participation in camping and other nature-based recreational activities is expected to grow in the future [9]–[11]. For campers in the US and Canada, national parks, state/provincial parks, and other public lands are among the most preferred destinations [12], [13]. Many of these destinations are within or near large areas of forest or woodland. Hence, it is logical that camper travel – and in particular, the movement of untreated firewood by campers – has received much attention as a potential invasion pathway for forest pests [14]–[19].

Untreated firewood is known to harbor a variety of organisms, particularly bark- and wood-infesting insects [20]. Perhaps most notably, firewood movement has been linked to the spread of two destructive alien insects, the Asian longhorned beetle (*Anoplophora glabripennis* (Motschulsky)) and the emerald ash borer (*Agrilus planipennis* Fairmaire), in both urban and natural forests in the eastern US and Canada [7], [21], [22]. Two points emphasize the magnitude of the threat that firewood poses to forests. Firstly, firewood is regularly moved for recreational purposes. Surveys performed in several US national and state parks indicate that a large percentage of campers (≈66% in some cases) bring firewood from home or another distant location [15], [19]. In fact, it is not unusual for campers to carry wood cut from dead or dying trees on their property [15]. Secondly, the rate of firewood infestation by forest pests, especially insects, may be high. Haack et al. [23] examined more than 1 000 pieces of firewood surrendered at the Mackinac Bridge in the US state of Michigan in April, July, and September 2008. The bridge carries vehicular traffic between Michigan’s Lower and Upper Peninsulas; at the time of the surveys, transport of firewood across the bridge was prohibited by a state-level *A. planipennis* quarantine. The researchers found live bark- and wood-boring insects ( = borers) in 23% of the firewood pieces, and an additional 41% displayed evidence of prior borer infestation. Jacobi et al. [20] purchased more than 400 bundles of firewood from retailers in the US states of Colorado, New Mexico, Utah, and Wyoming during a three-year period (2007–2009). Caging the firewood to measure insect emergence, they reported that live insects emerged from 47% of the bundles over 18 months of rearing time, with an average of 11 insects emerging from each infested bundle.

To protect their forests from firewood-borne pests, a majority of US states and Canadian provinces have implemented restrictions on firewood movement, in some cases prohibiting out-of-state/province firewood unless treated, or limiting transport to within a small distance radius [18], [19], [23], [24]. However, governments face a significant challenge in setting distance restrictions for firewood movement. Tobin et al. [18] addressed this issue, employing a simulation modeling approach to determine which campgrounds in a region of interest were most likely to receive infested firewood based on the hypothetical distribution of a forest pest and a given allowable distance for moving firewood. This analysis [18] focused on the proximity of target campgrounds to locations (e.g., urban areas) where a forest pest would likely originate. Notably, the authors did not attempt to simulate the movement of firewood by a specific subset of campers due to a lack of data describing this behavior at fine spatial scales. Instead, they simulated the travel of all campers, under the assumption that some unspecified proportion would be carrying pest-infested firewood.

Koch et al. [16] similarly adopted an indirect approach of examining general camper travel patterns rather than transport and deliberate use of firewood by campers. The study employed data from the US National Recreation Reservation Service (NRRS), an online reservation system for campgrounds and related facilities operated by the US Forest Service and other federal agencies. The analysis involved calculating, for more than seven million individual reservation records, the travel distance between a visitor’s origin location and destination campground. These distance data were then fitted with various theoretical probability distributions to identify functional forms that could be applied (i.e., as dispersal kernels) in mathematical modeling of spread [25]–[28]. Although roughly half of the trips recorded in the NRRS data involved one-way travel of 100 km or less, approximately 10% of the trips were greater than 500 km, and some were more than 5 000 km. Thus, while only a small percentage of campground visits may involve the movement of infested firewood, the risk of pest spread, including long-distance spread, is substantial given the millions of camper visits each year. Koch et al. [16] also found the data to be fairly well fit by theoretical distributions such as the lognormal, suggesting that it is possible to derive a reasonable dispersal kernel to simulate the spread of forest pests via camper travel.

A two-dimensional, kernel-based spread model commonly assumes that the kernel is rotationally symmetric (i.e., omnidirectional), such that the organism of interest is dispersed in the same fashion in all directions from the location of origin [29]. Even if the dispersal kernel is anisotropic (i.e., depicts a greater probability of dispersal in certain directions [30]), the destination locations for dispersing individuals are not defined exactly, since individuals may be dispersed probabilistically to any location that falls within the range of distances specified in the kernel. But for camper-transported firewood or any similar human-mediated pathway, dispersal actually occurs via a system of specific routes (i.e., vectors) and involves a relatively narrow set of possible destinations. Therefore, it seems sensible to apply a network-based, rather than a kernel-based, method for modeling the human-mediated spread of invasive species [31]–[33].

Network transport models describe movement via a lattice of vectors, or links, between a set of interconnected nodes. For networks, the amount of movement along a link between two nodes may be more important than the link’s length when determining the likelihood of spread [34], [35]. With respect to the NRRS data, visitor origin and destination campground locations may be envisioned as two sets of linked nodes, where the strength of the links is defined by the number of campers traveling along them. Here, we adopt this conceptual framework and construct a network model from the NRRS data for analyzing potential invasion patterns associated with camper travel.

A network transport model facilitates analyses that would be far more difficult with a kernel-based spread model [33], [36], [37]. For instance, “forward” pathway analysis deals with the likeliest destinations for a pest dispersed from a particular origin location within the network. It addresses a question that might likewise be answered with a kernel-based model: Where is a pest of interest that is currently (or anticipated to be) at a given location in the network most likely to appear next? However, a network transport model also permits “reverse” pathway analysis, which focuses on identifying the most likely origins for a network location that has been (or is expected to be) invaded by a pest of interest (but note Guichard et al. [38], who applied a kernel-based model for this type of reverse analysis). In our case, the ideal model would directly estimate the likelihoods of infested firewood being transported along the pathways (links) between locations (nodes) documented in the network. Because of the paucity of data describing patterns of firewood usage and infestation rates, our model more generally estimates rates of camper travel along the network’s pathways. We subsequently assume that some constant proportion of all camper travel involves the movement of infested firewood. Given this assumption, the aim of this study was to rank network locations based on the model-derived camper travel rates as a relative measure of firewood-facilitated pest dispersal risk, thereby allowing decision makers to prioritize locations for surveillance or other biosecurity management activities (e.g., targeted education).

In building our network model, we used the same core model structure as Yemshanov et al. [39], who examined the movement of commercial freight (i.e., commodities that could harbor forest pests) among Canadian municipalities and US-Canada border crossings via the region’s road network. In addition to the difference between the underlying data sets, we expanded upon this previous study by summarizing the model outputs at the state or provincial level, which is the typical geographic scale for pest management decision making in the US and Canada. Notably, the outputs all share a single frame of reference, making the states and provinces directly comparable to each other.

## Materials and Methods

### Data pre-processing

The original NRRS data are held by USDA Animal and Plant Health Inspection Service, Plant Protection and Quarantine Division, Fort Collins, CO. The available data spanned the period January 2004–September 2009 and documented approximately 7.2 million visitor reservations, including reservations from Canada, at federally operated campgrounds and recreational facilities throughout the continental US. The format and content of the NRRS data records, each of which included geographic information for a visitor origin location and a destination campground, are described in more detail in Koch et al. [16].

Before implementing these data in the network model, we pre-processed them in two key ways. Firstly, we filtered the reservation records according to a period of peak emergence for bark- and wood-boring insects (i.e., insects most likely to be transported in firewood). As with other categories of insects, the establishment success of any borer species is contingent on its developmental stage at the time of its introduction [31], [40]. Borers typically require 1–3 years to complete a single generation, with the actual length of time depending on the individual species and the nutritional quality of its host tissue [22], [41]. Regardless, we presume that the risk of successful borer invasion is greatest during the time of year when adult insects typically emerge from their current host (a piece of firewood in this case) and begin searching for new hosts [40]. For most borers, adult emergence usually occurs during late spring and early summer [42]–[44].

When implementing the model, we only retained those NRRS reservation records where both the visitor origin location and destination campground location were in the late spring-early summer period when the visit occurred. We intended for the model to be generic for all borer species rather than any one particular species, so we defined this period using a broad (10-week) time window. However, the calendar dates corresponding to the beginning and end of this climatic season vary geographically throughout the US and Canada in relation to factors such as latitude and elevation. Our procedures for accounting for this variation, as well as some of our related assumptions, are outlined in Appendix S1 in the Supporting Information.

Our second pre-processing step related to our ability to parse the selected NRRS data into a set of unique pathway segments. Each reservation record in the NRRS data represented a single “trip” between a pair of origin and destination locations. The full data set included more than 50 000 visitor origin locations (i.e., postal codes) and more than 2 500 destination campgrounds, yielding more than 973 000 pairwise origin-destination combinations with at least one trip between them. Fundamentally, the network model was formulated as a first-order transition matrix [45] that operates on every pairwise combination of origin and destination locations (i.e., nodes) in the network. It was computationally impractical to build a transition matrix model involving this many combinations, so – in contrast with previous applications of this modeling framework [37], [39] to smaller initial data sets – we aggregated the NRRS data at a moderately coarse spatial resolution. We divided our area of interest (the contiguous US and southern Canada) into a continuous grid of 15×15 km map cells, and then assigned all of the origin and destination locations documented in the NRRS data to the map cell in which they fell. For each cell, we calculated the total number of trips associated with the origin locations it contained, and separately did the same for its destination locations. Additionally, we dropped any pairwise combination that was associated with three or fewer trips during our study period (January 2004–September 2009). This was necessary for the aggregation process to conform to our available computational resources. Although omitting these pairwise combinations limited our ability to model certain low-frequency events (e.g., campers traveling from small communities to infrequently visited campgrounds), it only had a minor effect on the aggregated map cell values and did not change the primary patterns of camper travel behavior. The aggregation process yielded a set of ≈15 000 unique map cells representing network nodes.

### Model development

Briefly, we used the model to perform repeated simulations of camper movement (and thus potential firewood transport) from each location to other locations via the pathways comprising the network. We then compiled these simulation results to estimate the relative risk, *φ_ij_*, of a forest pest being spread by campers from a given location (i.e., map cell), *i*, to another location (cell) of interest, *j*. In turn, the *φ_ij_* output values provided a foundation for further pathways-related analyses.

In this networked set, any two cells *i* and *j* were connected by a unique pathway segment, *ij*. Each *ij* had a value, *m_ij_*, which represented the total number of trips from *i* to *j* over the study period, as well as a value, *m_ji_*, representing the number of return trips from *j* back to *i* over this period. Recall that we have assumed that a constant proportion of all camper travel (i.e., irrespective of direction) involves the movement of infested firewood, and that firewood is the dispersal vector of interest. To structure the model so that it depicted all relevant trips, including relevant return trips from campgrounds, we had to address the possibility that campers might not use all of their firewood upon reaching their destinations. Quantitative data about firewood transport and usage are scarce, especially with respect to wood left unburned after camping trips. We approximated *m_ji_* based on a Wisconsin study [46], which reported that 15% of firewood-carrying campers bring unused firewood home with them. In terms of the network model, this meant adding a complementary return segment, *ji,* for each pathway segment *ij*, with an associated number of trips, *m_ji_* = 0.15*m_ij_*.

Although the principal reason for including these *m_ji_* values was to depict potential forest pest transport in unburned firewood brought back to campers’ homes, aspects of our network model implementation afforded them a somewhat broader role. Because the underlying NRRS data were aggregated at the level of 15 km cells (i.e., each cell incorporated multiple origin and destination points from the initial data set), and because each cell served as both a potential origin and destination location in the model, the *m_ji_* values can be seen as generally enhancing the possibility of secondary transmission of forest pests between map cells. Conceptually, secondary transmission may not just involve the movement of infested firewood from a campground to a camper’s home, but also from one campground to another, or even between communities. Hence, the *m_ji_* values, as implemented in this case, may provide the model with a certain degree of realism. At the same time, the limited evidence for our fundamental assumption (i.e., *m_ji_* = 0.15*m_ij_*) emphasizes that the model output values should be interpreted cautiously.

We incorporated the *m_ij_* and *m_ji_* values into an *n×n* matrix of travel frequencies, **M**, where *n* represents the total number (≈15 000) of unique map elements (15 km×15 km map cells) in the aggregated NRRS data. The non-diagonal elements of **M** were the *m_ij_* and *m_ji_* values for the pathway segments connecting each pair of map cells:
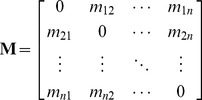
(1)All diagonal elements of **M** (i.e., elements where *i* = *j*) were set to 0. Overall, **M** was a sparse matrix with a large proportion of the non-diagonal elements also set to 0; even after the NRRS data aggregation process, many map cell pairs had no recorded travel between them during the study period, so the number of non-zero *m_ij_* (and *m_ji_*) values was far below the theoretical maximum (≈15 000*≈15 000).

From **M**, we developed another matrix, **P** *_t_*, which depicted, in relative terms, the risk of forest pest (i.e., wood-and bark-boring insect) dispersal via camper travel along the pathway segments during the study period *t*. In estimating the elements of **P** *_t_*, we assumed that *p_ij_*, the risk of dispersal along a given segment *ij*, was linearly related to the frequency of camper travel, i.e., the number of trips from *i* to *j* during *t*:
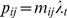
(2)where *λ_t_* is a scaling parameter. Preferably, this parameter would allow us to calculate the total likelihood (i.e., over the study period *t*) of camper transport of infested firewood from *i* to *j* in the matrix. Indeed, knowing the precise value of *λ_t_* would be critical if one wanted to estimate an exact probability of a specific pest being moved from a given *i* to a given *j*. However, we did not need absolute estimates of *p_ij_* to achieve our goal of ranking map locations using the model output values as a relative risk measure. In this case, the only requirement for *λ_t_* was that it was sufficiently small to guarantee that the sum of the values in each row of **P** *_t_* was below 1.

Each row of **P** *_t_* included an additional variable, *p_i_*_ term_, which represented the chance that no camper travel (i.e., to any *j*) would proceed from *i*:
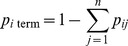
(3)As this equation suggests, if *p_i_*_ term_ was equal to 1 for any row, then the map cell *i* associated with that row did not function as a point of origin in the pathway model. However, this did not preclude the location from functioning as a potential destination *j*.

The matrix, **P** *_t_*, of the relative risk of dispersal by campers traveling along each pathway segment was thus specified as follows:
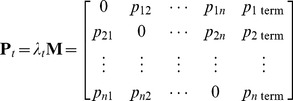
(4)We used **P** *_t_* as the basis for stochastic pathway simulations of camper travel (and the corresponding dispersal risk) from a particular map cell *i* to other map cells in the study area. Setting *i* as the origin location, the model extracted the vector of values associated with *i* (i.e., the values in row *i*) and used them to simulate camper travel from *i* through the network to other map cells. In short, for each *i*, the model used the associated vector of values to select the next map cell. The simulation of an individual trip’s path proceeded until reaching a final destination (i.e., a map cell with no outgoing travel), or instead, when a terminal, no-travel state was selected based on the *p_i_*_ term_ value. The summary risk, *φ_ij_*, of camper-facilitated forest pest dispersal from a given cell *i* to a cell *j* of interest was then estimated as follows:
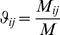
(5)where *M_ij_* is the number of times pathway travel from *i* to *j* was simulated to occur, and *M* is the total number of simulations (*M* = 4×10^6^ in this study). As a proportional measure, *φ_ij_* is bounded between 0 and 1, and is perhaps most easily interpreted as an ordinal score indicating the level of dispersal risk from *i* to *j*.

The large number of repeated model simulations allowed us to represent multi-destination itineraries (i.e., campers traveling to multiple campgrounds before returning home), despite certain data limitations. Multi-destination recreational travel is common in the US and Canada; it may be motivated by such considerations as cost-efficiency or the wider array of recreational opportunities that are available when incorporating several destinations into a single trip [47], [48]. Unfortunately, due to privacy concerns, all personally identifiable information was deleted from the original NRRS reservation records, so we had no documentation of instances where a camper made reservations at multiple campgrounds on consecutive or near-consecutive dates. However, through repeated model simulations, we constructed a comprehensive set of travel paths originating from each map cell. Many of these paths included one or more intermediate destination cells in addition to the final destination map cell. These multi-destination paths were incorporated in the *φ_ij_* values, which summarized all travel paths involving cells *i* and *j*, whether or not the paths actually ended at *j*.

### Applying the network model for analysis

The network model can be used to create maps where any individual location in the potential area of concern can be set as the origin (for forward pathway analysis) or destination (for reverse pathway analysis) location of interest. These types of location-specific maps are useful for analyzing particular invasion scenarios, such as identifying the most likely source locations if an individual map cell was invaded by a pest of interest. For example, Appendix S2 in the Supporting Information presents both forward and reverse pathway analysis maps for the Yosemite Valley area of Yosemite National Park in California. Yet, pathway analysis maps have limited utility for establishing regional-scale management priorities, since they do not provide a comprehensive overview of the invasion risks associated with camper travel (and firewood transport). An extra analytical step is required to summarize the model results in a broader geographic context. Previous work [15], [16], [19], [23] suggests that campers often travel across state or provincial borders. Furthermore, regulatory decision making with respect to invasive species, such as the enactment of firewood restrictions to reduce pest spread, usually takes place at the state or provincial level [49]. Therefore, we developed model outputs that identified those locations outside a state or province of interest that were most strongly linked to locations inside the state or province.

For each US state and Canadian province, we constructed a map where, for each cell *i* outside the target state (province), we summed the model-derived risk values (i.e., the *φ_ij_* values) for all pathways between that cell *i* and any destination cell *j* within the target state (province). Each map thus depicts the most likely external source locations if a camper-associated forest pest were to be found within the state (province) of interest. Note that for the Canadian provinces, we assume that campers returning from the US could still be transporting forest pests despite biosecurity measures that restrict cross-border movement of firewood. A primary purpose of these maps is to identify significant “bottleneck” locations where surveillance, firewood inspections, public awareness campaigns, or other response procedures may be most cost-effective in terms of protecting the state (province) of interest from camper-facilitated forest pest invasions [50].

We created origin risk maps for the 48 contiguous US states as well as seven Canadian provinces bordering the US. We omitted New Brunswick, Prince Edward Island, Newfoundland and Labrador, and the three Canadian territories because the amount of camper travel associated with each was insufficient to generate a meaningful origin risk map. As noted earlier, the *φ_ij_* values shown in the maps should only be interpreted as a relative measure of camper-related pest dispersal risk. However, the maps share a single frame of reference because they were generated using the same underlying pathway matrix, which encompassed the entire study area.

## Results

**Figure 1:**
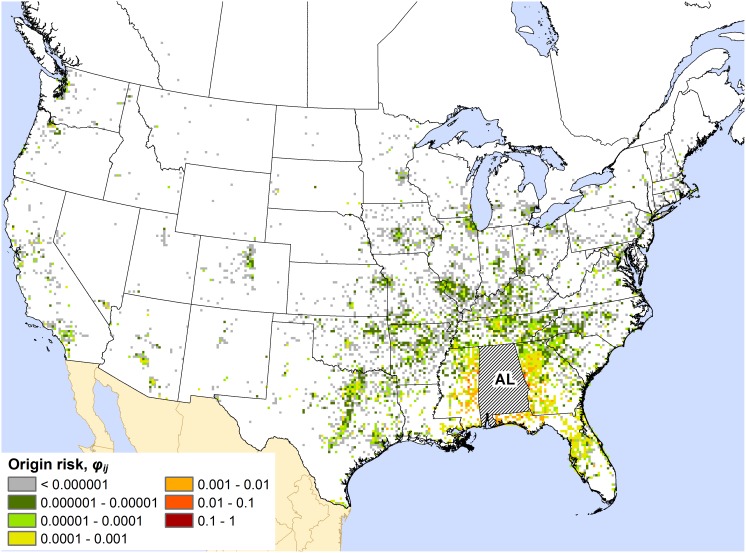
Map of the out-of-state origin risk for Alabama (AL). The color of a map cell indicates the risk, *φ_ij_*, that the cell is the source of any camper-transported forest pest found to be introduced into the state.

**Figure 2:**
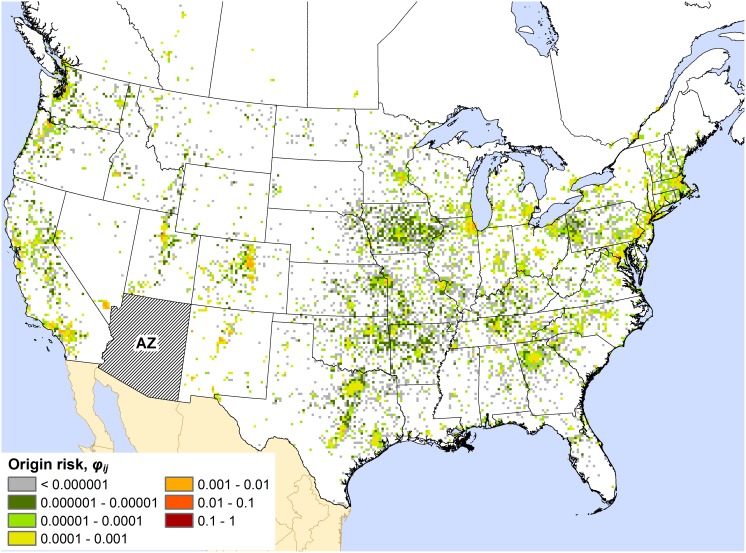
Map of the out-of-state origin risk for Arizona (AZ). The color of a map cell indicates the risk, *φ_ij_*, that the cell is the source of any camper-transported forest pest found to be introduced into the state.

Maps for all states and provinces are presented in Appendices S3, S4, and S5 in the Supporting Information. The maps commonly displayed one of two basic spatial patterns of out-of-state (or out-of-province) origin risk. For many states, as exemplified by the map for Alabama (Figure 1), the highest out-of-state origin risk values (i.e., the summed *φ_ij_* values for a 15×15 km cell) were mostly restricted to a localized zone around the state of interest, which typically consisted of adjacent portions of neighboring states. This spatial pattern was typical for states in the southeastern and northeastern US as well as the Great Lakes region (e.g., Wisconsin, Michigan, and Indiana). Under this pattern, a local zone of origin risk may be perceptible, but the majority of map cells with high origin risk values are associated with major urban areas that are distant from the target state. With respect to Arizona (Figure 2), high-risk cells can be seen in western US cities like Denver (CO), Salt Lake City (UT), and Las Vegas (NV), but also in cities like Chicago (IL), Washington (DC), Boston (MA), and Montreal (QC). This type of dispersed pattern of origin risk was exhibited by several western US states, including California, Colorado, and Utah. Axiomatically, all of these states feature popular recreational destinations (e.g., national parks) that draw visitors from across North America.

**Figure 3:**
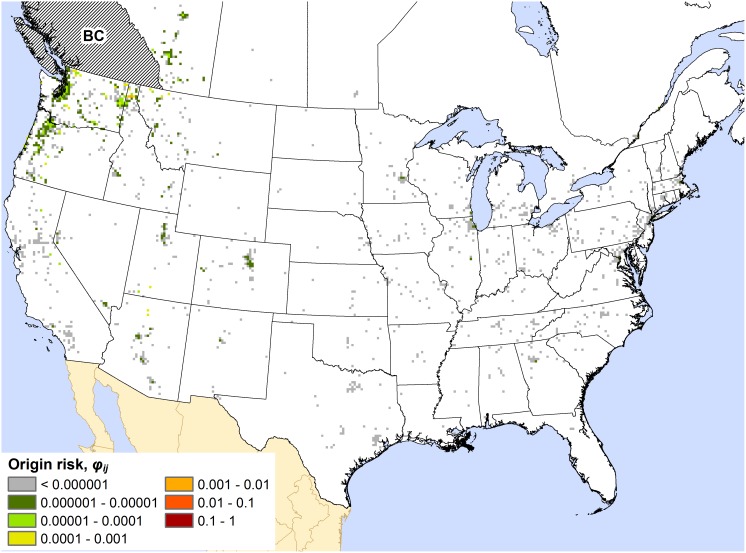
Map of the out-of-province origin risk for British Columbia (BC). The color of a map cell indicates the risk, *φ_ij_*, that the cell is the source of any camper-transported forest pest found to be introduced into the province.

Some states, such as Idaho and Missouri (see Appendix S3), displayed aspects of both spatial patterns: a localized zone of high origin risk around the state of interest, along with several moderate- to high-risk (*φ_ij_*>0.0001) hotspots associated with major urban areas outside this zone. For example, the map for Missouri includes hotspots associated with Chicago, Dallas (TX), and Denver; all three cities are more than 400 km away from the state. The maps for the Canadian provinces also exhibited characteristics of this mixed pattern, although their origin risk values were typically much lower than those for the contiguous US (Appendix S5). Indeed, the maps for less populous Canadian provinces, such as Saskatchewan, featured few map cells with any out-of-province origin risk (i.e., *φ_ij_*>0). However, the map for British Columbia (Figure 3) displays a zone of low to moderate risk extending into Idaho, Oregon, and Washington. In addition, isolated cells with moderate origin risk can be seen elsewhere in the map, usually in association with prominent recreational destinations such as Grand Canyon National Park in Arizona.

## Discussion

It is challenging to account for the myriad factors that influence pest invasions, and more practically, to determine whether the arrival of a species in a particular location will result in establishment of a viable population and subsequent host impact. For instance, a wood-boring insect species transported to a location in a bundle of firewood may already be present there (i.e., it may be native to the area, or a previous invader), and so may not represent a major new forest health risk. Moreover, whether a newly arrived species is alien or not, there are many possible constraints on its long-term establishment, including the suitability of the environment in the species’ new location, the availability of suitable hosts, the species’ life stage, reproductive status, and population size, and the presence of predators [15], [51], [52]. Nevertheless, because of the intense propagule pressure [53] on a potential destination that may be facilitated by human activities such as recreational travel, the risk of successful invasion may still be substantial.

The authorities who must manage this risk typically do not have sufficient resources to implement the measures that may be necessary to detect incipient pest threats, which are fundamentally rare events. Unfortunately, any delay in detecting a new threat – if the pest proves to have a significant impact – will likely lead to increased response costs in the future, such as the greater expense of eradicating a larger invaded area [54], [55], or the greater probability of the population growing too fast for a successful eradication. Prohibitive measures, such as restrictions on the distance firewood can be moved, may slow the expansion of pests of concern, but are difficult to enforce [56]. Public awareness efforts may help promote compliance with such restrictions, but it is impossible to eliminate the risk [56].

Although we cannot remove all risk of forest pest dispersal due to camper travel, the maps generated with our network model can guide the deployment of resources for response activities aimed at managing the risk. The maps represent a novel application of a modeling framework that was previously used to analyze invasion risks associated with global marine shipping [37] and truck-based freight transportation in Canada [39]; the novelty lies in how the maps summarize and depict the model outputs at the level of individual states and provinces. To construct the maps, we ran the network model from every potential origin location (i.e., each map cell) outside a target state or province. In short, we compiled a series of forward-looking analyses from every eligible node in the network to a geographic region of interest (i.e., a state or province), instead of analyzing the results for individual origin-destination node pairs, as in Yemshanov et al. [39]. Perhaps more importantly, because all 55 origin risk maps share a single frame of reference (i.e., were generated using the same pathway matrix), the *φ_ij_* values reported for one state or province are directly comparable to the *φ_ij_* values for any other state or province. This comparability may make the maps useful for broad-scale decision making (e.g., determining the proportion of total response resources that should be allocated to each of several neighboring states).

### Decision making implications of the observed risk patterns

The distinct patterns observed in the origin risk maps suggest that no single response strategy may work for all states and provinces. For example, the origin risk map for Alabama (Figure 1) suggests that most of the campers coming from outside the state originate in adjacent areas of neighboring states like Georgia and Mississippi; thus, most out-of-state camper-transported firewood is sourced locally. These campers are probably more familiar with the region’s geography and local road network than campers from farther away. So, when traveling to campgrounds in the neighboring state (i.e., Alabama), they are probably less restricted to interstate highways or other primary roads. If one were attempting to establish a surveillance or firewood inspection campaign in such a region, an area-distributed approach that targeted a variety of locations (e.g., gas stations, grocery stores, and home and garden stores, in addition to campgrounds) throughout the region might work better than exclusively targeting locations along interstate highways or other high-traffic routes. Furthermore, wide distribution of public awareness materials throughout the region might be necessary for those materials to reach their intended audience of potential campers (e.g., roadside billboards about firewood and forest pests, as well as advertisements on television, radio, and social media).

In contrast, the origin risk map for Arizona (Figure 2) suggests that an area-distributed response strategy may not be practical for that state. Compared to Alabama, much more of the firewood coming into Arizona from out-of-state probably originates a long distance away. This may reduce the effectiveness of area-based surveillance or inspection schemes designed to detect emerging pest problems at a regional scale. It may also make coordinating public awareness efforts with neighboring states less effective. Consequently, for Arizona and similar states or provinces, it might be expedient to target locations (e.g., truck stops, rest areas) near interstate highways and other major roads, since these routes could essentially represent bottlenecks in the movement of campers and firewood. Another option might be to direct efforts toward the most heavily visited campgrounds, so response actions are focused on specific destinations rather than a large and diffuse set of likely origin locations. A state or province facing such circumstances might also consider joining with other states or provinces in national-scale public awareness efforts, which may lack the immediate impact of local- or regional-level efforts but are still likely to provide some benefit.

A few broadly operating factors might help explain the observed patterns of origin risk. Within the context of our model, any factor that substantially affected the number of people traveling to (and from) particular locations likely had some influence on the observed patterns. For instance, because the network model was bi-directional (i.e., it also simulated return travel by campers), the human population of a target state or province probably influenced the *φ_ij_* values depicted in its origin risk map, even though these values corresponded to cells outside the state or province. Indeed, we did see evidence of this: the origin risk maps of heavily populated states and provinces tended to display a greater range of *φ_ij_* values, and higher values overall, than sparsely populated states and provinces. Another potentially influential factor was the extent of the recreational opportunities available in a target state. States containing a small number of highly popular recreational destinations, such as Arizona (Figure 2), seemed to exhibit a dispersed pattern of origin risk, while states with many but comparatively low-traffic recreational destinations, such as Alabama (Figure 1), typically exhibited a more localized pattern. Admittedly, our interpretations of the roles of these factors are conjectural. Quantitative analysis of these and other possible explanatory drivers could be a fruitful direction for future work.

### Limitations of the analysis

In implementing the network model, we adopted two simplifying assumptions that merit further discussion. Firstly, we assumed that all visitors to campgrounds, regardless of where they originated, were equally likely to be transporting infested firewood. This may seem like a questionable assumption, since one might expect that visitors traveling to campgrounds in adjacent states are more likely to be transporting firewood than campers who travel across several states to reach their destinations. Indeed, some longer-distance campground visits could involve air travel, which would almost certainly prevent a visitor from bringing firewood from home. Nonetheless, because of the relative remoteness of the campgrounds from urban (or rural) settlements, we can confidently infer that virtually all visitors documented in the NRRS data – even those who may have flown part of the way – arrived at their destination campgrounds via car or some other passenger vehicle, and thus had some opportunity to bring firewood with them. Certainly, any camper bringing firewood into a campground could have procured the wood locally rather than transporting it from home or another distant location, thereby greatly reducing the chance of introducing a truly alien forest pest. Unfortunately, the NRRS data do not provide information about firewood transport and usage, so we cannot quantify the likelihood of local firewood acquisition for any class of camper. Given our data restrictions, it seemed appropriate to assign all visitors an equal likelihood of transporting infested firewood.

Secondly, in making the assumption of equal probability of carrying firewood, we also implicitly assumed that all campers who transported firewood carried roughly the same quantity of wood, and that a constant proportion of this wood was infested. Both assumptions are debatable. For instance, campers traveling to higher-elevation destinations might be expected to bring more firewood than the average camper because they are likely to encounter colder weather. With respect to the infested firewood proportion, it may be worth noting that wood infestation rates can vary substantially between different insect species. For example, Haack et al. [57] estimated that a single infested log of moderate size (≈1 m in length and ≈10 cm in diameter) could contain 100–250 individual bark beetles, but only 20–30 buprestids (e.g., *Agrilus* species) or 5–10 cerambycids (e.g., *Anoplophora* species). This has important implications for successful establishment; a given bundle of infested firewood is far more likely to support a sufficient population of bark beetles than of large borers.

Ultimately, we adopted the aforementioned assumptions because we felt they allowed us to provide meaningful information about camper-related dispersal risk for decision makers, even if that information was only in relative, generalized terms. Of course, our study falls short of being a true probabilistic assessment of the risk of pest dispersal in camper-transported firewood. As illustrated by Haack et al. [57], this risk is at least partially species-dependent. A rigorous probabilistic assessment would therefore require that we focus on one actual species of interest, rather than an unspecified number of hypothetical pests from the broad category of wood- and bark-boring insects. Focusing on one species would permit us to incorporate certain factors into our modeling efforts: the species’ current geographic distribution, including whether campers are traveling to or from an already infested region, as well as the impacts of the current regulatory climate for the species. The geographic distribution of potential hosts would also be relevant. Nevertheless, the data requirements for such an analysis would be substantial. In particular, we would need more specific data on both campers (e.g., the true, distance-dependent proportion of campers that bring wood with them) and firewood (e.g., the real infestation rate of the species of interest) than is available from existing studies [15], [20], [23].

Beyond these assumptions, we should also note that the NRRS data underlying our network model only included visits to federal recreational facilities. We have assumed these data are a reasonable sample of all camper travel, and indeed, we believe these data provide a realistic depiction of the long-distance movements of campers. Nevertheless, our output origin risk maps could change were we to include data from state, provincial, or private campgrounds. In particular, such data might increase the proportion of local, short-distance trips to lesser-known campgrounds in the model. In future work, we will evaluate how camper travel patterns differ when the network model includes state- and provincial-level reservation data in addition to the NRRS data.

Finally, there were computational limitations imposed by the network modeling approach. The aggregation of the NRRS data at the level of 15×15 km cells allowed us to execute a sufficiently large number of pathway simulations within a reasonable amount of computing time. However, through further optimization of the network model and parallelization of the simulations, it would be possible to increase the size of the transmission matrix and run the model at a finer spatial resolution more comparable to the actual resolution of the NRRS data. Note that improving the spatial resolution would not substantially alter the general patterns of camper travel behavior that we described here.

## Conclusions

The network modeling approach offers definite advantages over kernel-based spread models with respect to camper travel and the potential dispersal of forest pests in camper-transported firewood. For instance, a major limitation of the NRRS data set is that we were unable to determine whether a camper made reservations at multiple campgrounds on consecutive or near-consecutive dates (i.e., booked a multi-stop itinerary). When we previously developed dispersal kernels from the NRRS data [16], we were forced to presume that these multi-stop itineraries had a minimal effect on the dispersal distance estimates. The network modeling approach helps us overcome this limitation: through repeated simulations of all plausible pathways in the network, it is possible to identify “secondary origin” points that could represent locations visited during multi-stop itineraries.

Overall, a network modeling approach is practical for some human-mediated invasions because it emulates the vector-based fashion in which these invasions often proceed. Despite its likely shortcomings, a basic network model can provide useful broad-scale information to support invasive species management decisions. Increased availability of such methods will ultimately permit decision makers to shift away from biosecurity strategies focused on controlling pests at jurisdictional borders. Instead, they will be able to implement potentially more productive, pathway-based biosecurity strategies that seek to identify those geographic locations most pivotal to the spread of alien species [31], [58].

## Supporting Information

Appendix S1 **Estimating geographically variable start and end dates for the late spring-early summer period.**
(DOCX)

Appendix S2 **Example forward and reverse pathway analysis maps for the Yosemite Valley area of Yosemite National Park.**
(DOCX)

Appendix S3 **Out-of-state origin risk maps for 24 US states: Alabama – Montana.**
(PDF)

Appendix S4 **Out-of-state origin risk maps for 24 US states: Nebraska – Wyoming.**
(PDF)

Appendix S5 **Out-of-province origin risk maps for seven Canadian provinces: Alberta, British Columbia, Manitoba, Nova Scotia, Ontario, Quebec, and Saskatchewan.**
(PDF)
