# Supplementary material for: Using a Network Model to Assess Risk of Forest Pest Spread via Recreational Travel
Source: PLoS One. 2014 Jul 9;9(7):e102105. doi: 10.1371/journal.pone.0102105 (PMC4090238; doi:10.1371/journal.pone.0102105)
Supplement: Appendix S1 — Estimating geographically variable start and end dates for the late spring-early summer period. (DOCX) [file pone.0102105.s001.docx]

**Using a Network Model to Assess Risk of Forest Pest Spread via Recreational Travel -** Frank H. Koch, Denys Yemshanov, Robert A. Haack, Roger D. Magarey

**Appendix S1. Estimating geographically variable start and end dates for the late spring-early summer period.**

In this study, we focused on the late spring-early summer season under the assumption that this time period would represent the best opportunity for wood- and bark-boring insects to emerge from firewood and become established in the vicinity of the campground where the firewood was transported, or if campers brought infested firewood back with them, near their homes. Effectively, we also assumed that any infested firewood brought to a campground at an earlier date (i.e., prior to late spring) was burned before insect emergence. We believe this is a reasonable assumption since most campers can be expected to use all of their firewood or (perhaps to avoid the future expense of procuring firewood of sufficient quality) take home any leftover wood [1,2]. It also seems likely that any wood that they did choose to leave behind at a campground would be quickly used by subsequent campers. In initial tests with the network model, we found that camper travel patterns based only on late spring-early summer reservations were fairly well correlated with patterns based on all reservations, downplaying the significance of this assumption; see Figure 1 in Koch et al. [3] for example model outputs generated using the full set of reservation records.

To depict geographic variation in the start and end dates defining the late spring-early summer period, we began with a map of the 30-year (1961-1990) mean annual (Julian) date of the last spring frost [4]. However, the date of last frost is an imperfect marker of the beginning of spring, especially since some locations in our study area (e.g., the extreme southeastern US) commonly have years with no spring frost. We transformed the mean last-frost map into a simple phenological map of late spring using a linear function that, based on latitude, incrementally added a number of days to the mean last-frost date. This yielded a map where the start of late spring ranged from a low of 46 Julian days (in southern portions of Florida, Texas, and Arizona) to a high of 225 Julian days (in portions of the northern Rocky Mountains); these date estimates are generally consistent with existing research on plant and insect phenological patterns in North America [5-10]. In turn, we estimated the late spring-early summer period as lasting 70 days from this modified start date. We then used this map to identify a seasonal subset of NRRS reservation records.

**References**

1. Peterson K, Diss-Torrance A (2012) Motivation for compliance with environmental regulations related to forest health. J Environ Manage 112: 104-119.

2. Peterson K, Nelson EB (2009) Firewood use in Wisconsin state parks and forests: 2006 and 2008. Research/Management Findings, Issue 61. Madison, WI: Wisconsin Department of Natural Resources, Bureau of Science Services. 4 p.

3. Koch FH, Yemshanov D, Haack RA (2013) Representing uncertainty in a spatial invasion model that incorporates human-mediated dispersal. NeoBiota 18: 173-191.

4. Daly C, Gibson W, Taylor G (2000) United States mean extreme date of last 32F temperature in spring, 1961-1990. Digital raster map at 1.25 arc-minute (~2 km) spatial resolution. Corvallis, OR: Climate Source, LLC.

5. Beaubien EG, Freeland HJ (2000) Spring phenology trends in Alberta, Canada: links to ocean temperature. Int J Biometeorol 44: 53-59.

6. Gray DR (2004) The gypsy moth life stage model: landscape-wide estimates of gypsy moth establishment using a multi-generational phenology model. Ecol Modell 176: 155-171.

7. Jenkins JP, Braswell BH, Frolking SE, Aber JD (2002) Detecting and predicting spatial and interannual patterns of temperate forest springtime phenology in the eastern U.S. Geophys Res Lett 29: 2201.

8. Régnière J, Bolstad P (1994) Statistical simulation of daily air temperature patterns in eastern North America to forecast seasonal events in insect pest management. Environ Entomol 23: 1368-1380.

9. White MA, De Beurs KM, Didan K, Inouye DW, Richardson AD, et al. (2009) Intercomparison, interpretation, and assessment of spring phenology in North America estimated from remote sensing for 1982–2006. Glob Chang Biol 15: 2335-2359.

10. Wolfe D, Schwartz M, Lakso A, Otsuki Y, Pool R, et al. (2005) Climate change and shifts in spring phenology of three horticultural woody perennials in northeastern USA. Int J Biometeorol 49: 303-309.
