# Supplementary material for: Using a Network Model to Assess Risk of Forest Pest Spread via Recreational Travel
Source: PLoS One. 2014 Jul 9;9(7):e102105. doi: 10.1371/journal.pone.0102105 (PMC4090238; doi:10.1371/journal.pone.0102105)
Supplement: Appendix S2 — Example forward and reverse pathway analysis maps for the Yosemite Valley area of Yosemite National Park. (DOCX) [file pone.0102105.s002.docx]

**Using a Network Model to Assess Risk of Forest Pest Spread via Recreational Travel -** Frank H. Koch, Denys Yemshanov, Robert A. Haack, Roger D. Magarey

**Appendix S2. Example pathway analysis maps for the Yosemite Valley area of Yosemite National Park.**

The two maps shown in Figure S2 describe the degree of connectivity, in terms of camper travel, between the Yosemite Valley and other locations in the study region. Note that “location” in this case refers to the 15 km × 15 km area represented by a map cell, rather than a single specific campground or populated place.

**
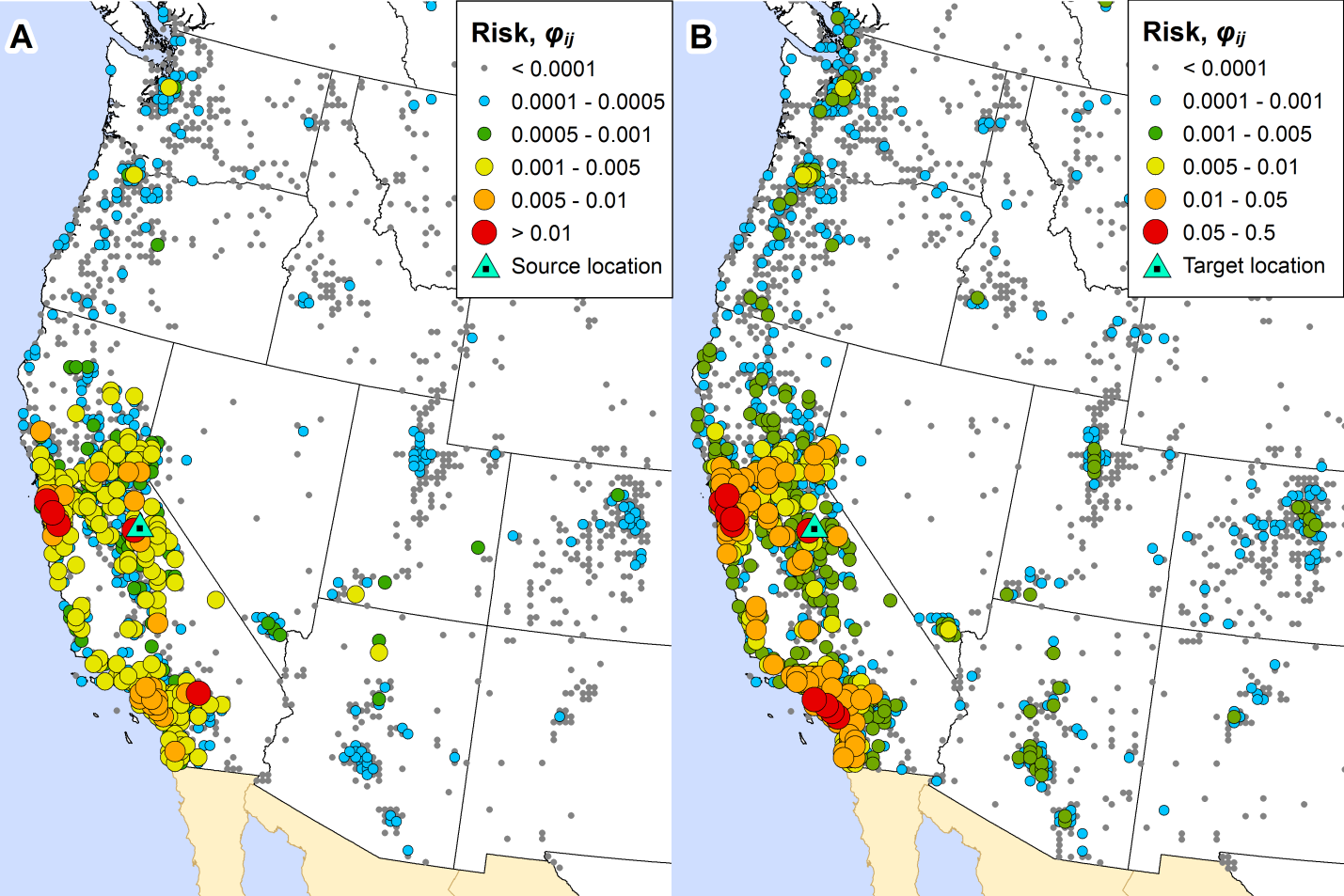
**

**Figure S2. Pathway analysis maps for Yosemite Valley.** A: forward pathway analysis; B: reverse pathway analysis.

The forward pathway analysis map (Figure S2A) highlights those locations that are most at risk of receiving a forest pest were it to be transported from the Yosemite Valley by campers, presumably in infested firewood. Other than the nearby location of Wawona (also within Yosemite National Park) as well as a location near San Bernardino in southern California, the likeliest destinations for a hypothetical forest pest from the Yosemite Valley are clustered in the San Francisco area of northern California. In fact, most of the potential destination locations with at least moderate levels of risk (*ϕ_ij_* > 0.001) are in California, although locations in Seattle (WA) and Portland (OR) also exhibit moderate risk values, as do locations corresponding to Grand Canyon (AZ) and Zion (UT) National Parks.

Alternatively, the reverse pathway analysis map (Figure S2B) highlights the locations that are the most probable sources if the Yosemite Valley area were found to be infested by a camper-transported forest pest. The likeliest origin locations are all within California, especially in the Los Angeles and San Francisco urban areas. However, locations in Seattle, Portland, and Las Vegas (NV) also display moderate (0.005 < *ϕ_ij_* ≤ 0.01) origin risk values.
