# Supplementary figures and images for: Using a Network Model to Assess Risk of Forest Pest Spread via Recreational Travel
Source: PLoS One. 2014 Jul 9;9(7):e102105. doi: 10.1371/journal.pone.0102105 (PMC4090238; doi:10.1371/journal.pone.0102105)

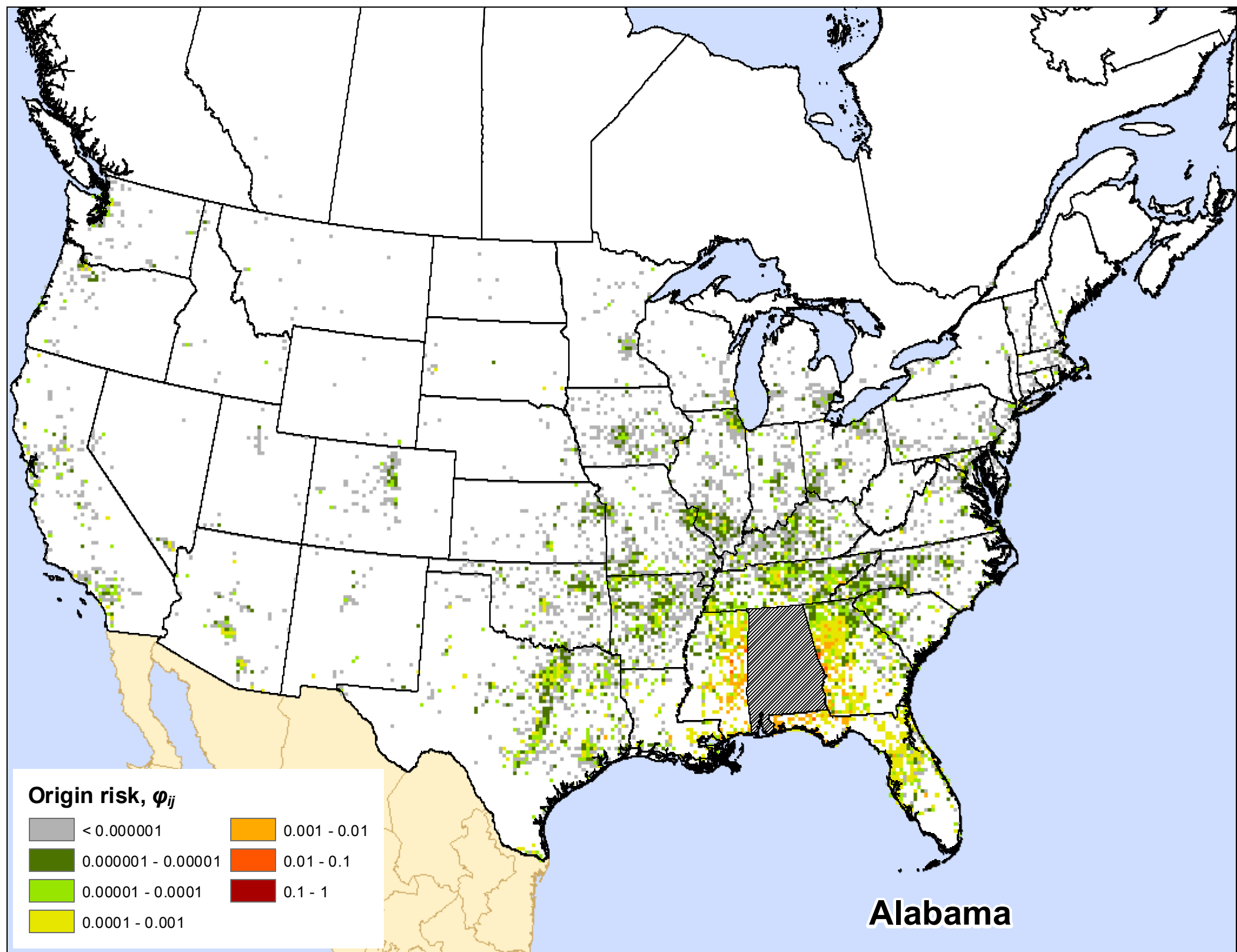

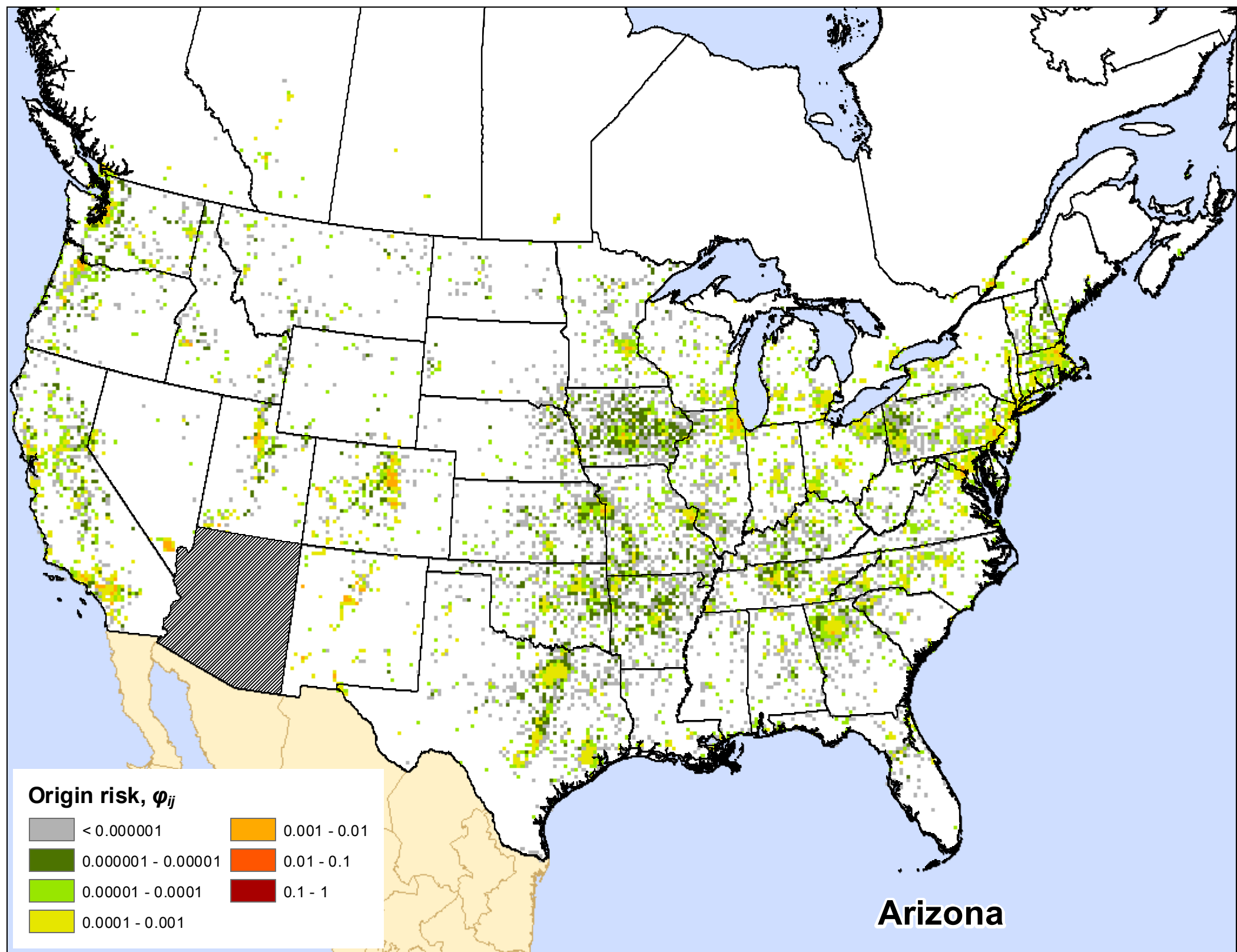

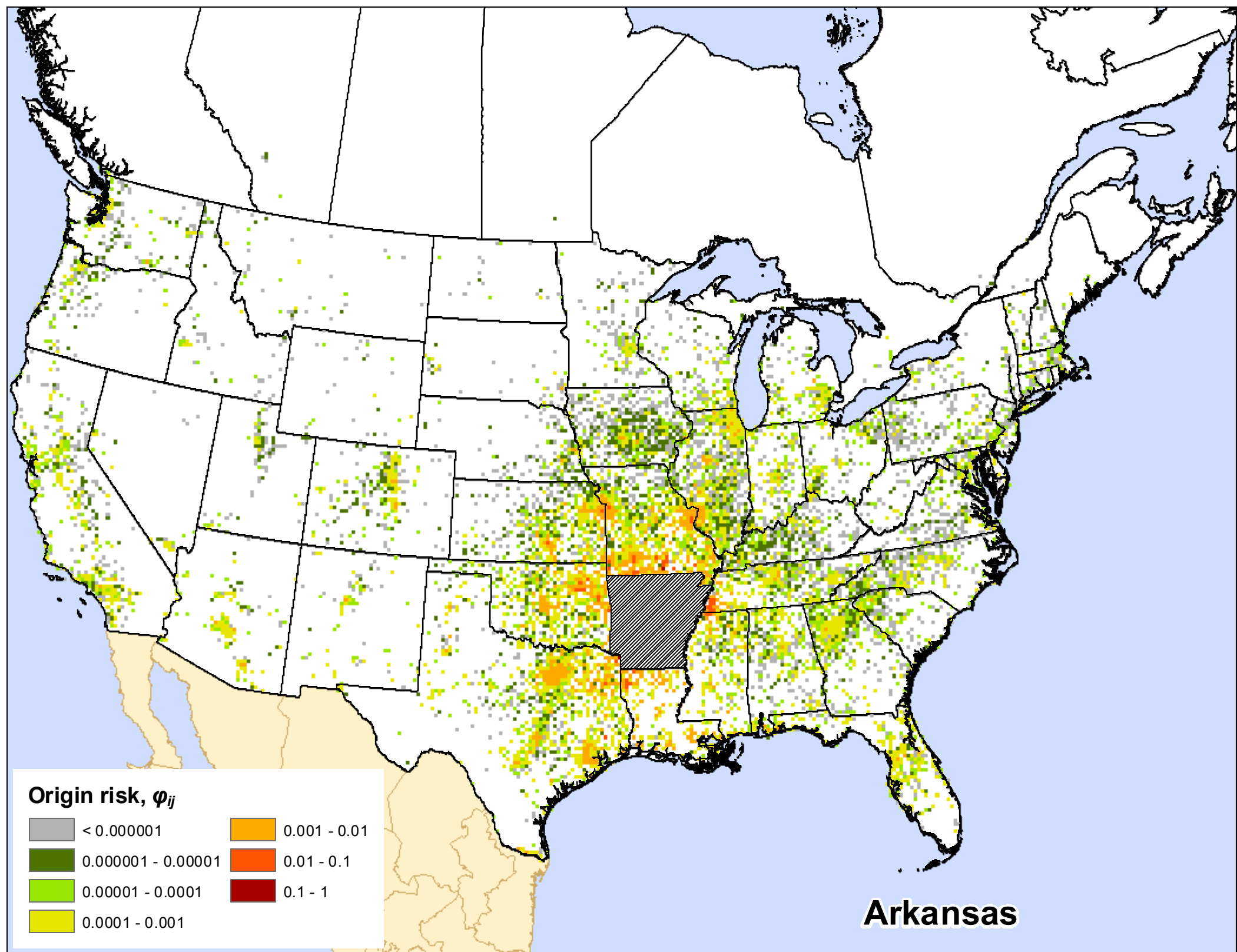

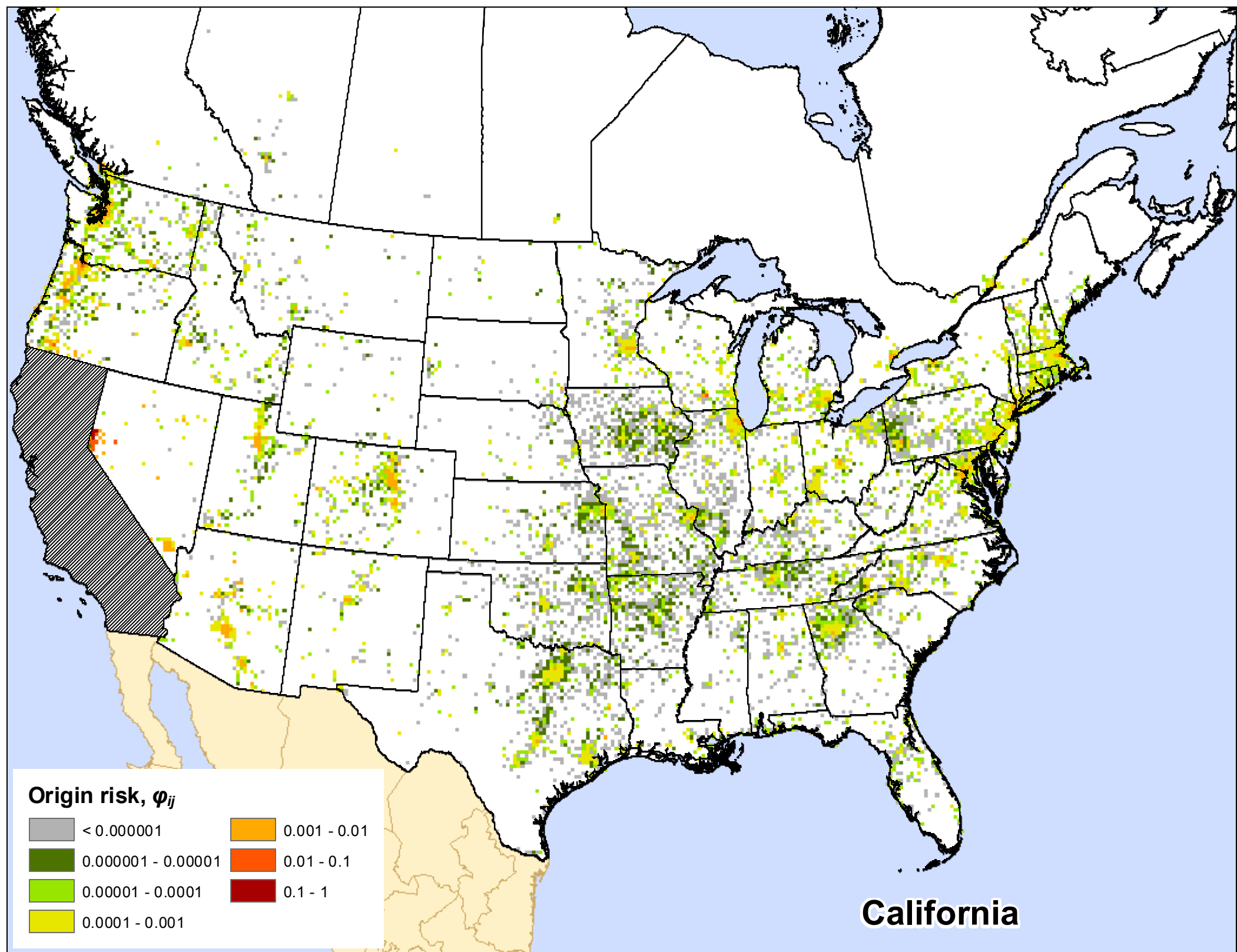

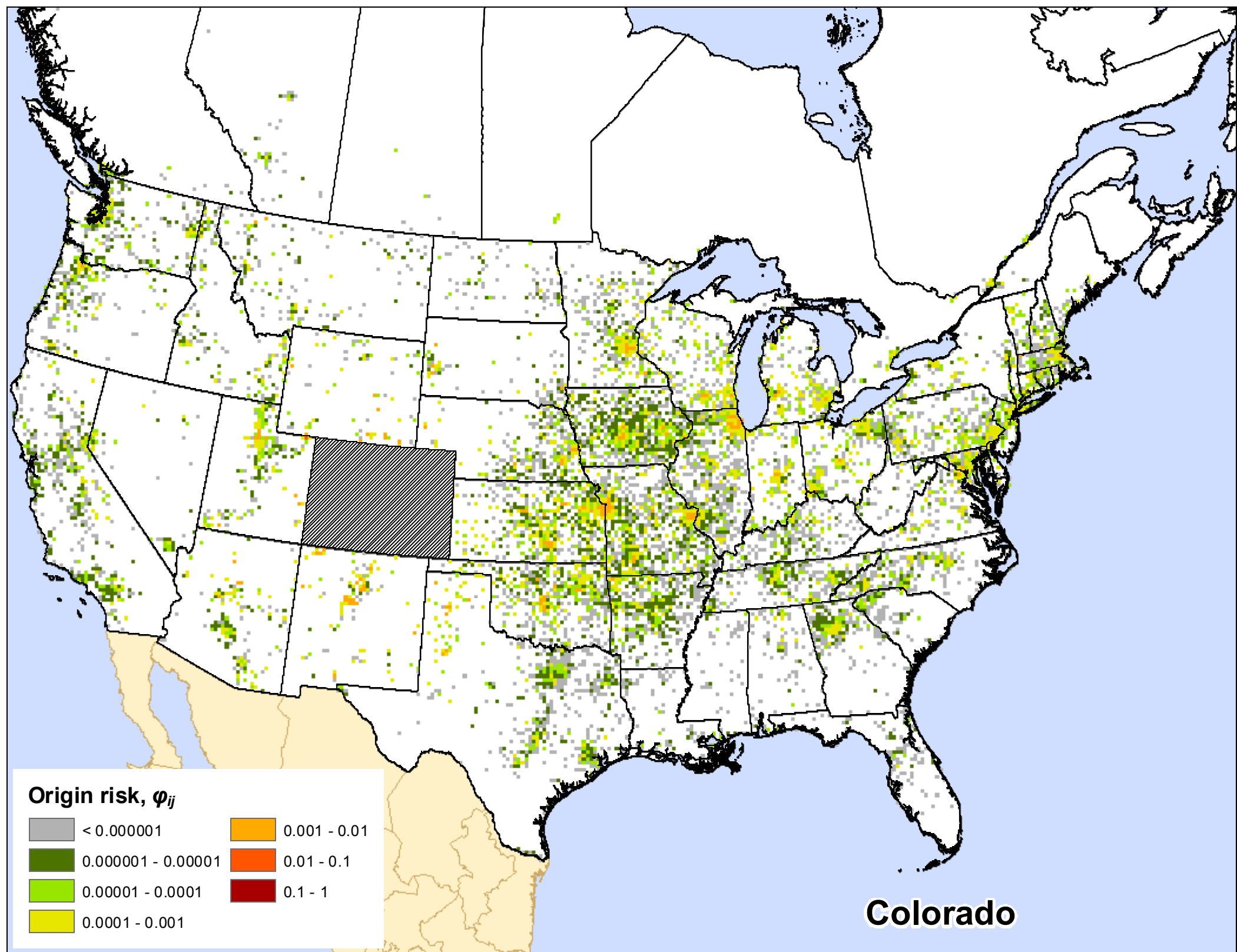

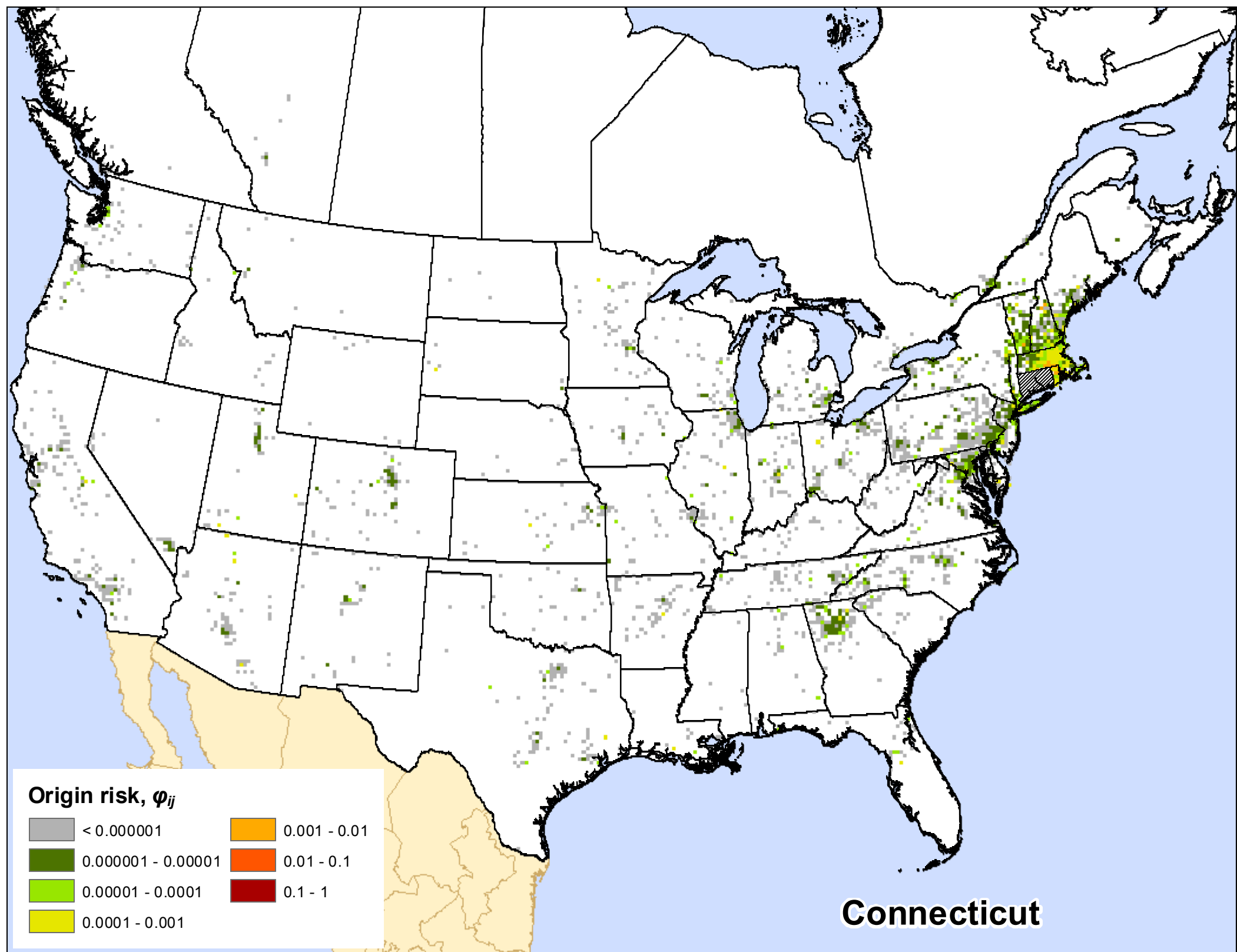

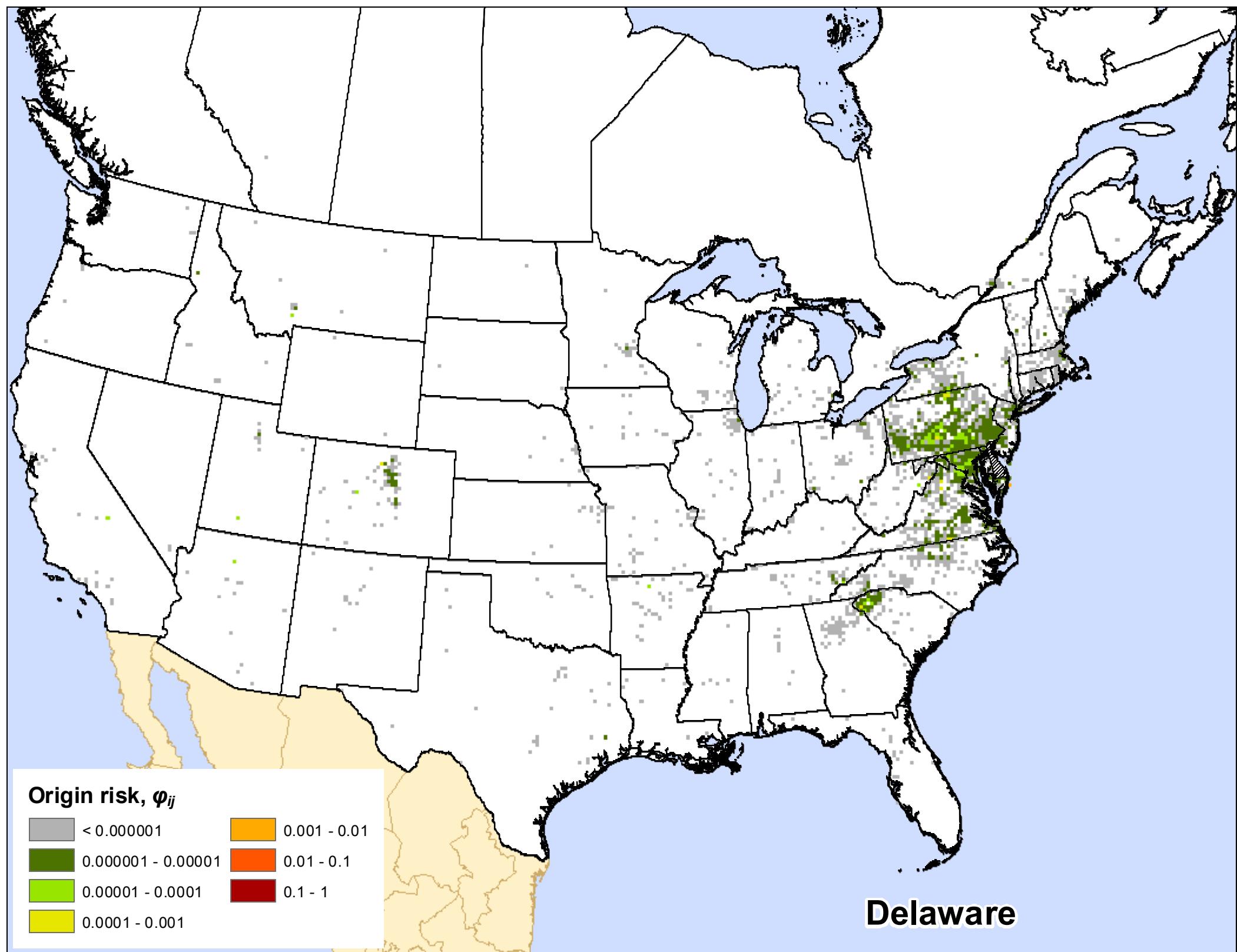

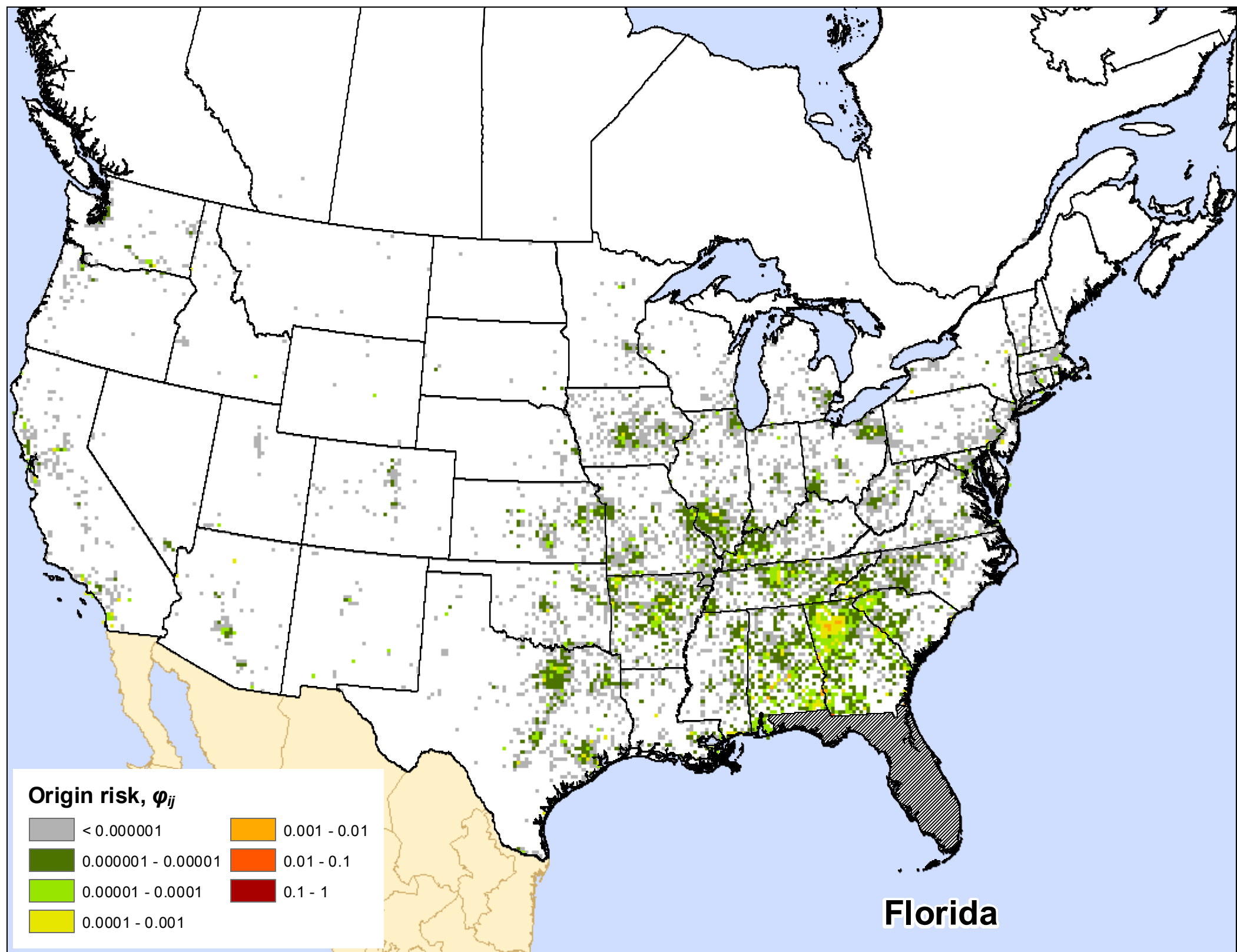

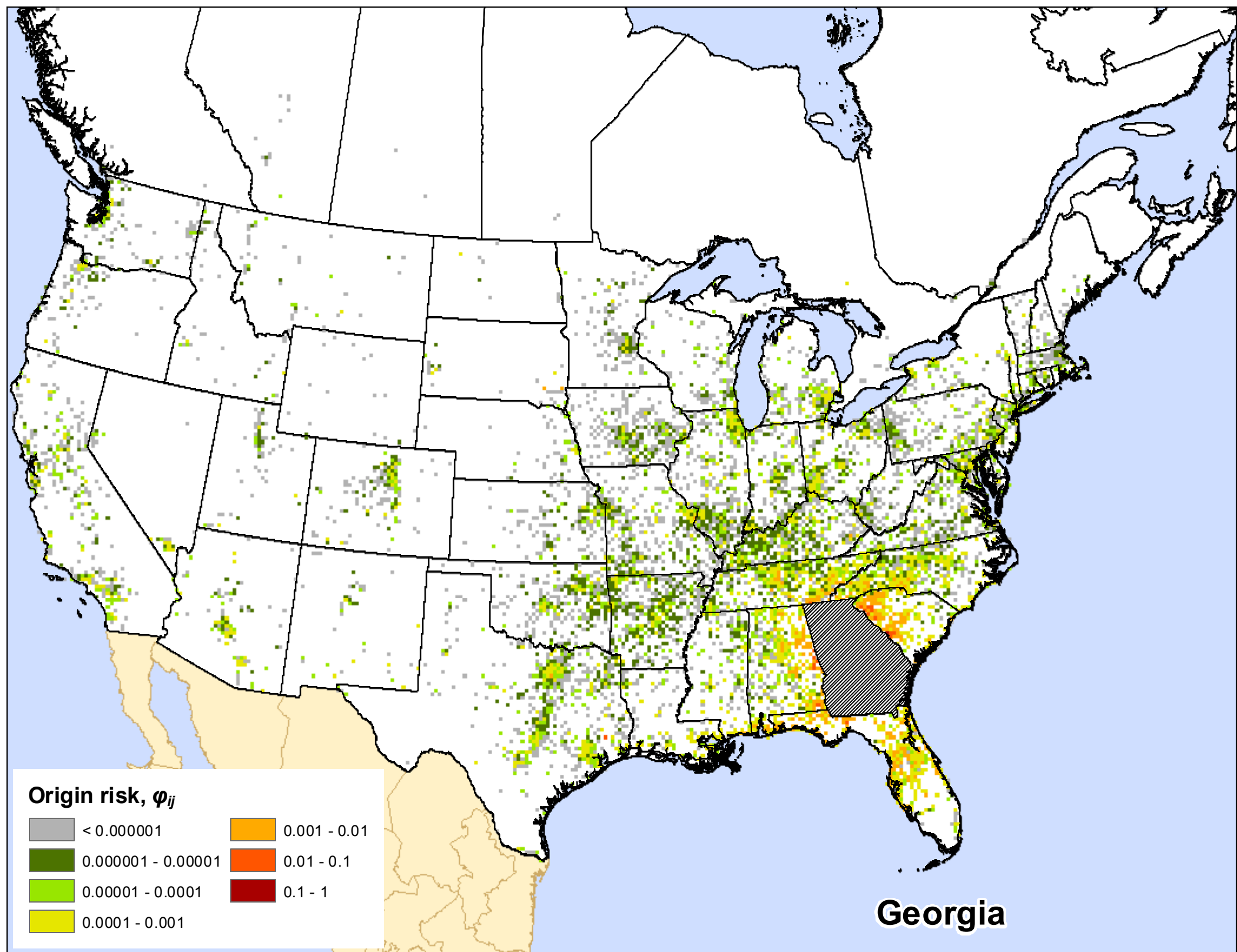

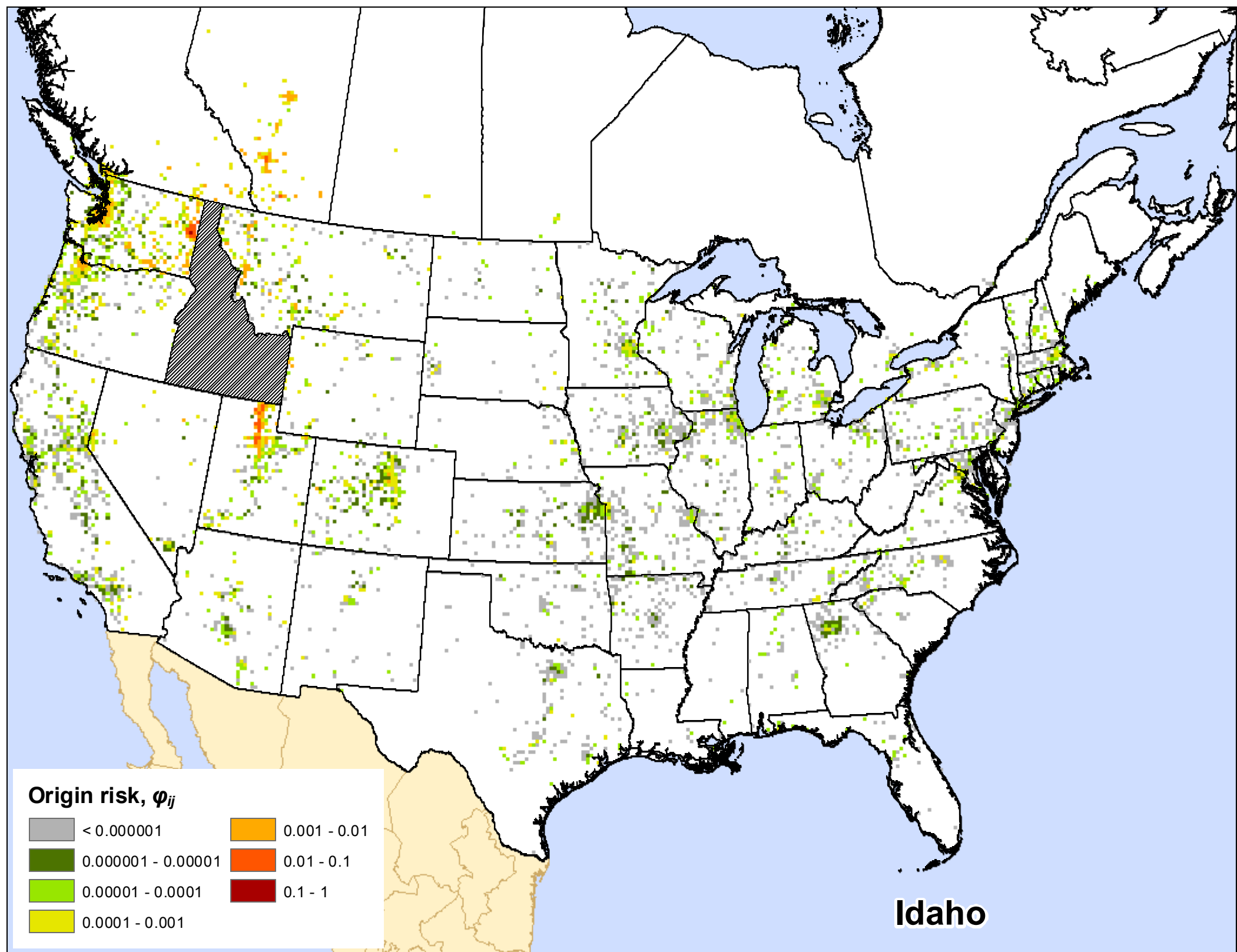

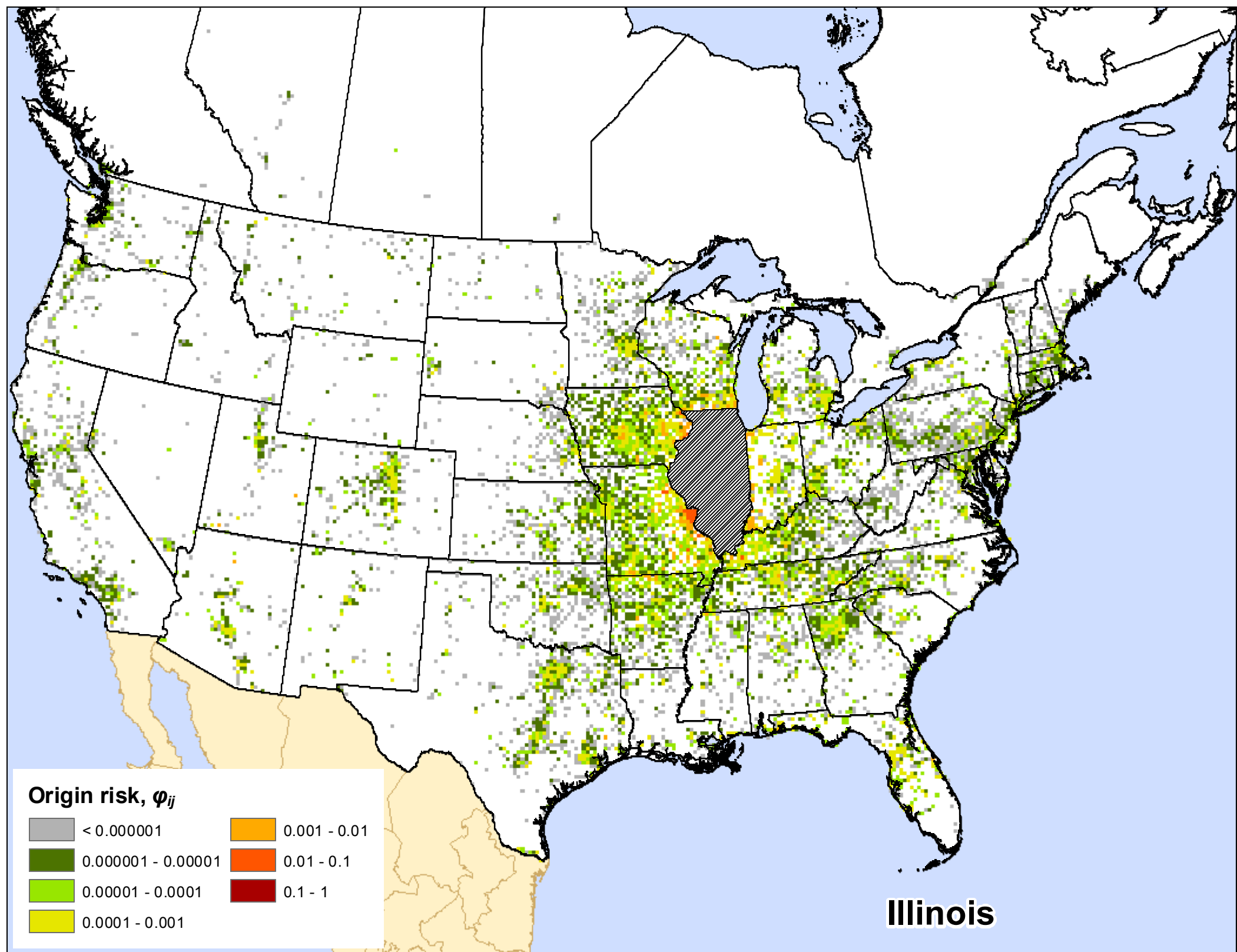

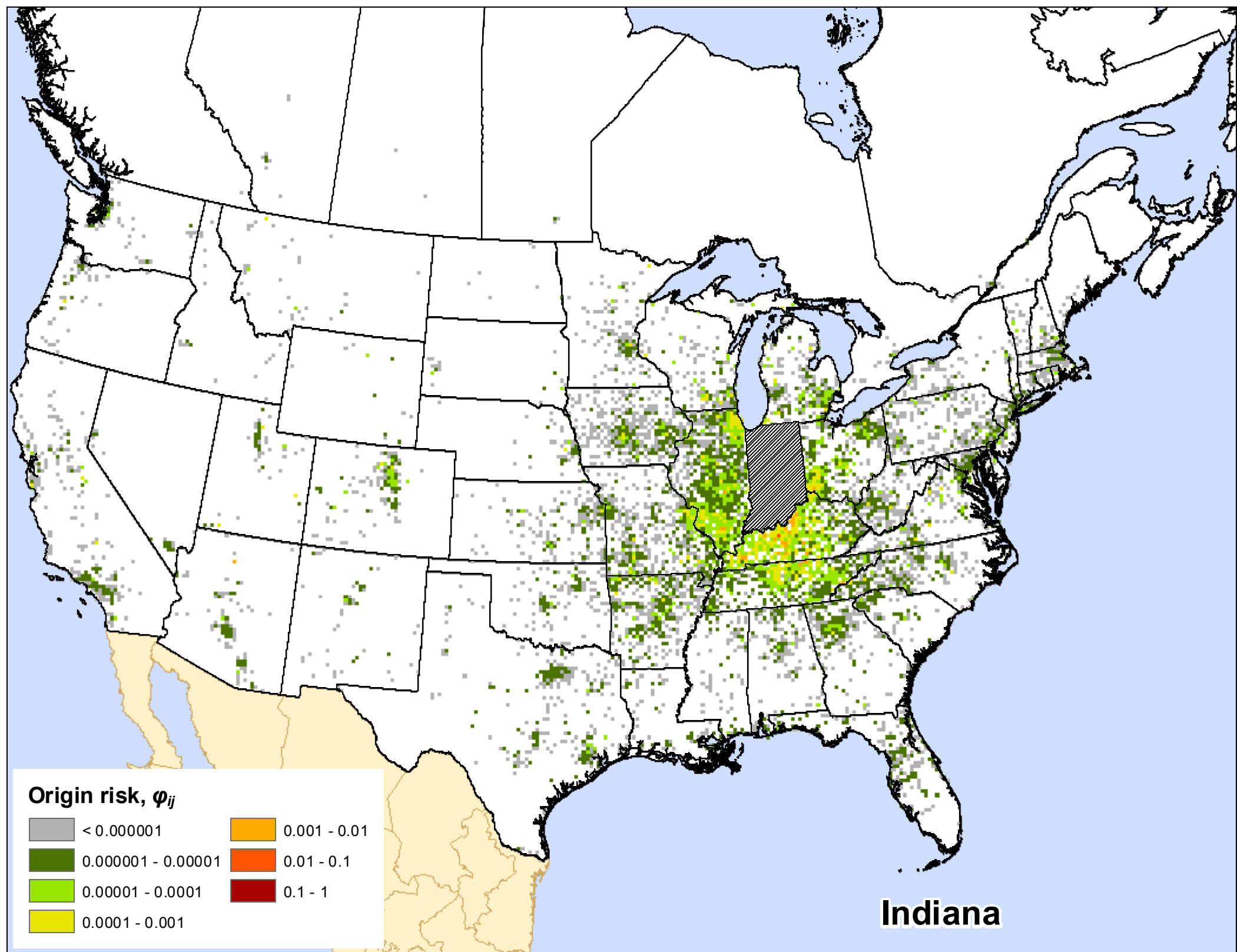

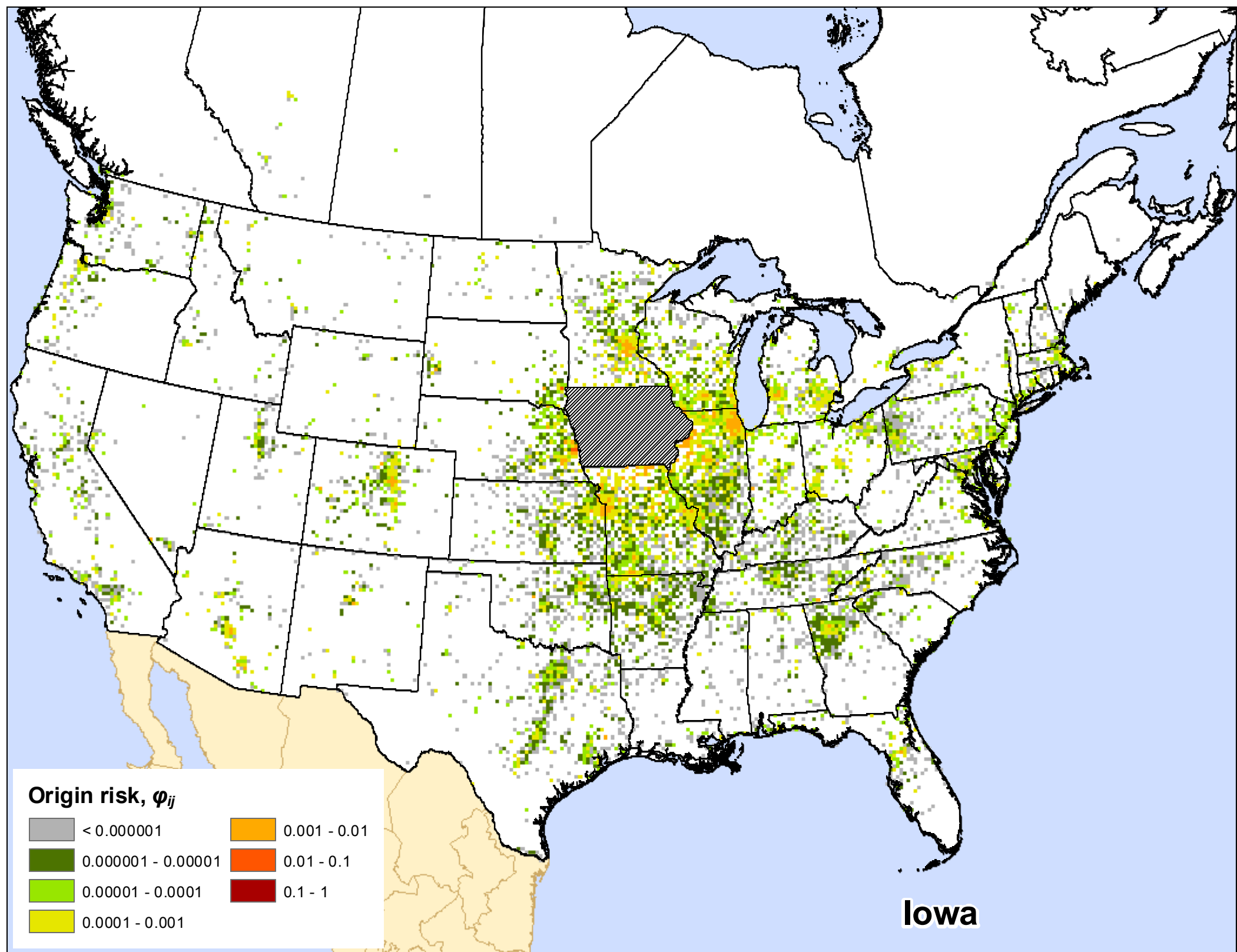

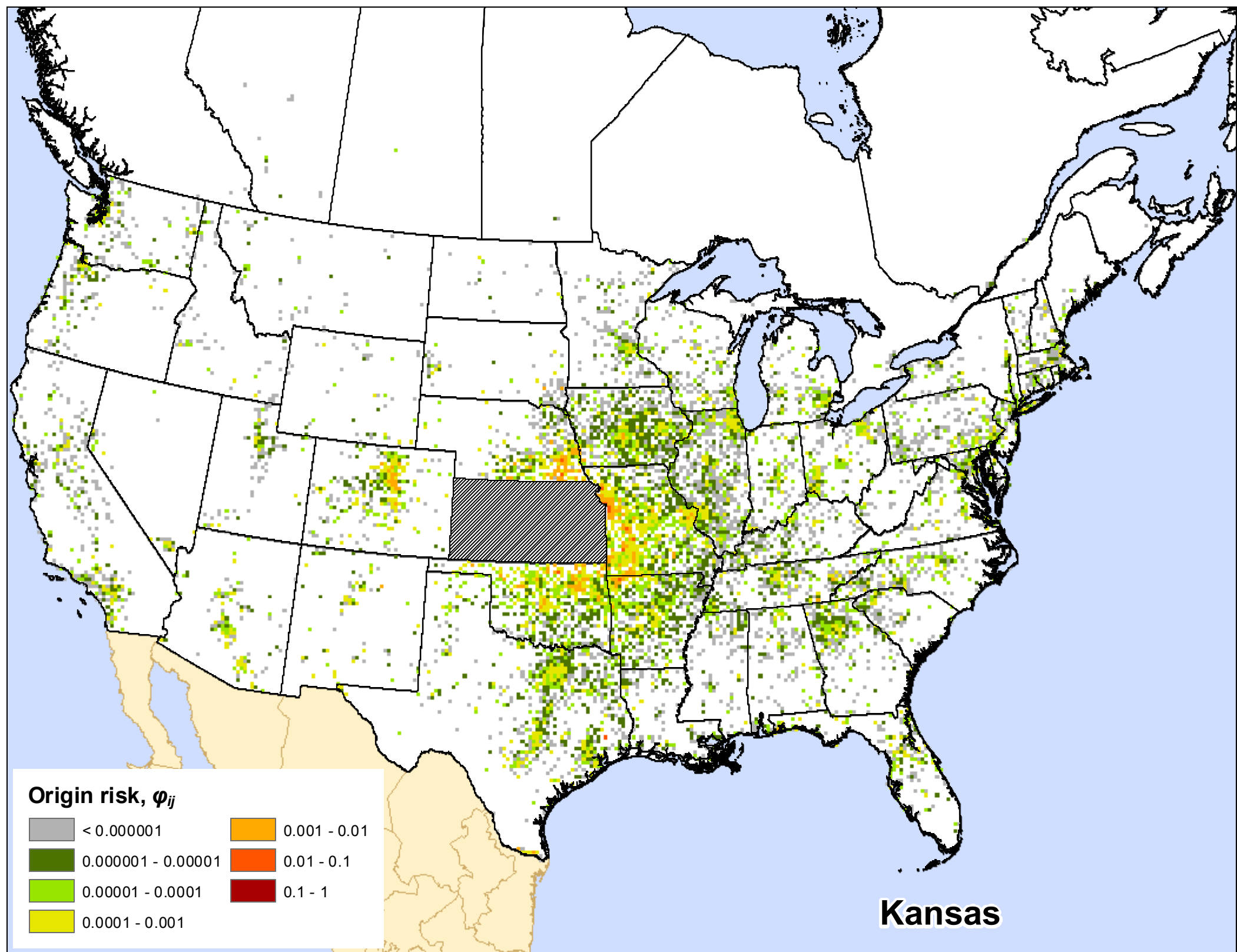

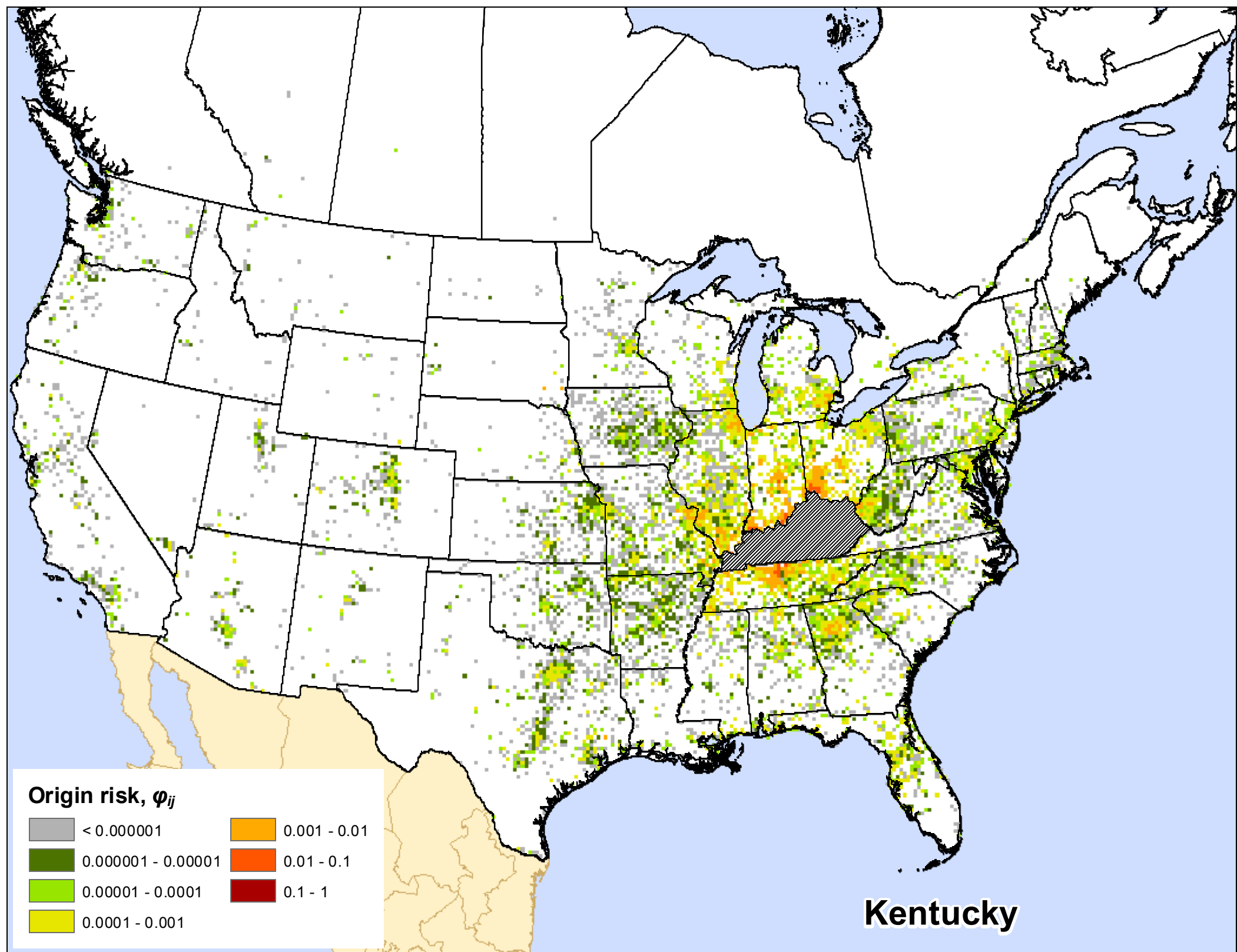

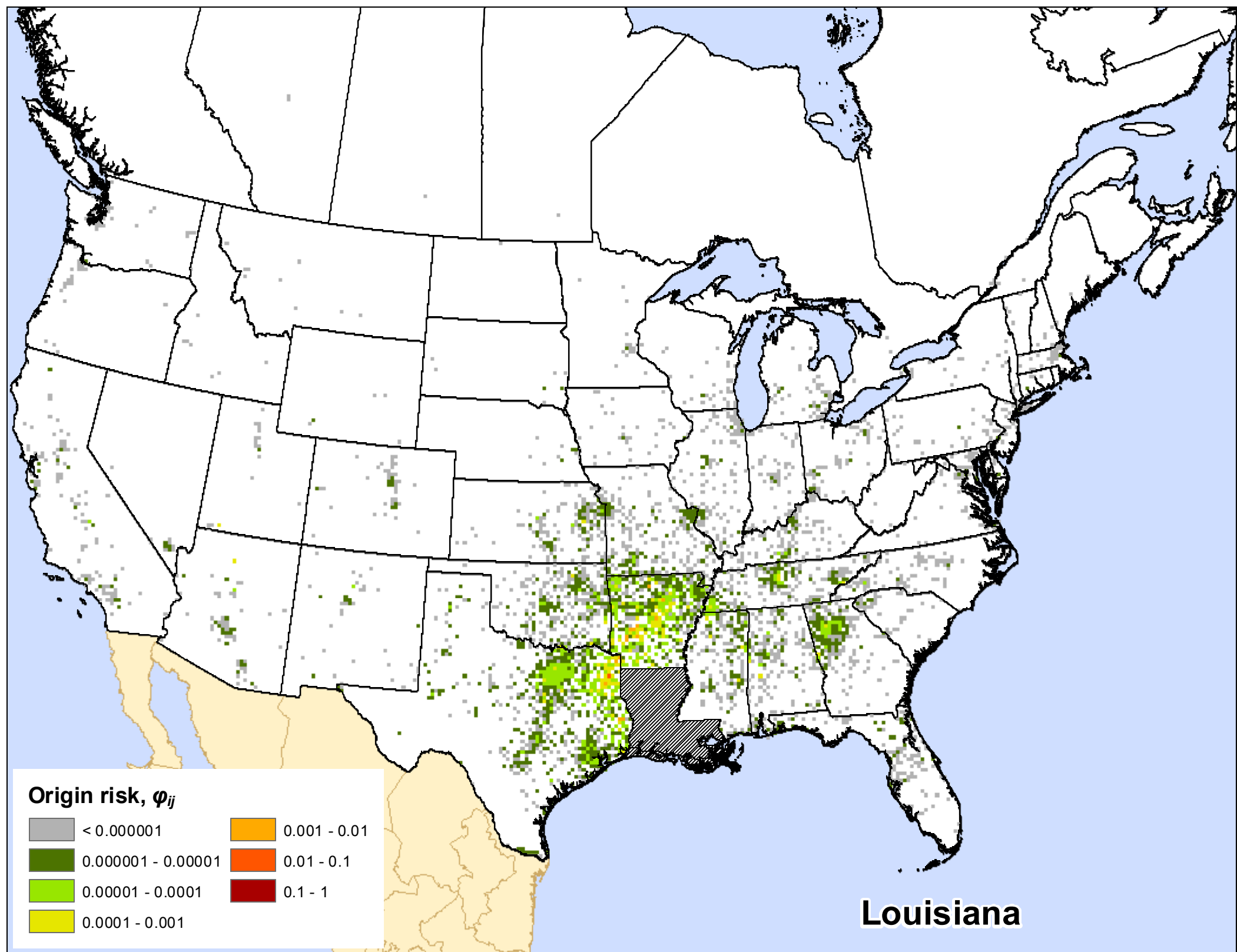

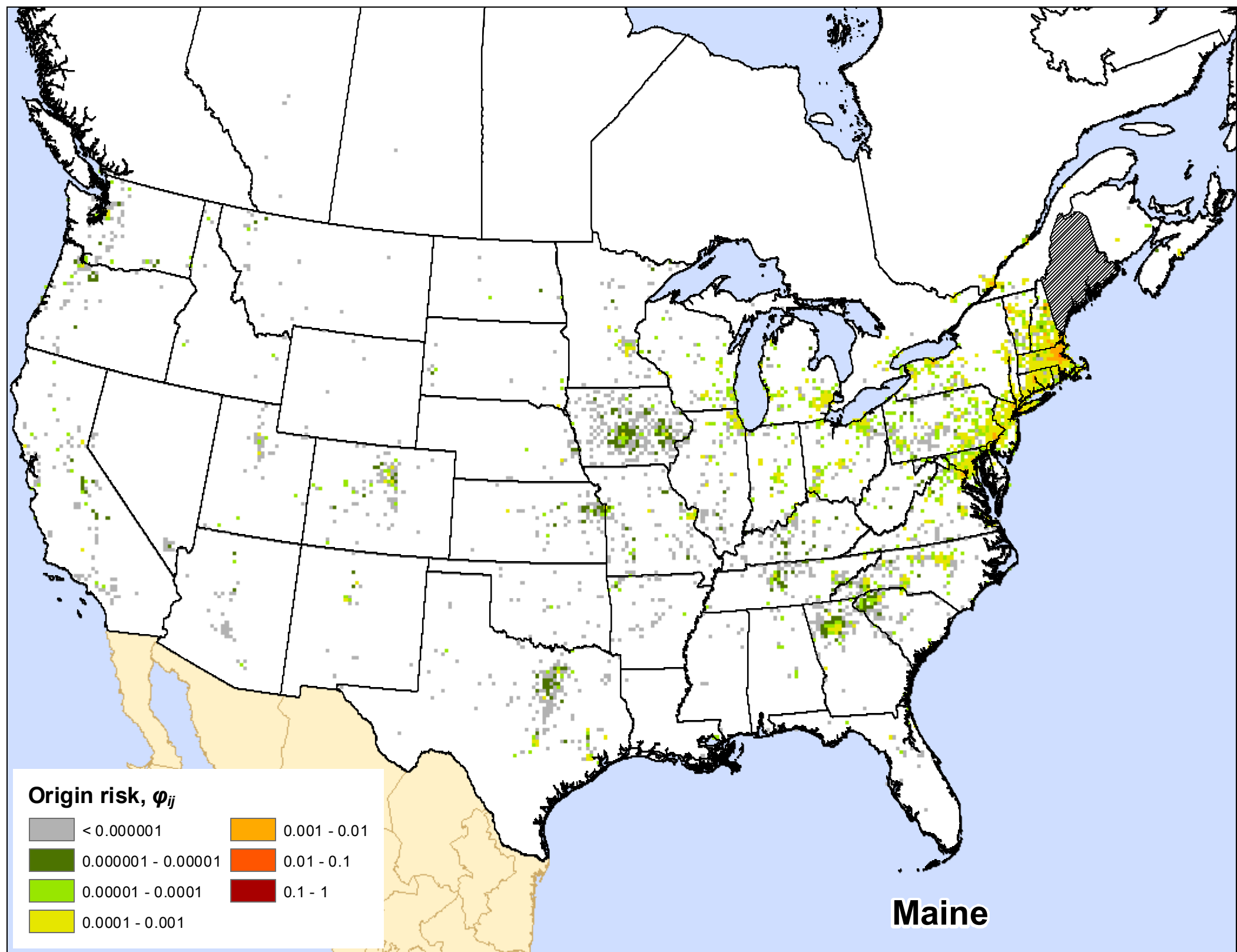

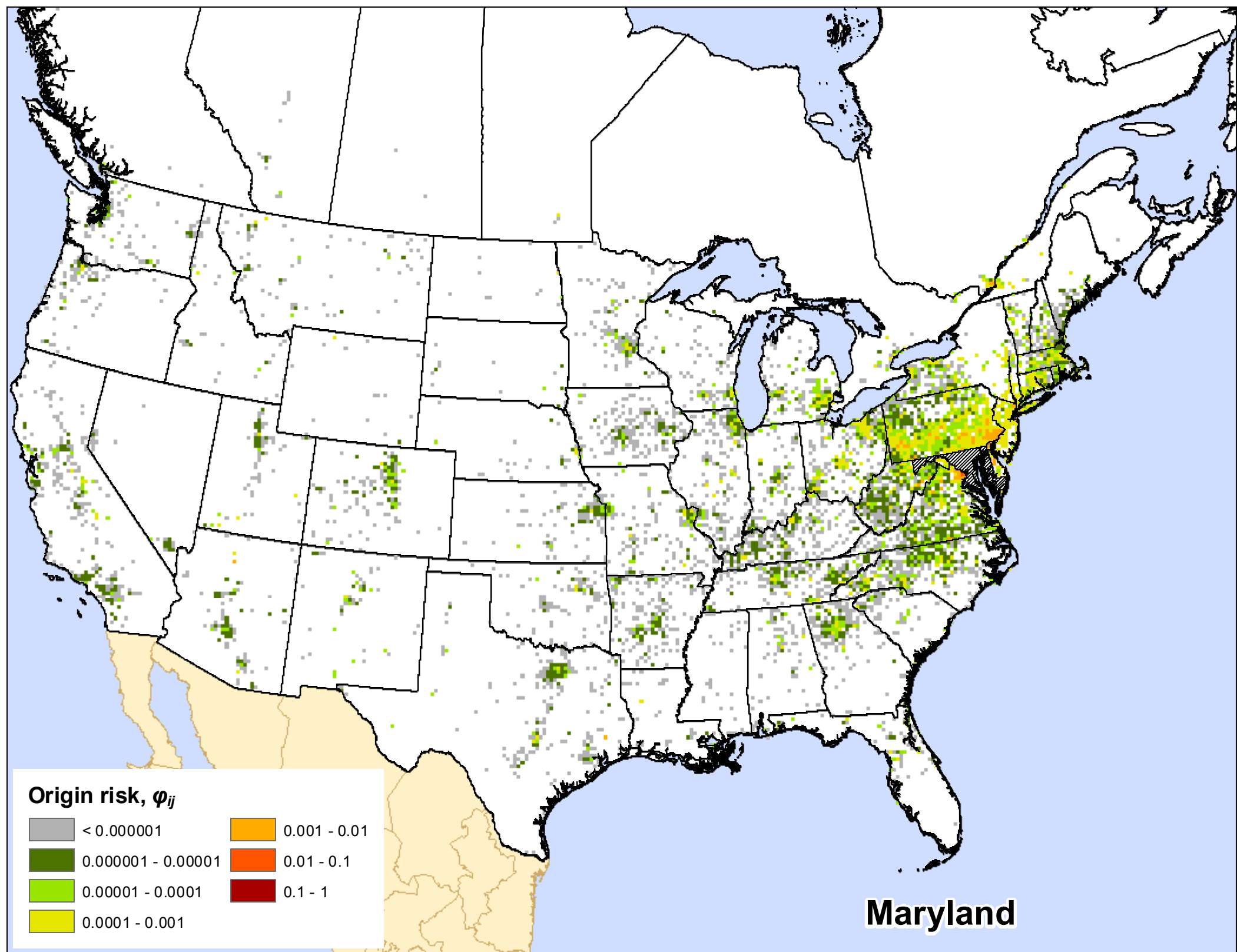

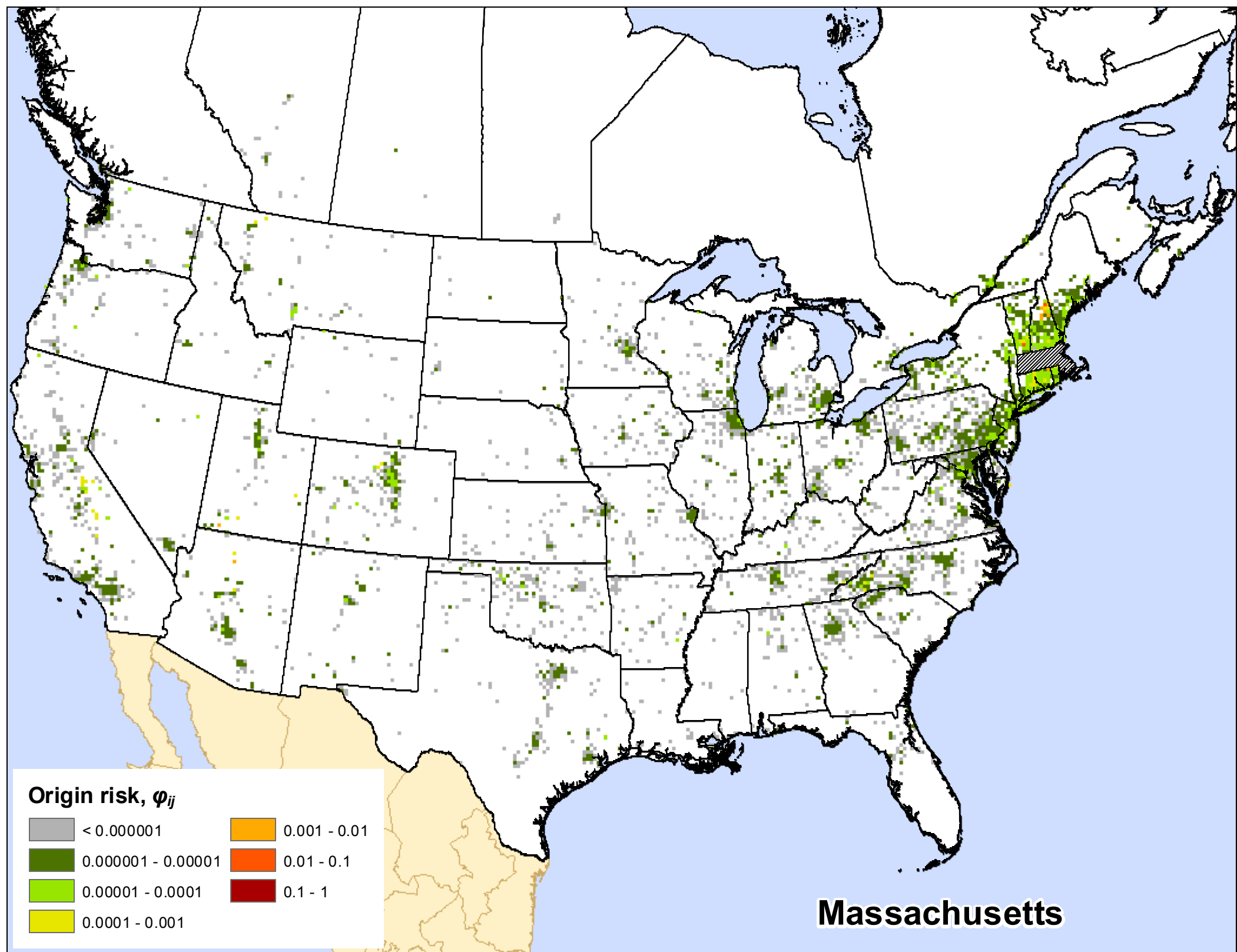

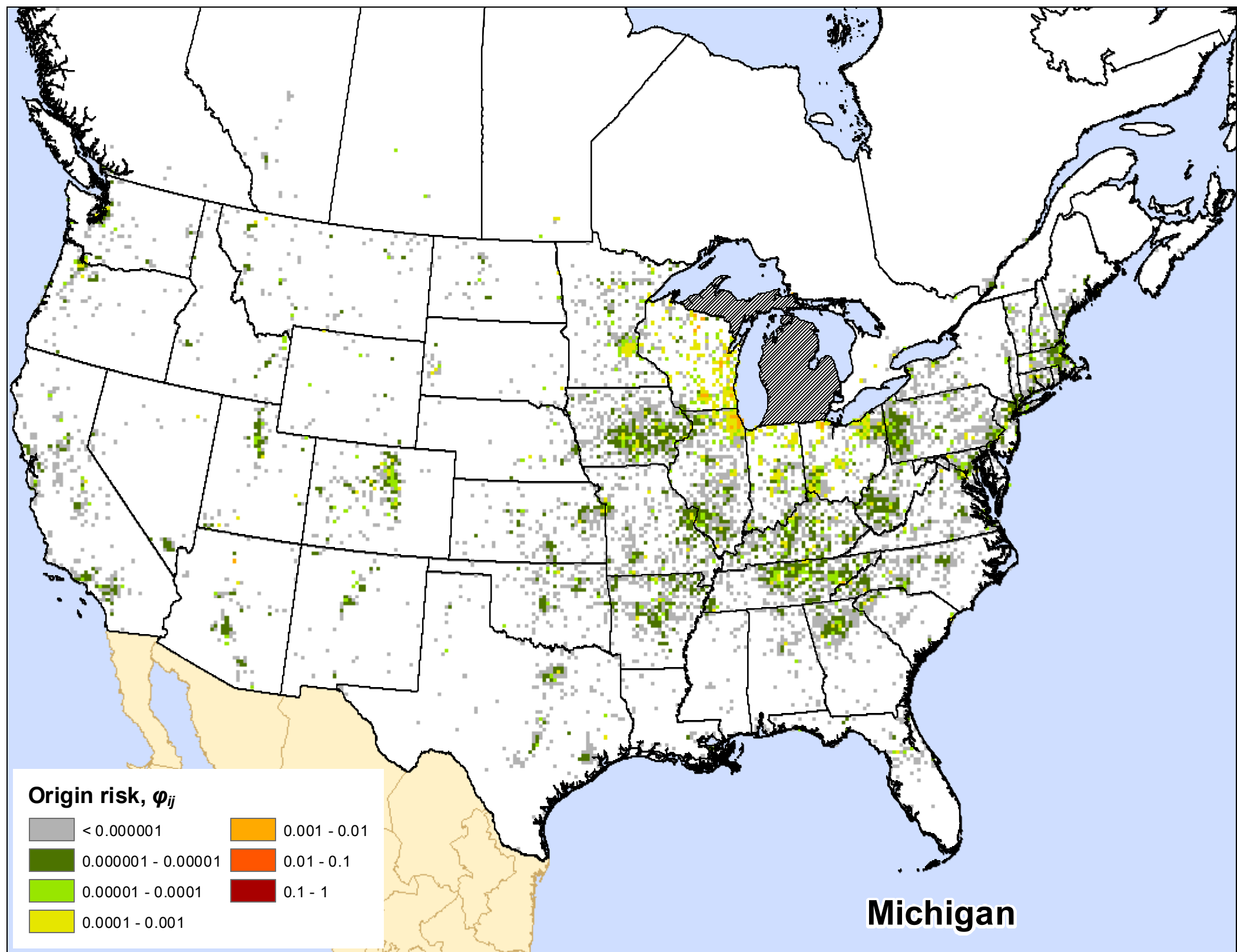

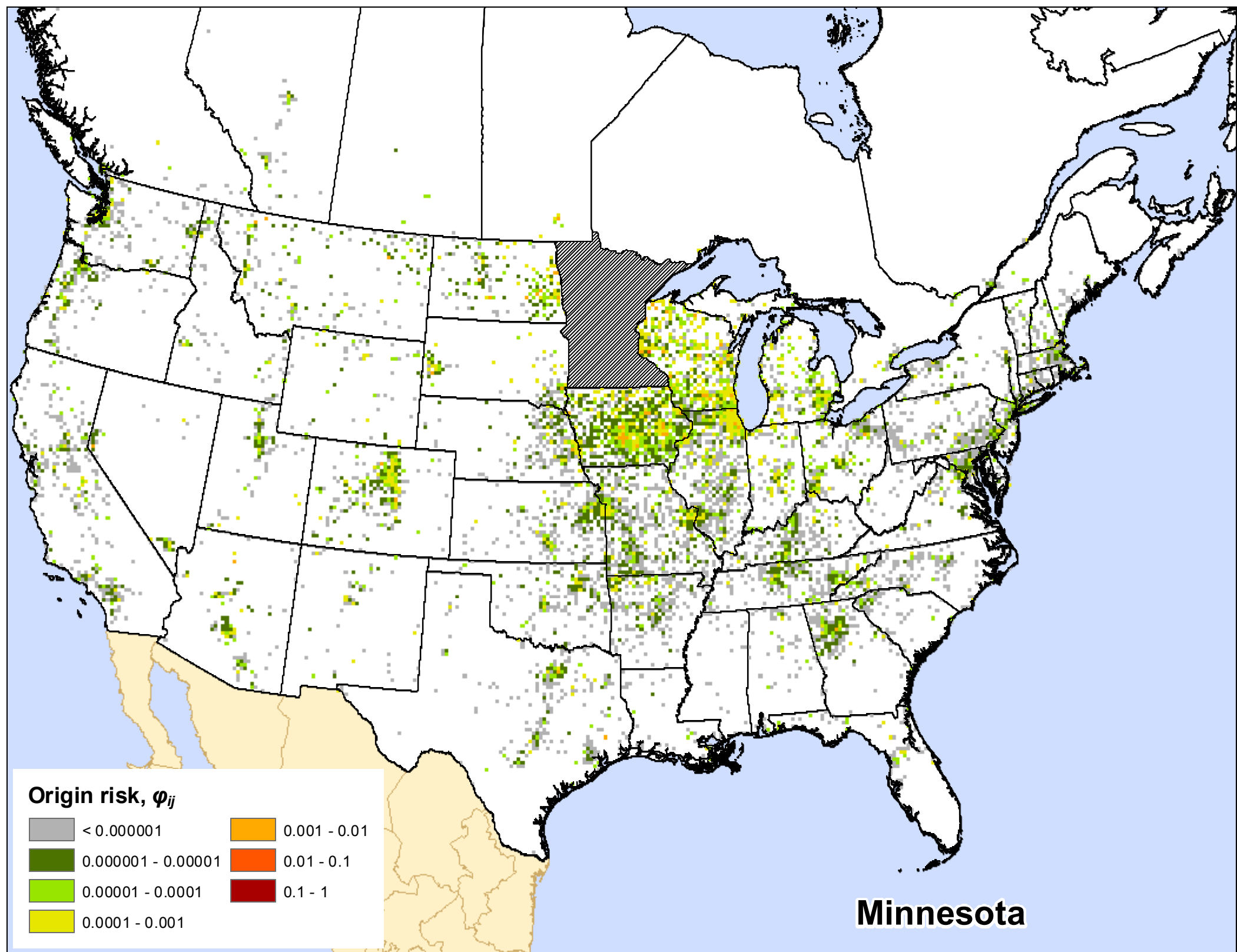

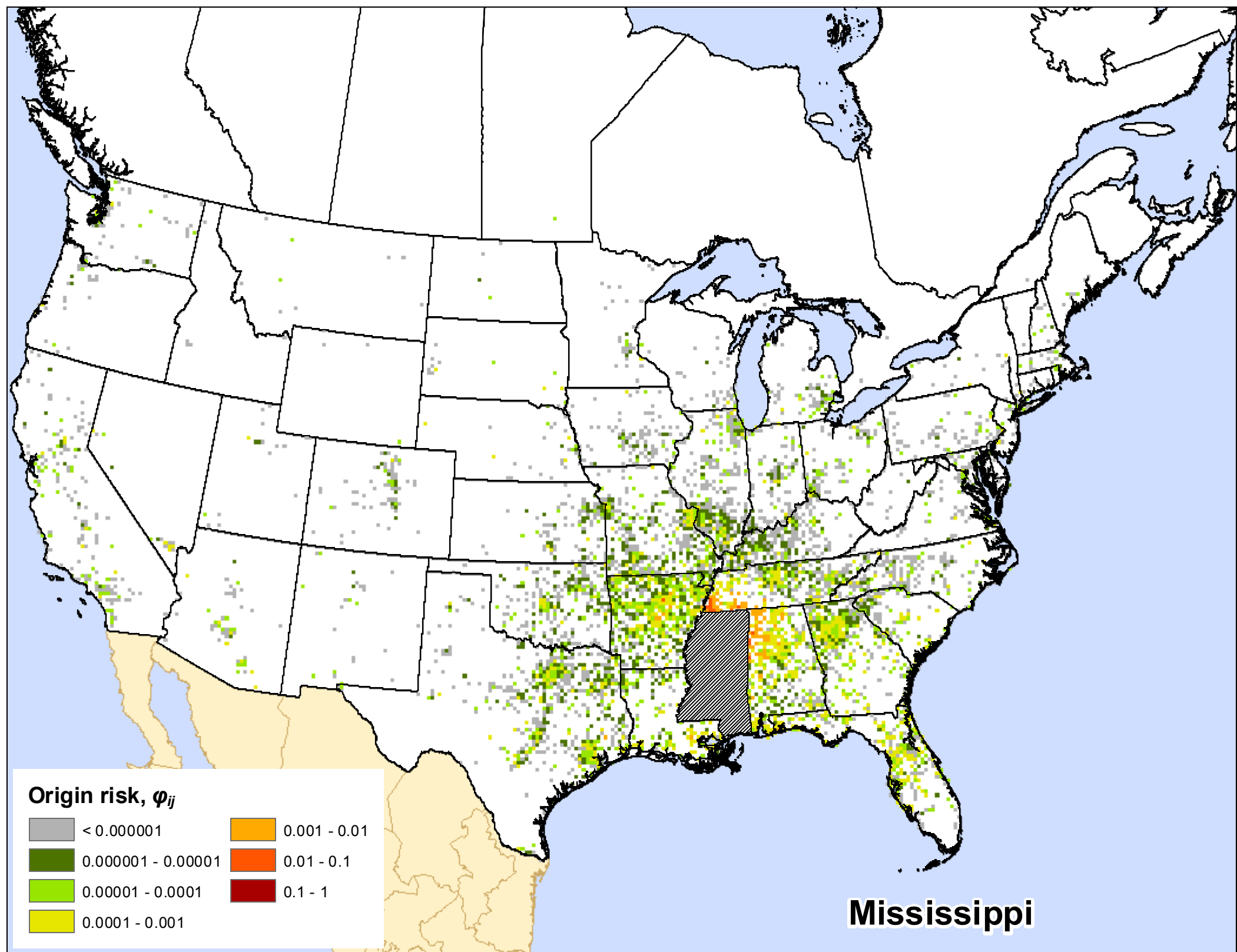

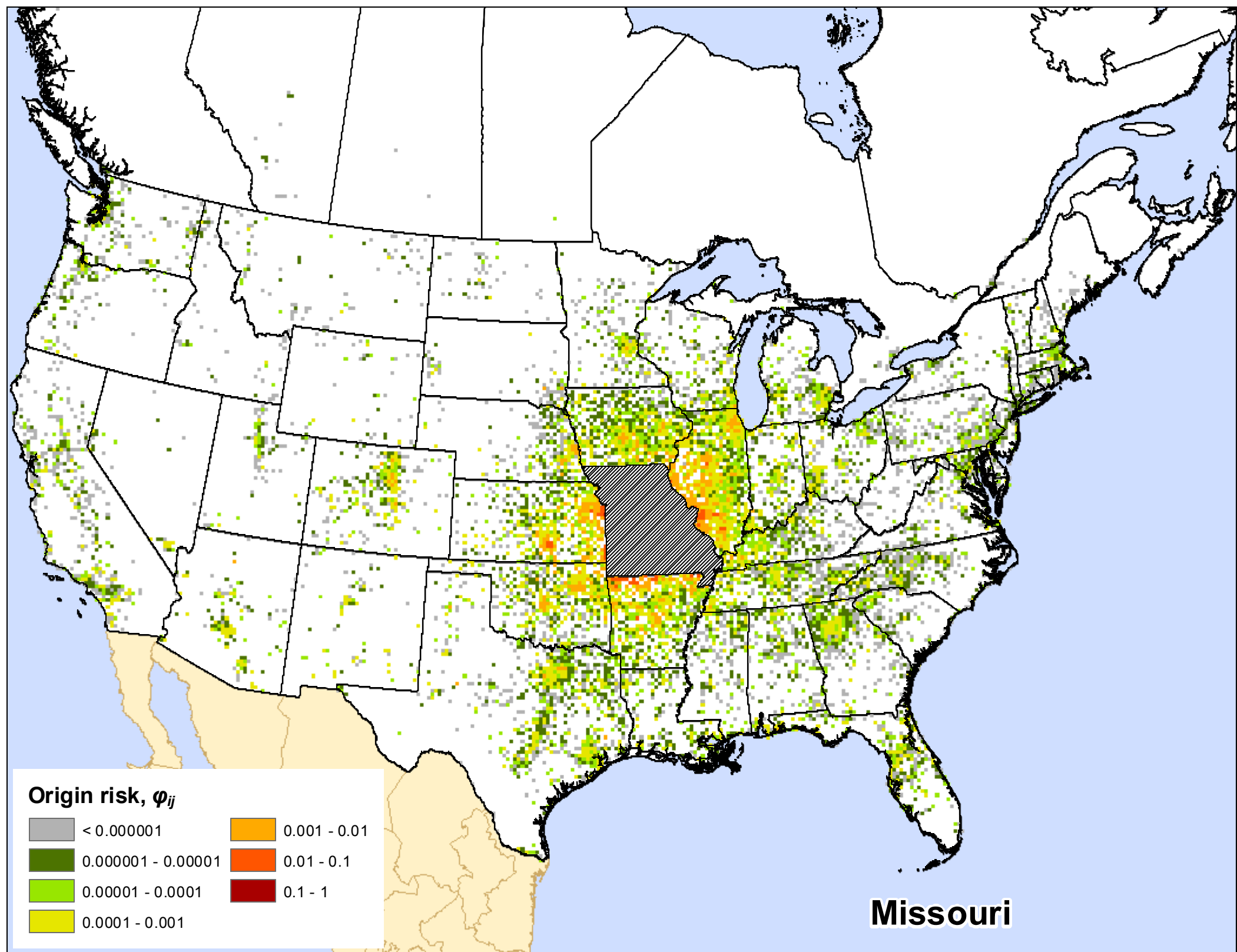

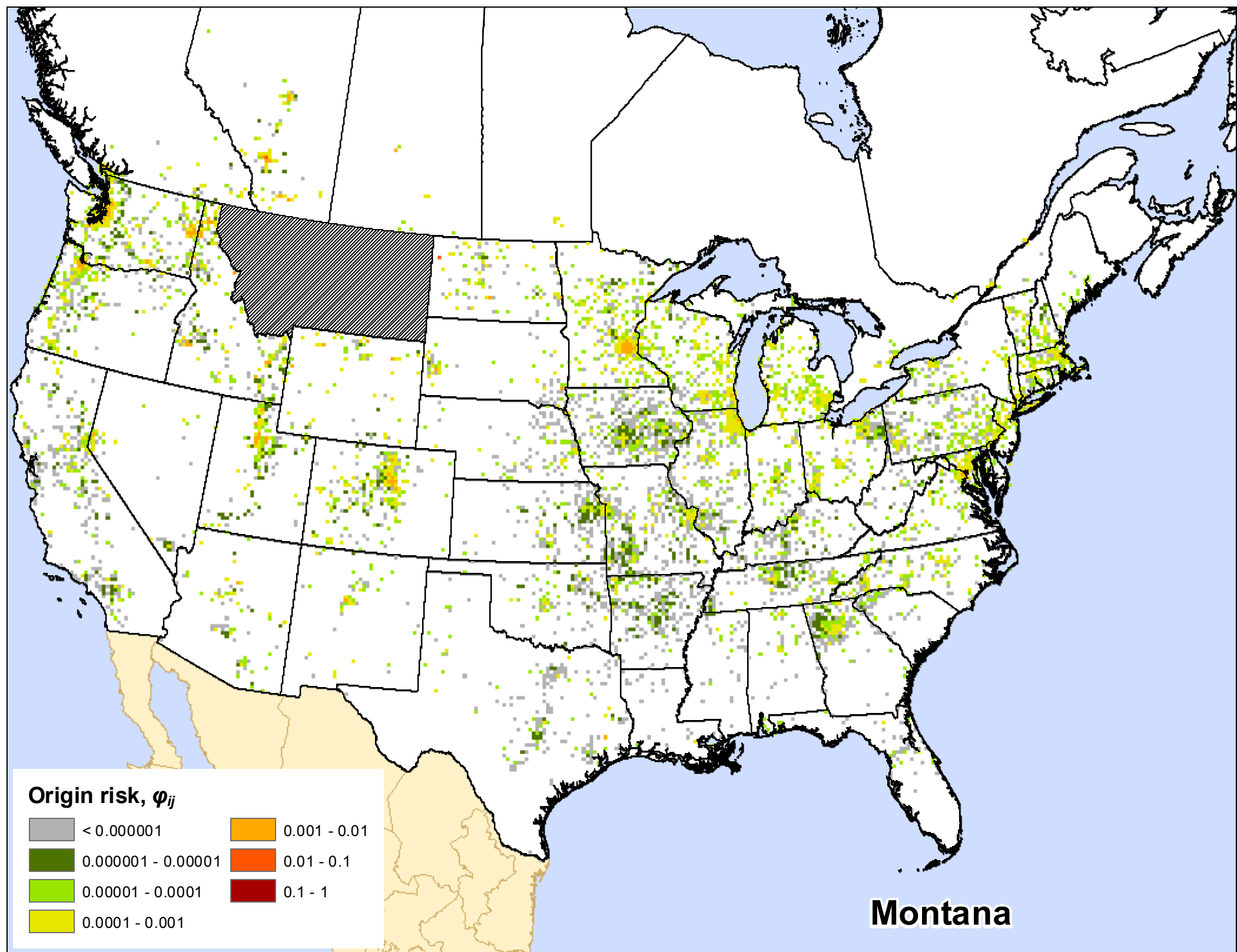

Supplement: Appendix S3 — Out-of-state origin risk maps for 24 US states: Alabama – Montana. (PDF) [file pone.0102105.s003.pdf]

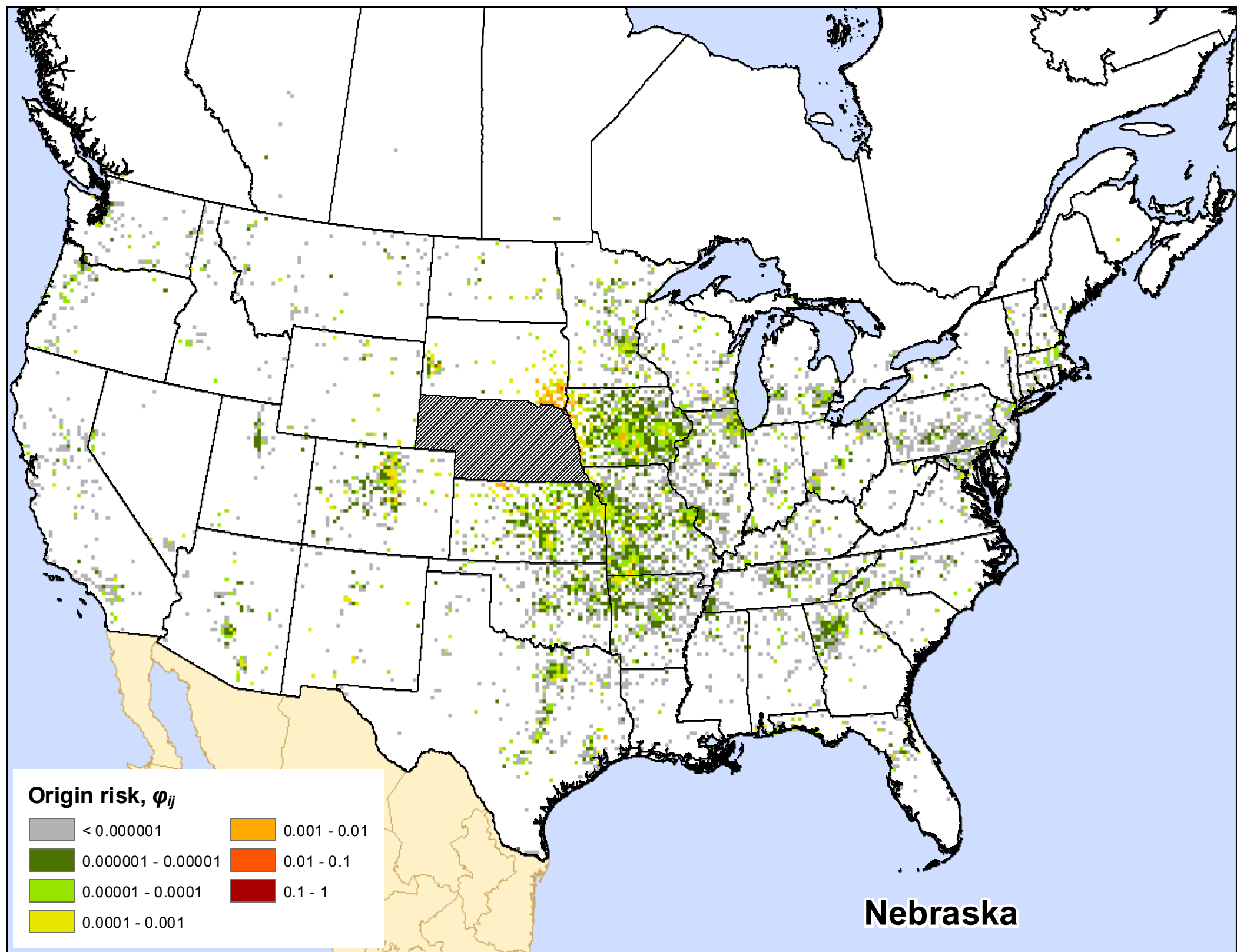

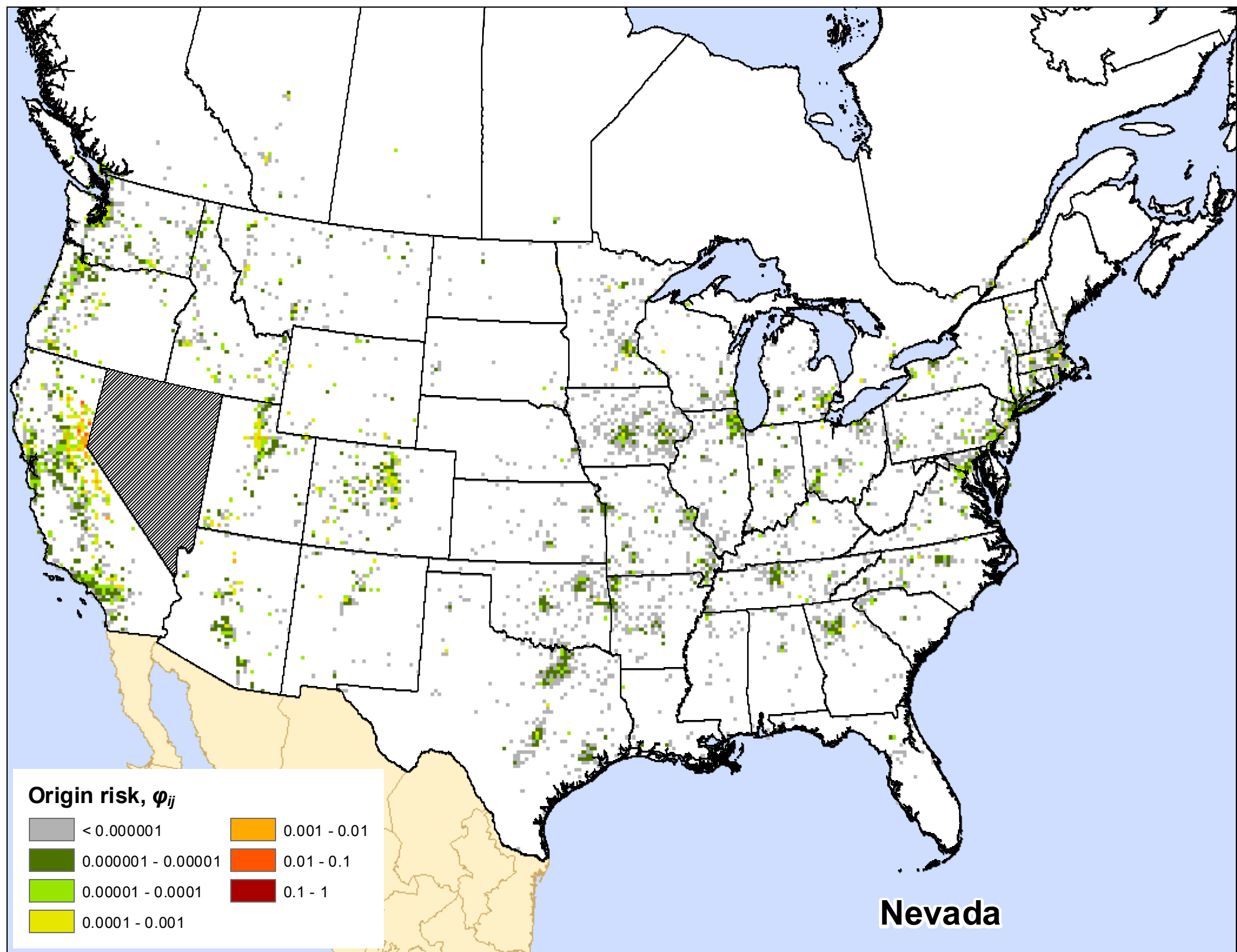

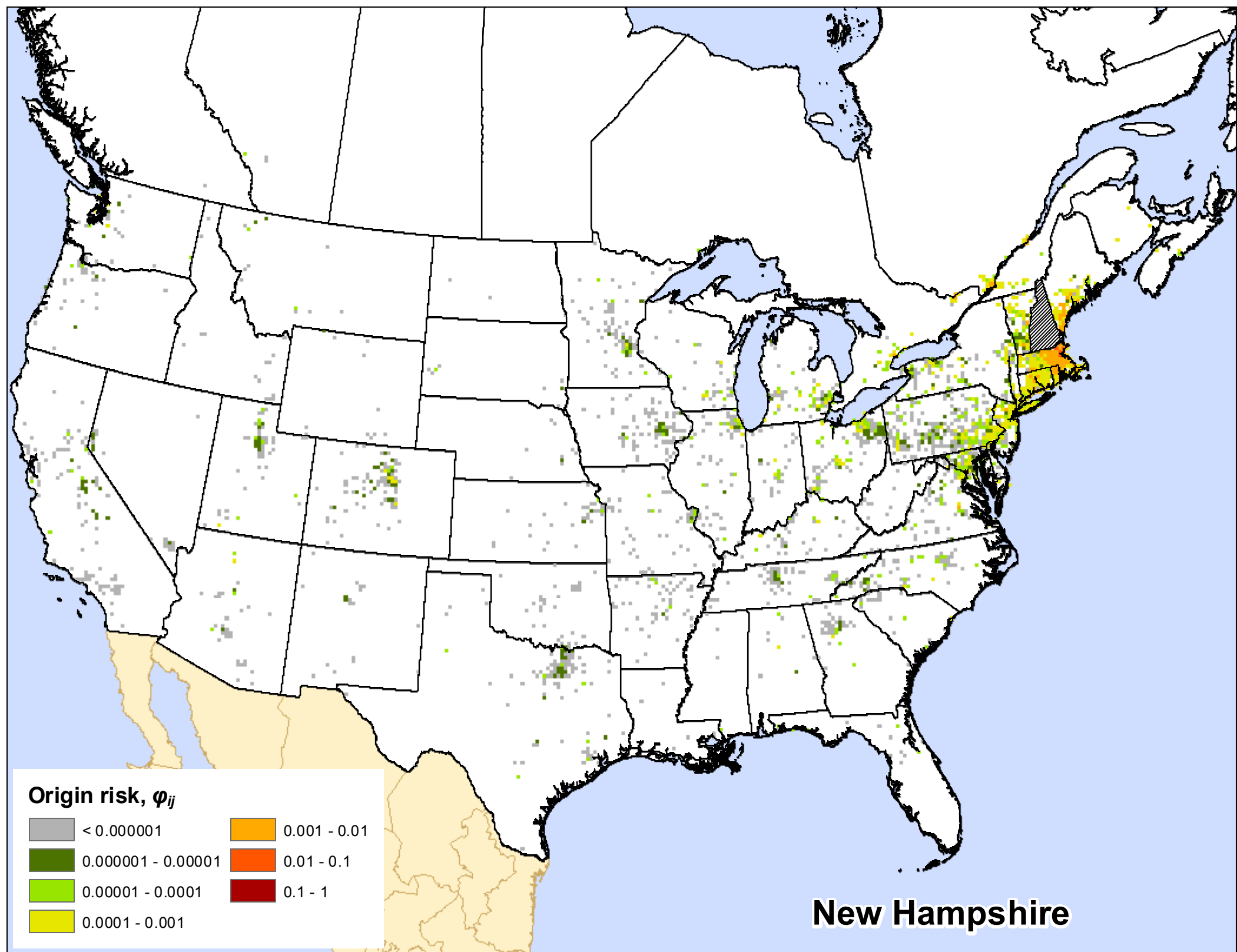

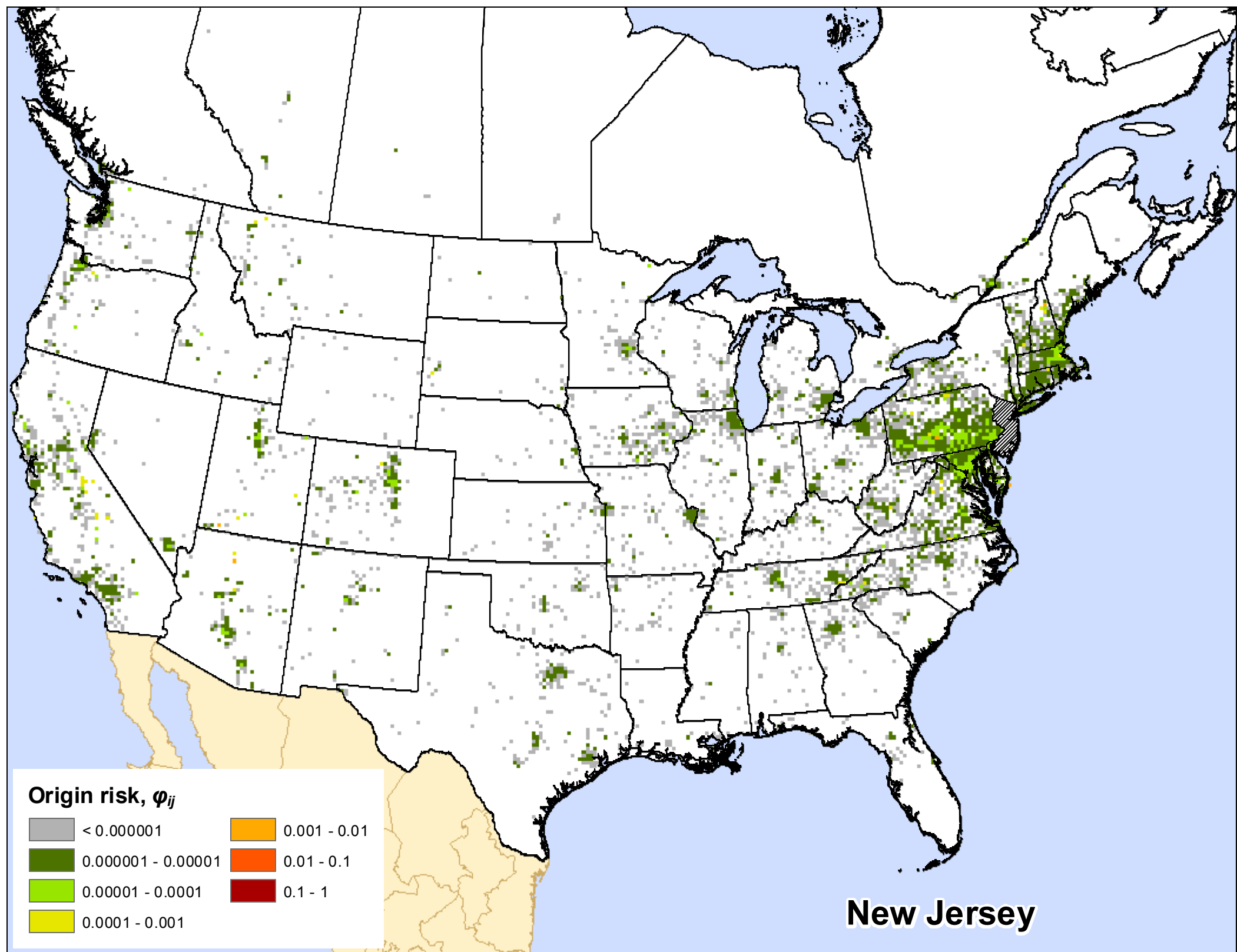

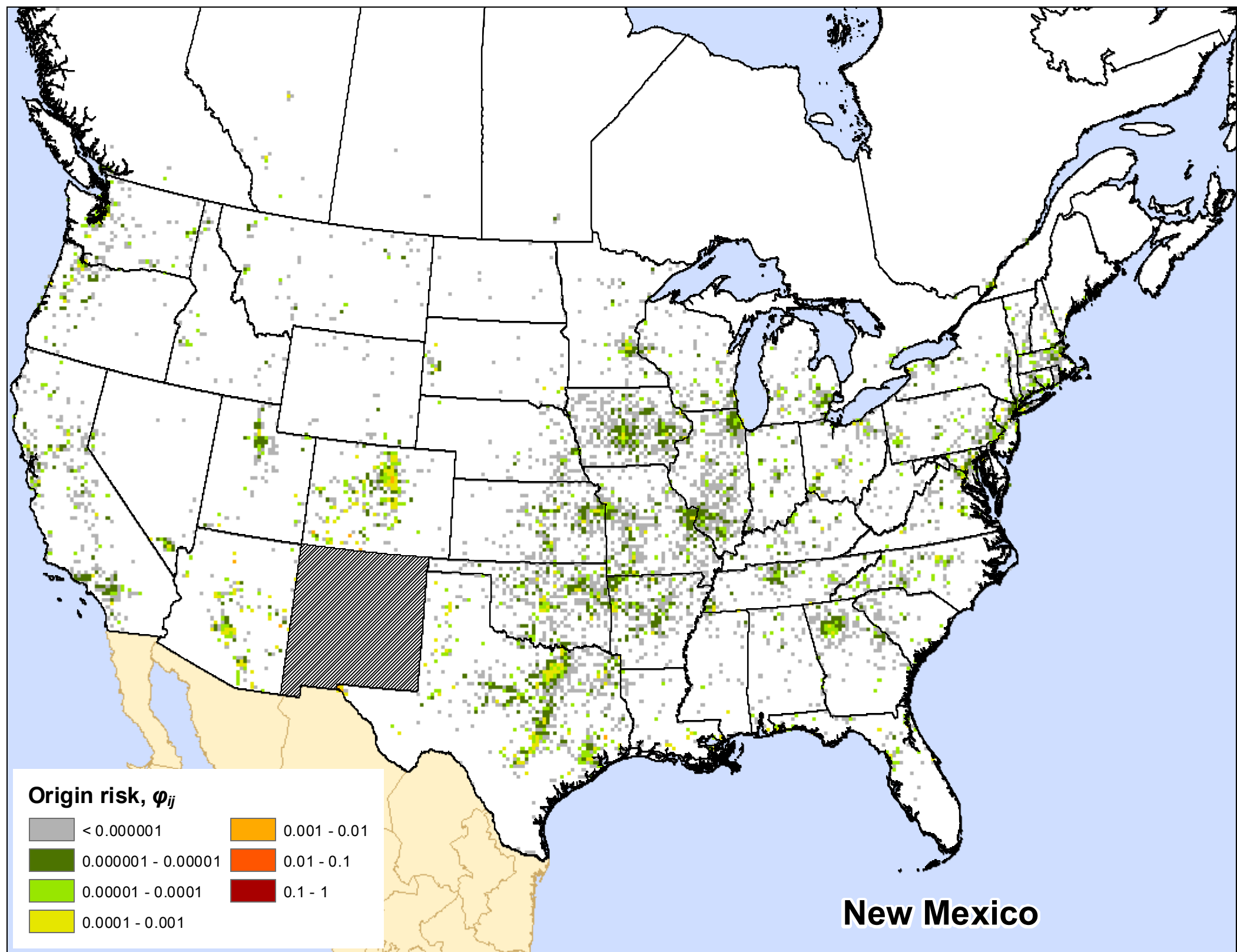

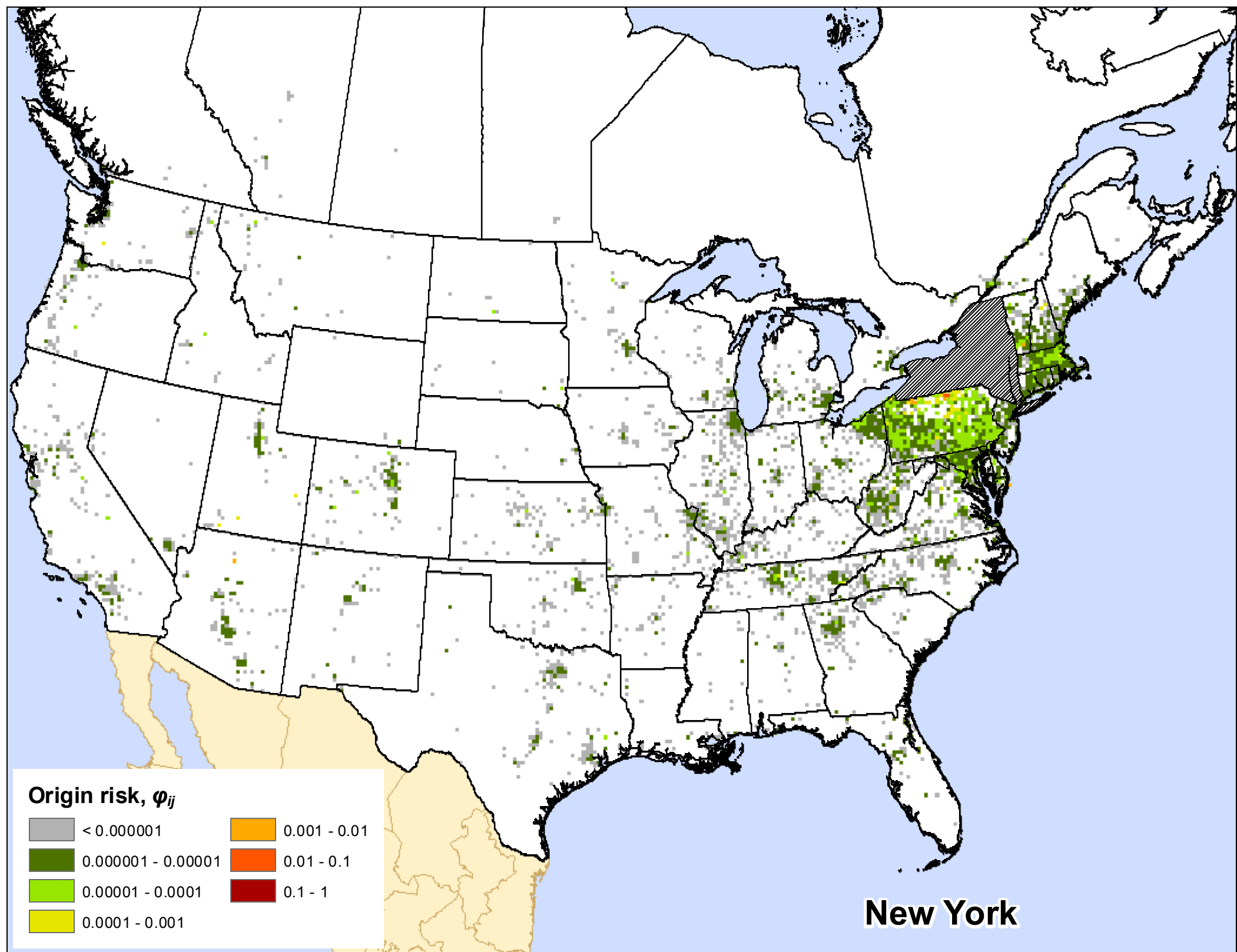

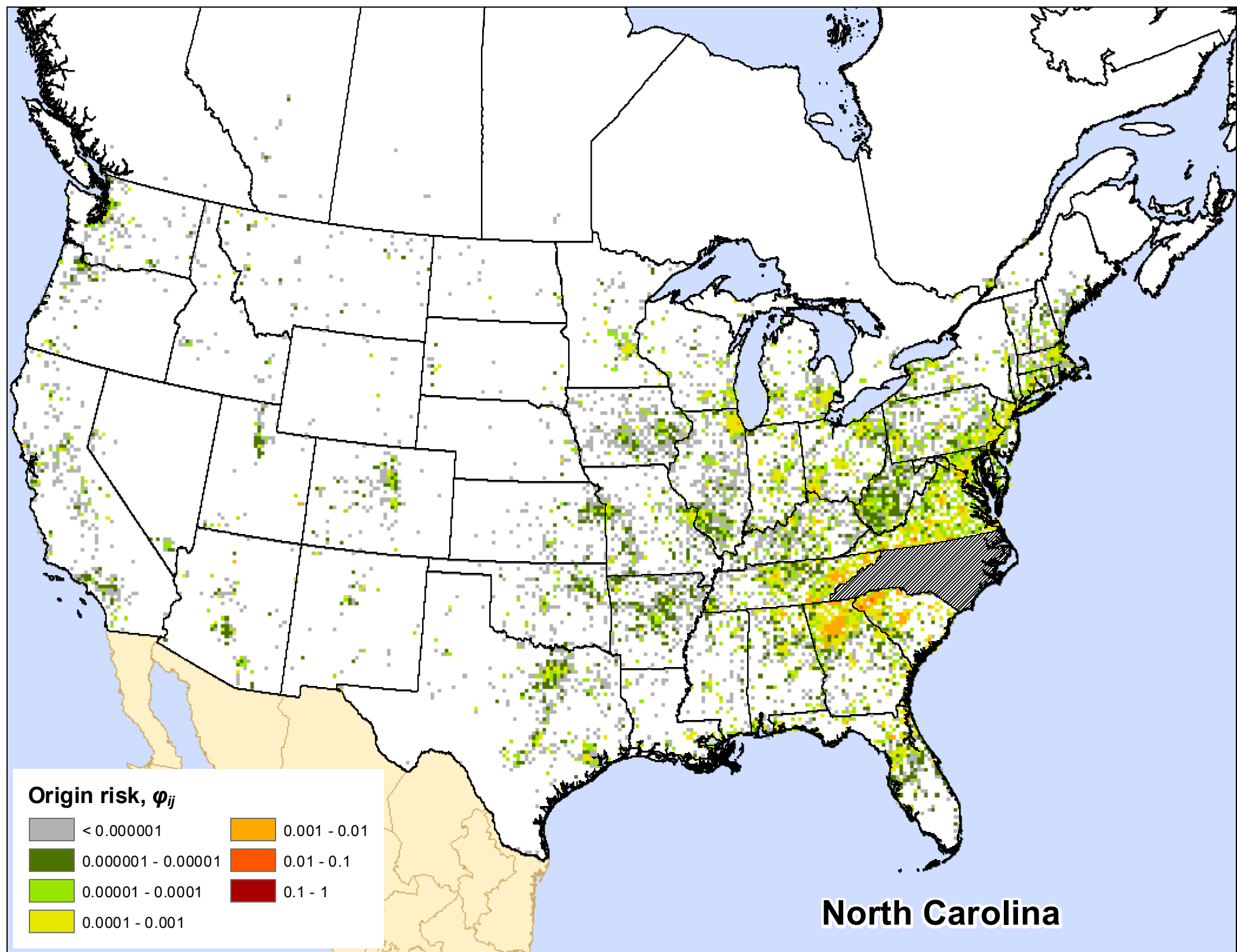

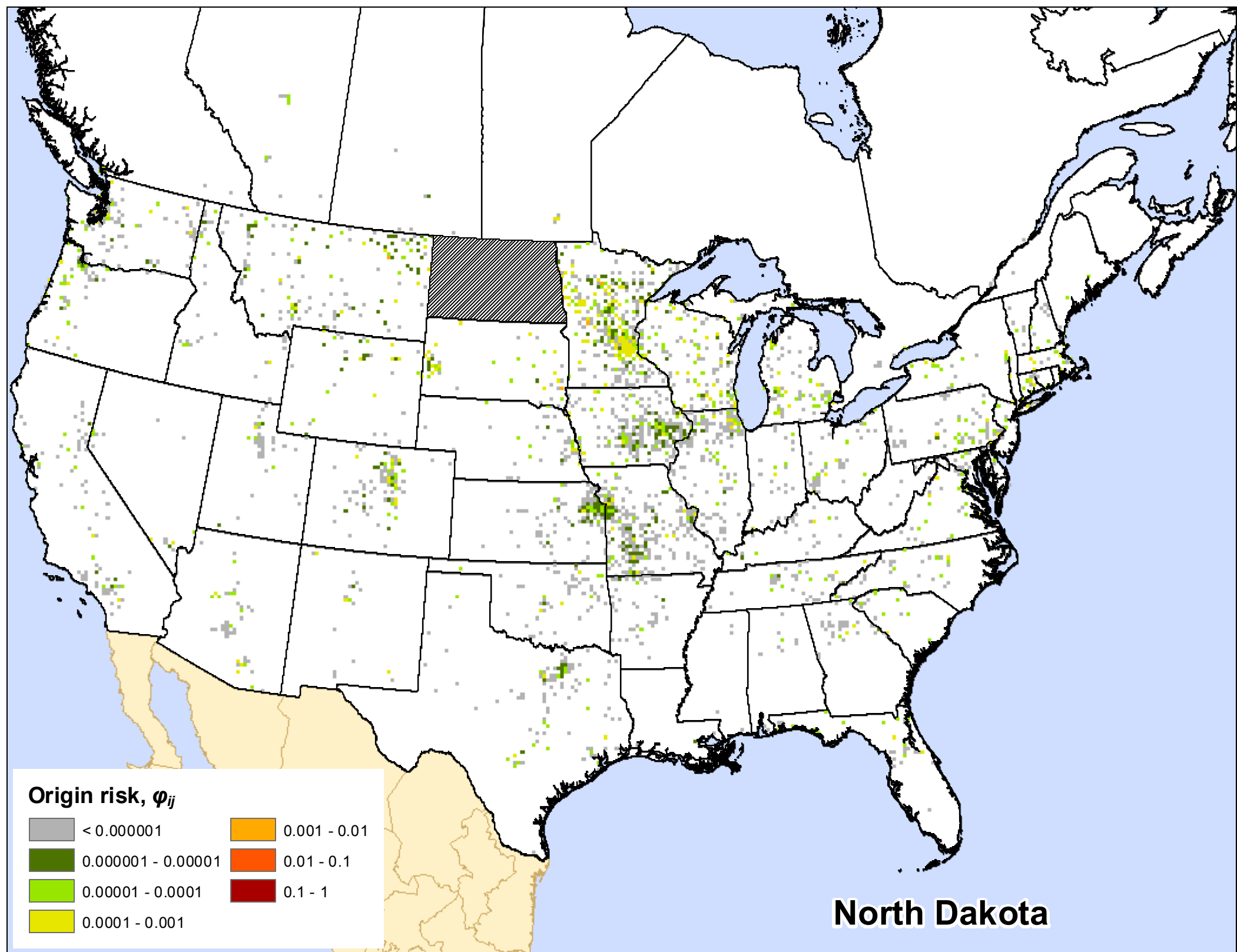

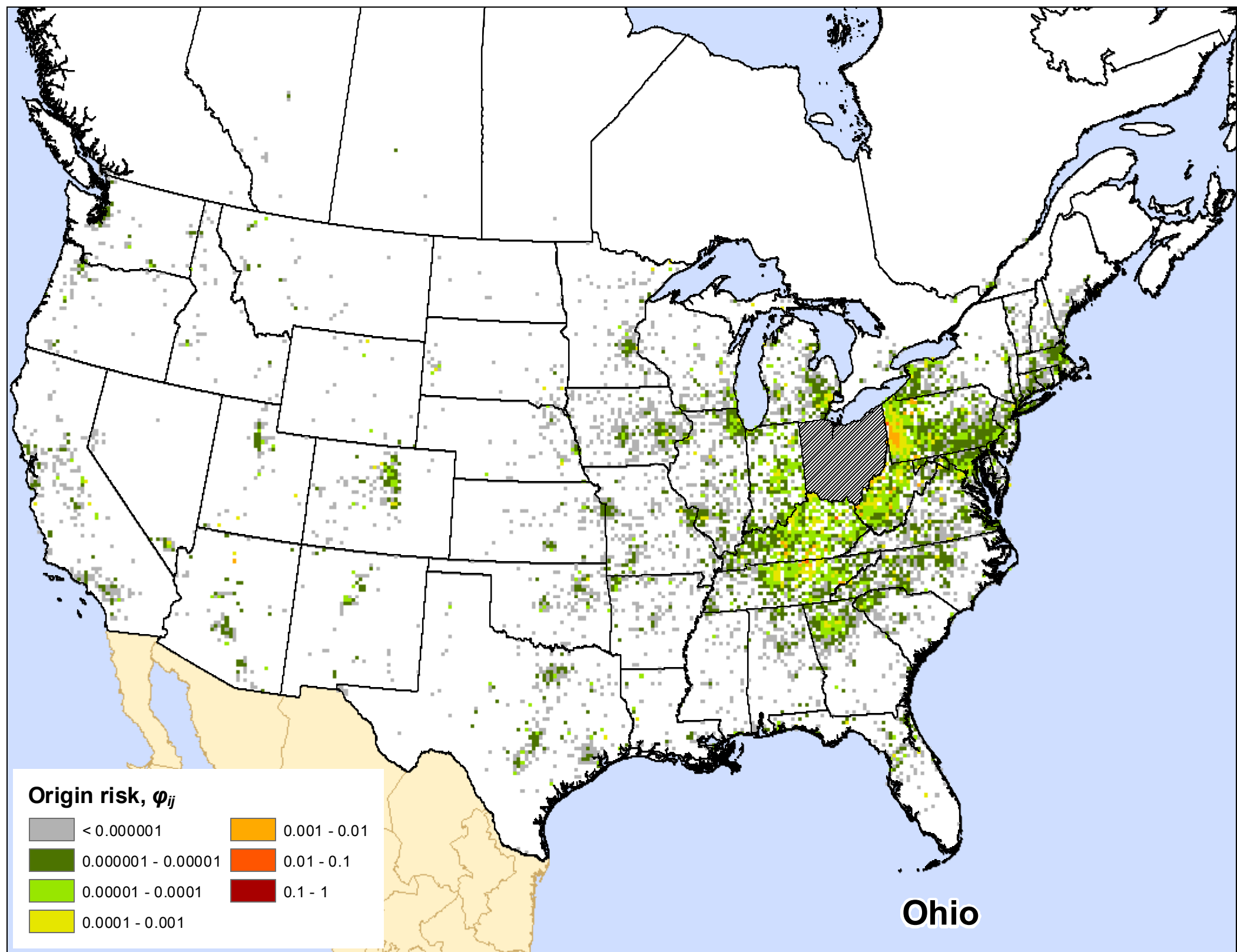

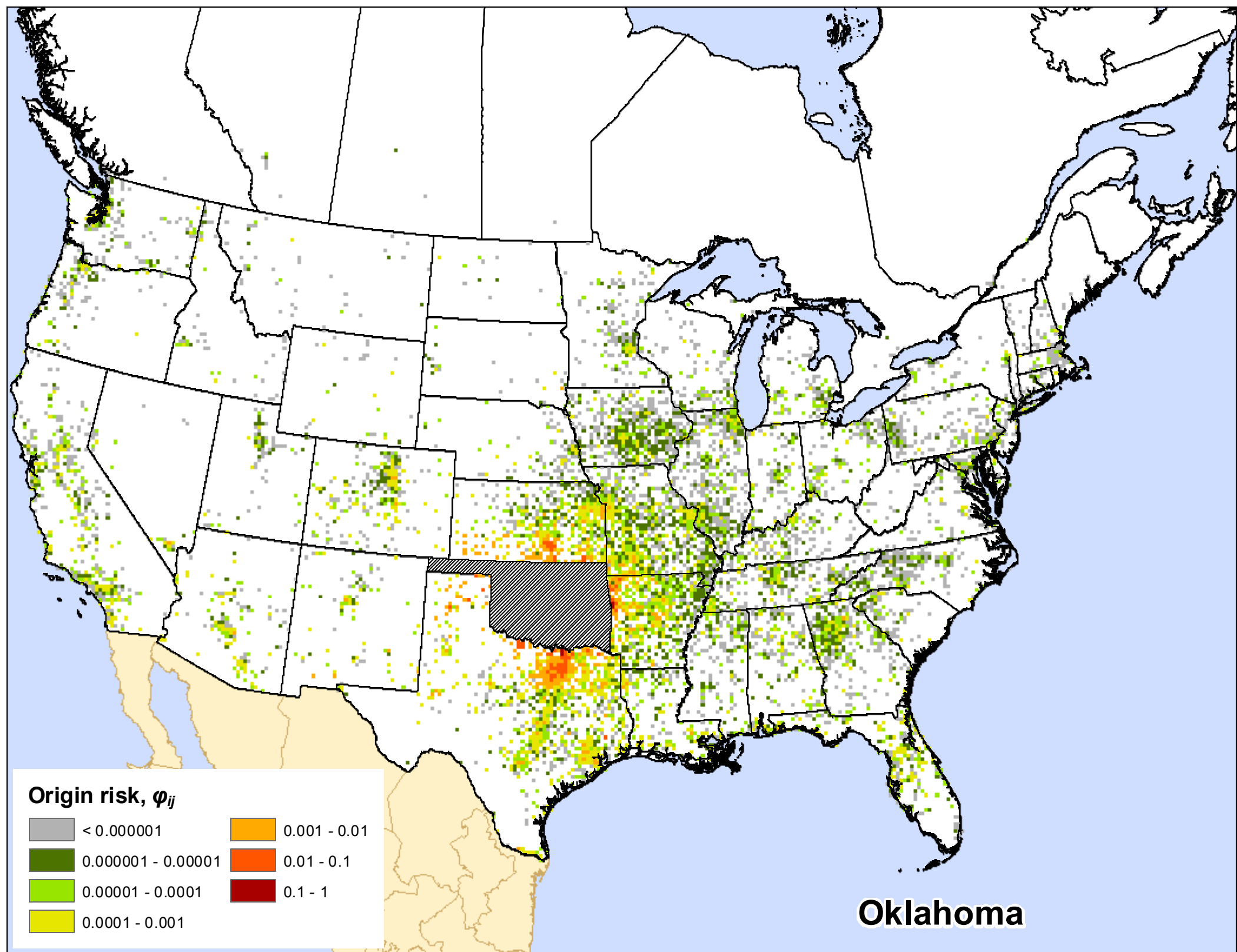

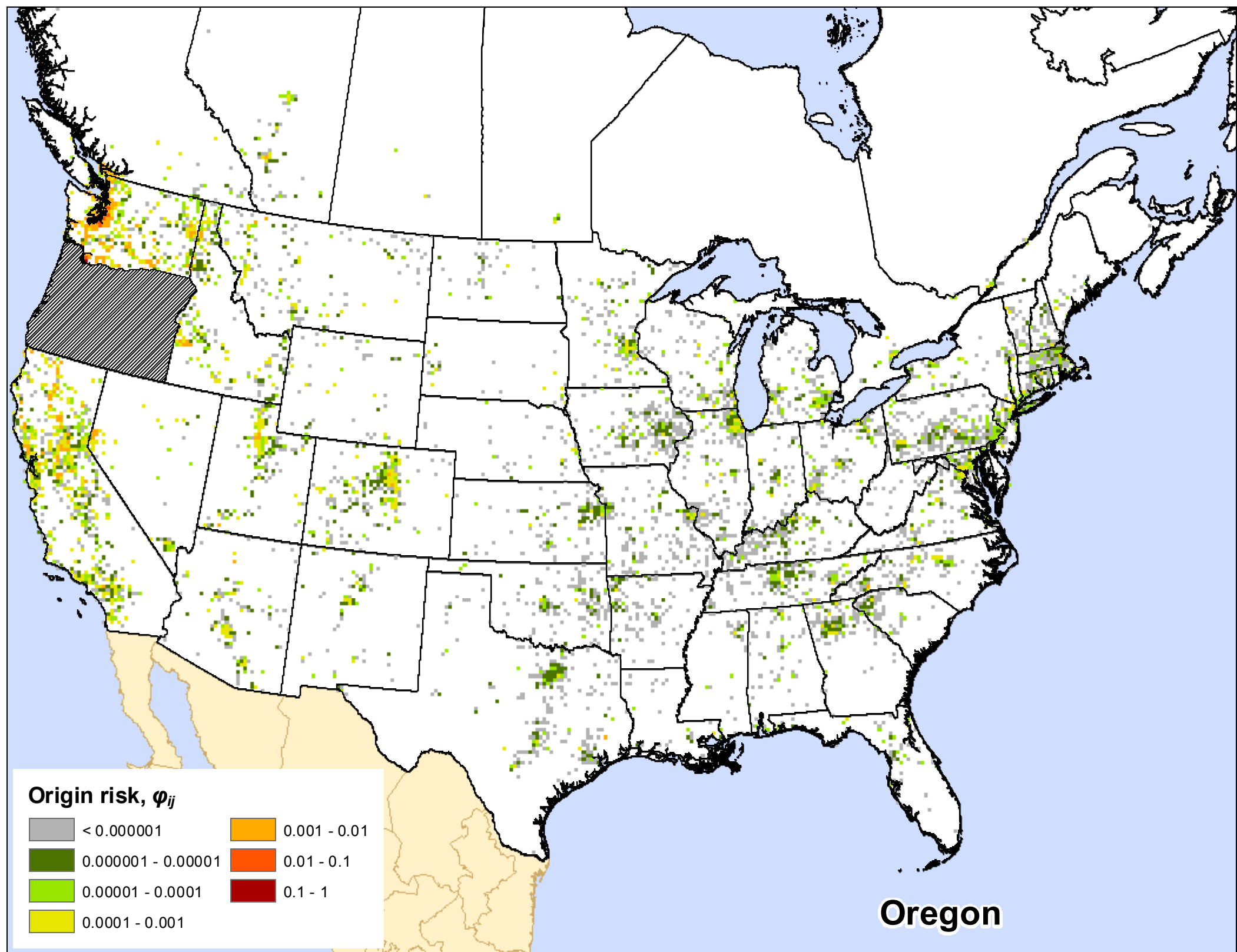

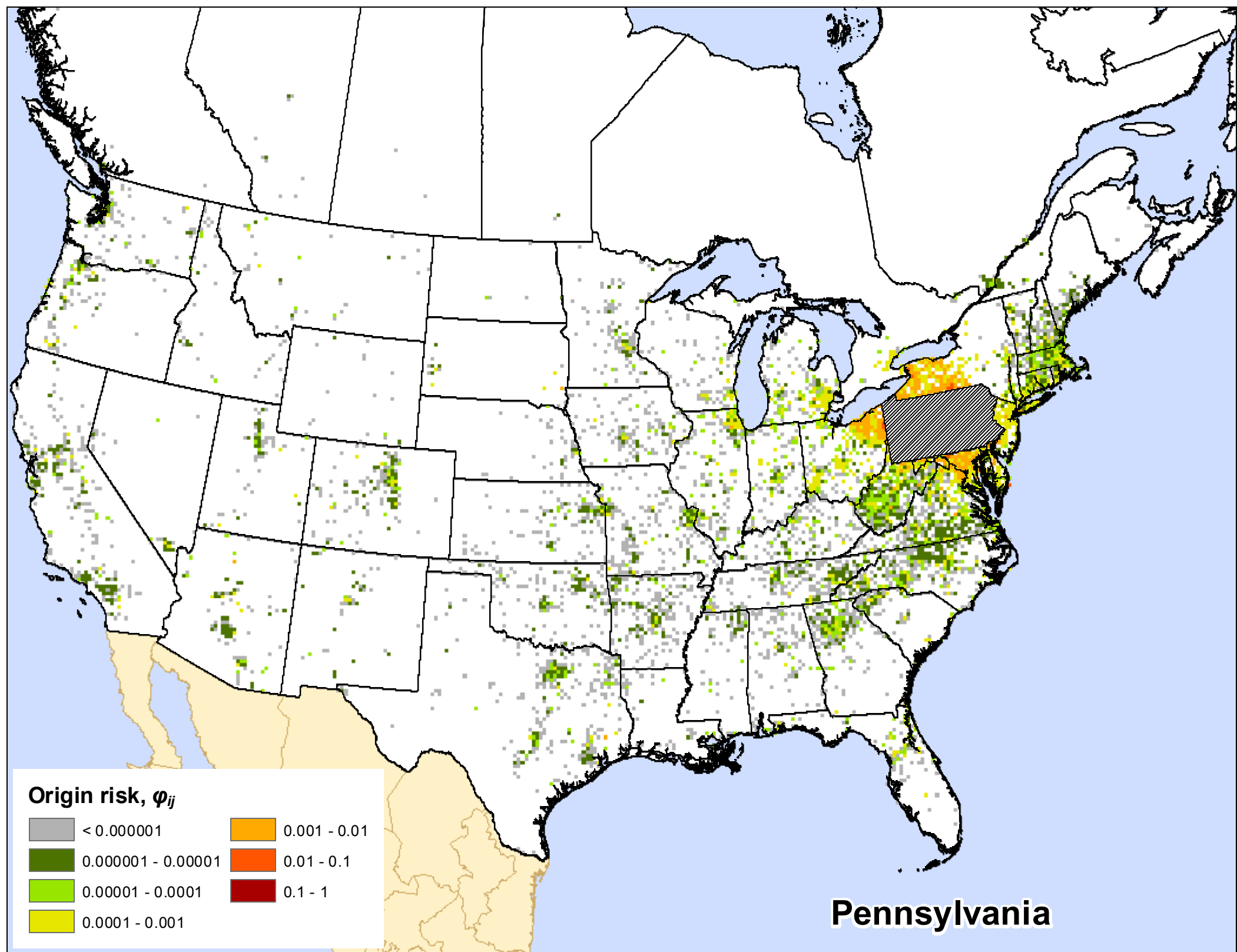

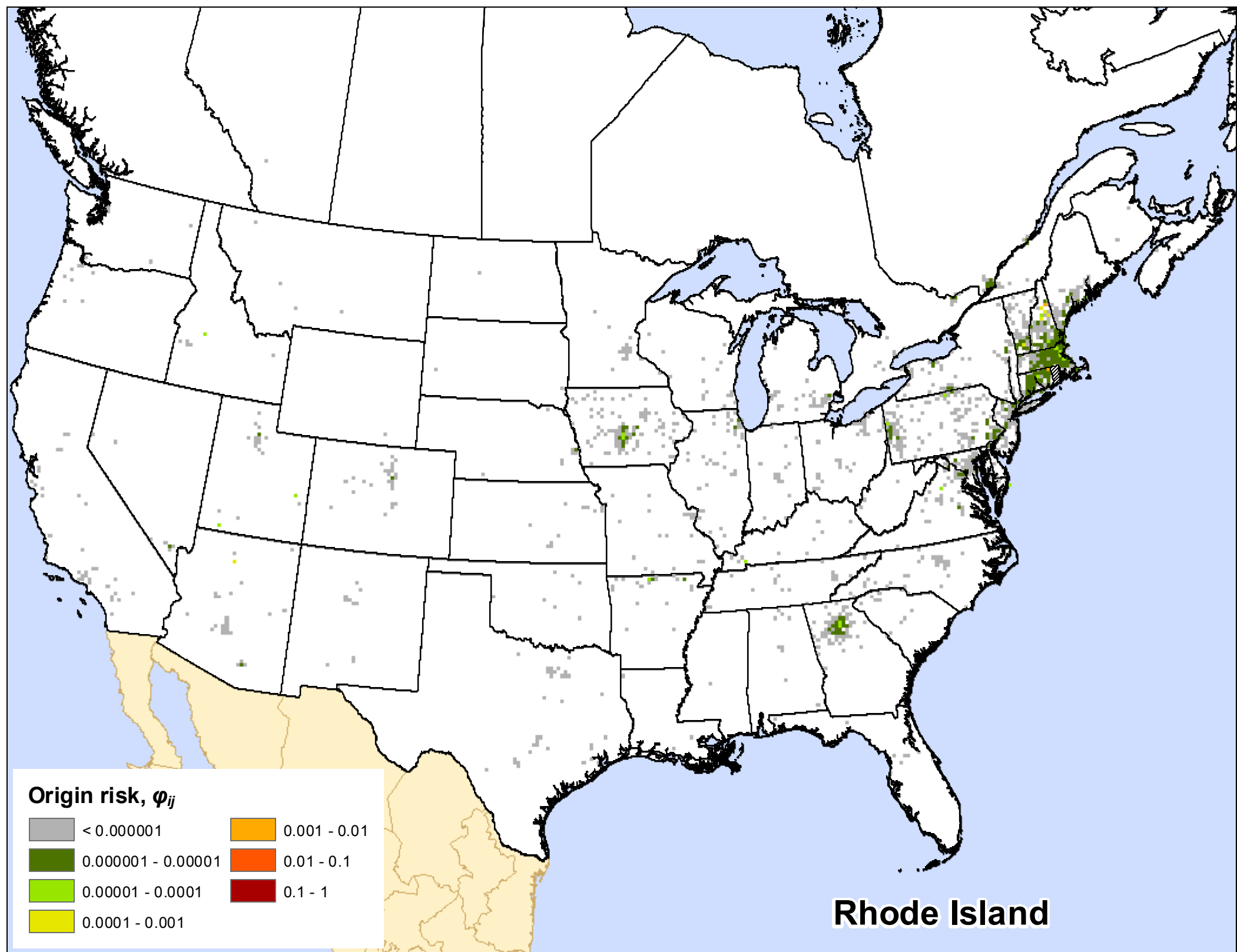

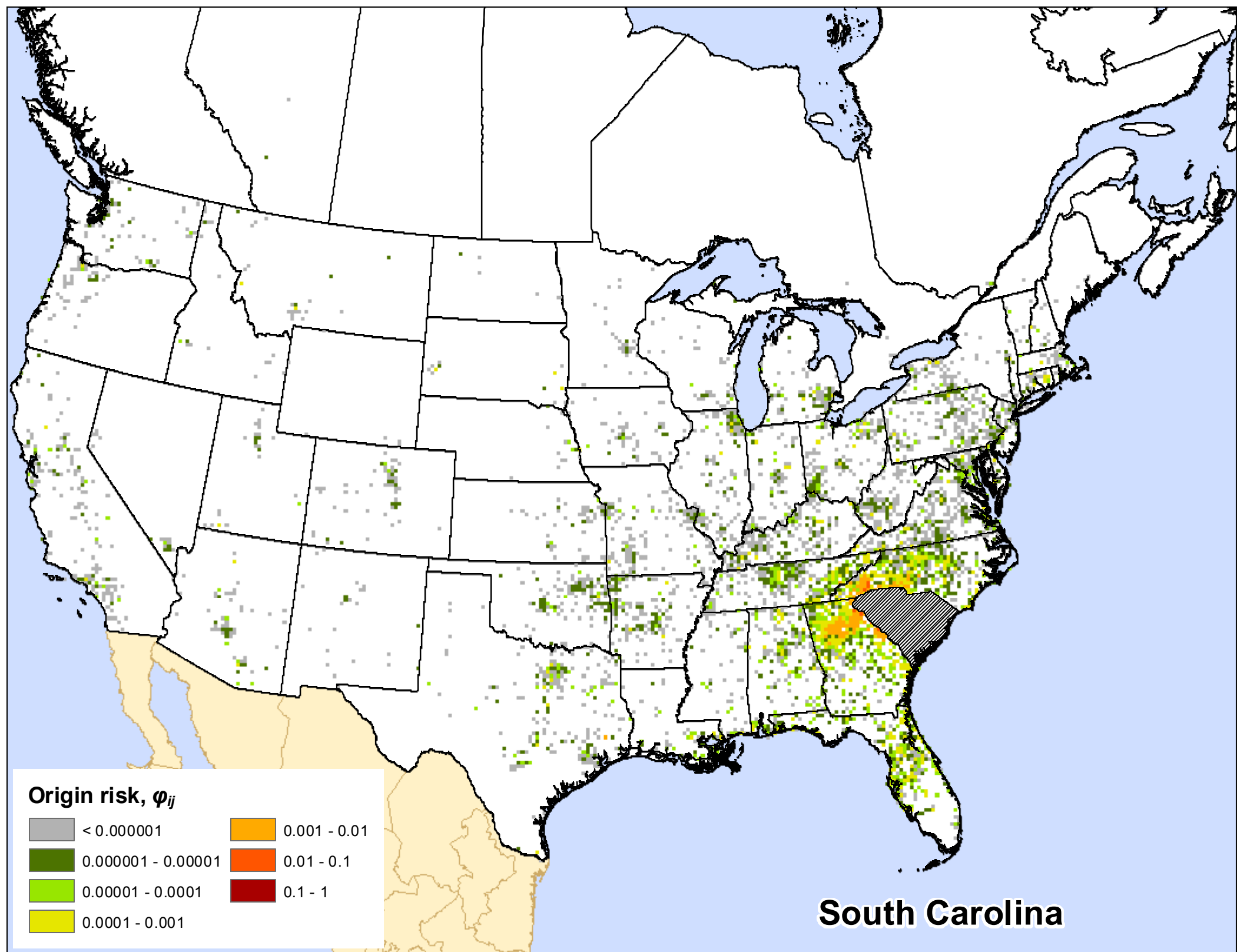

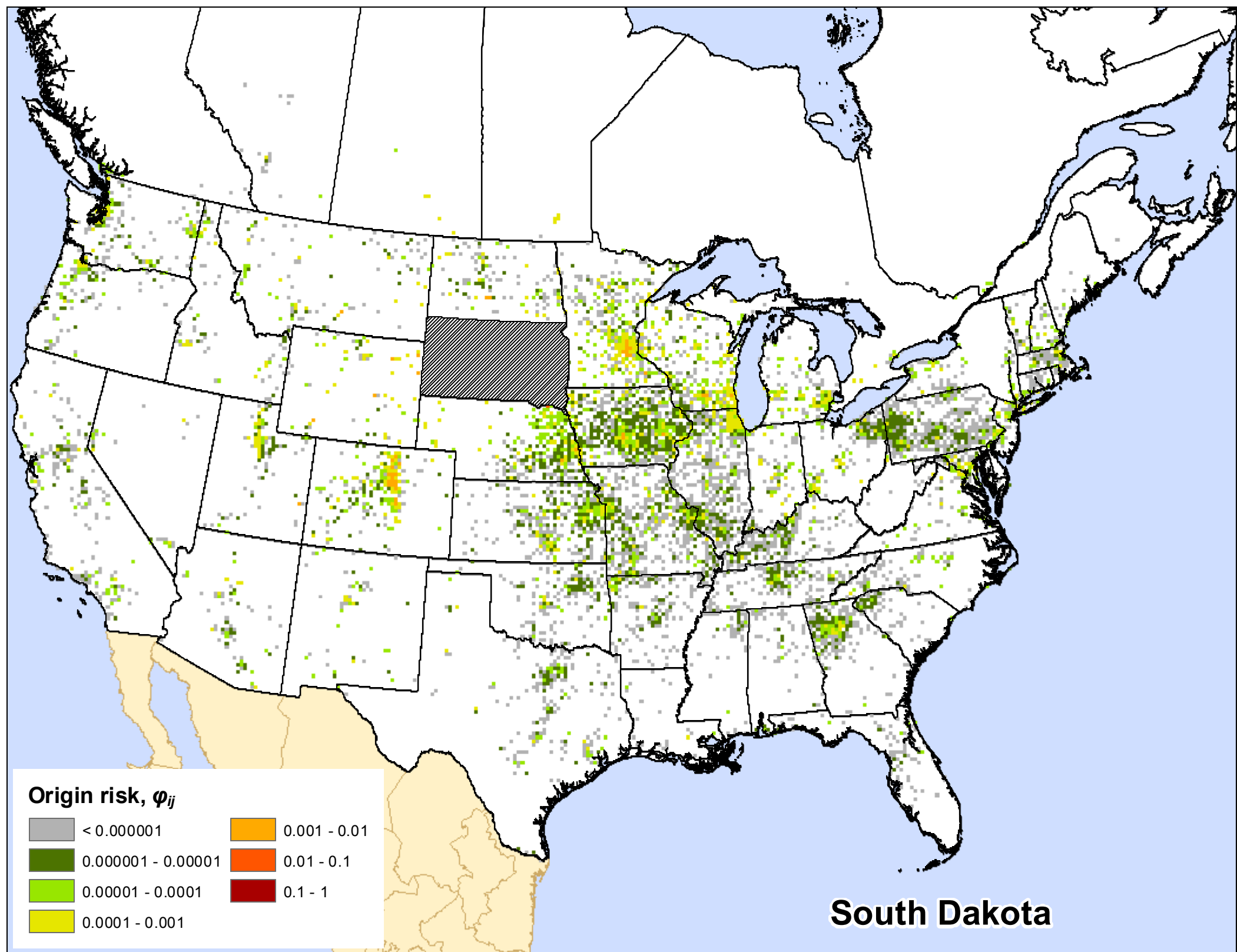

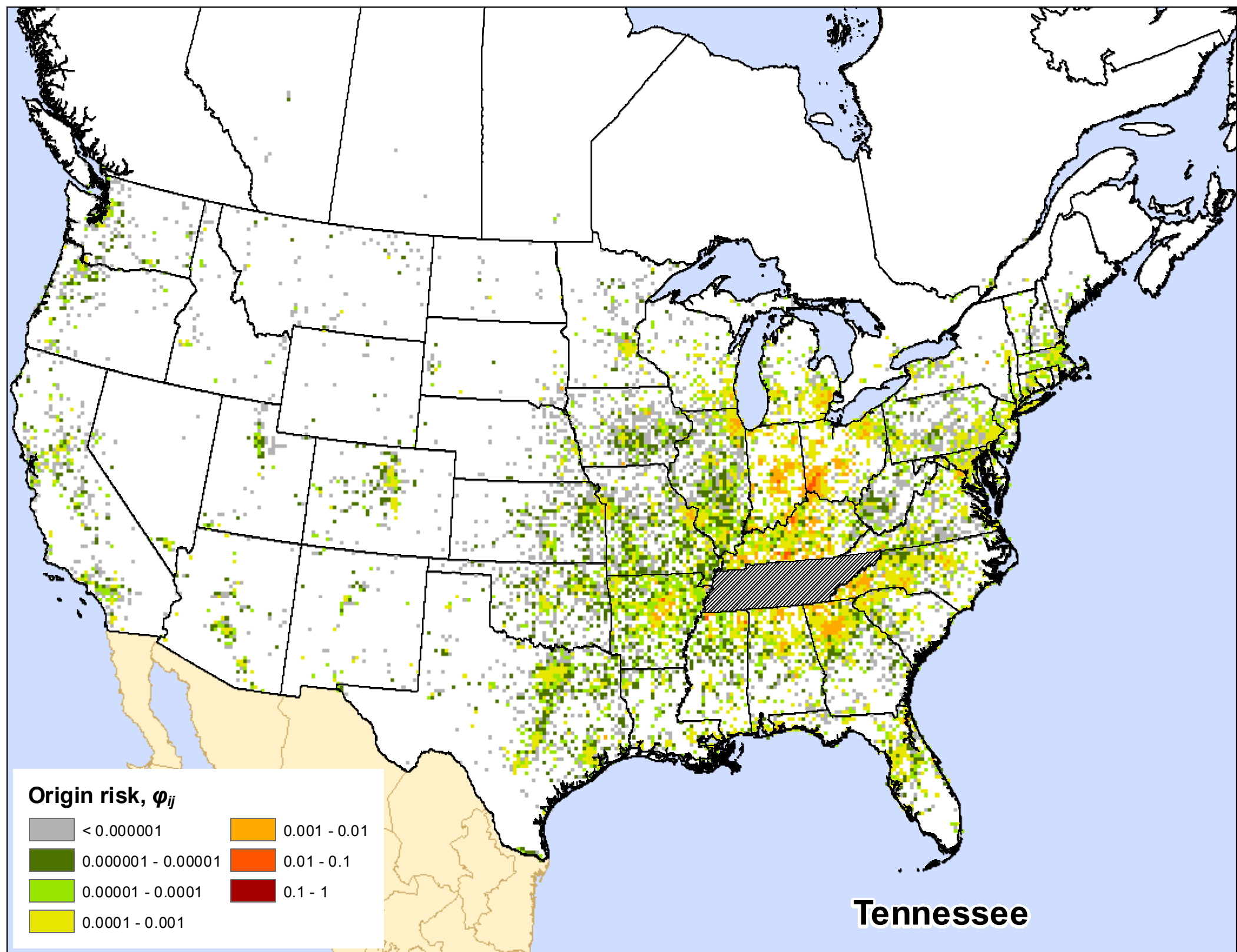

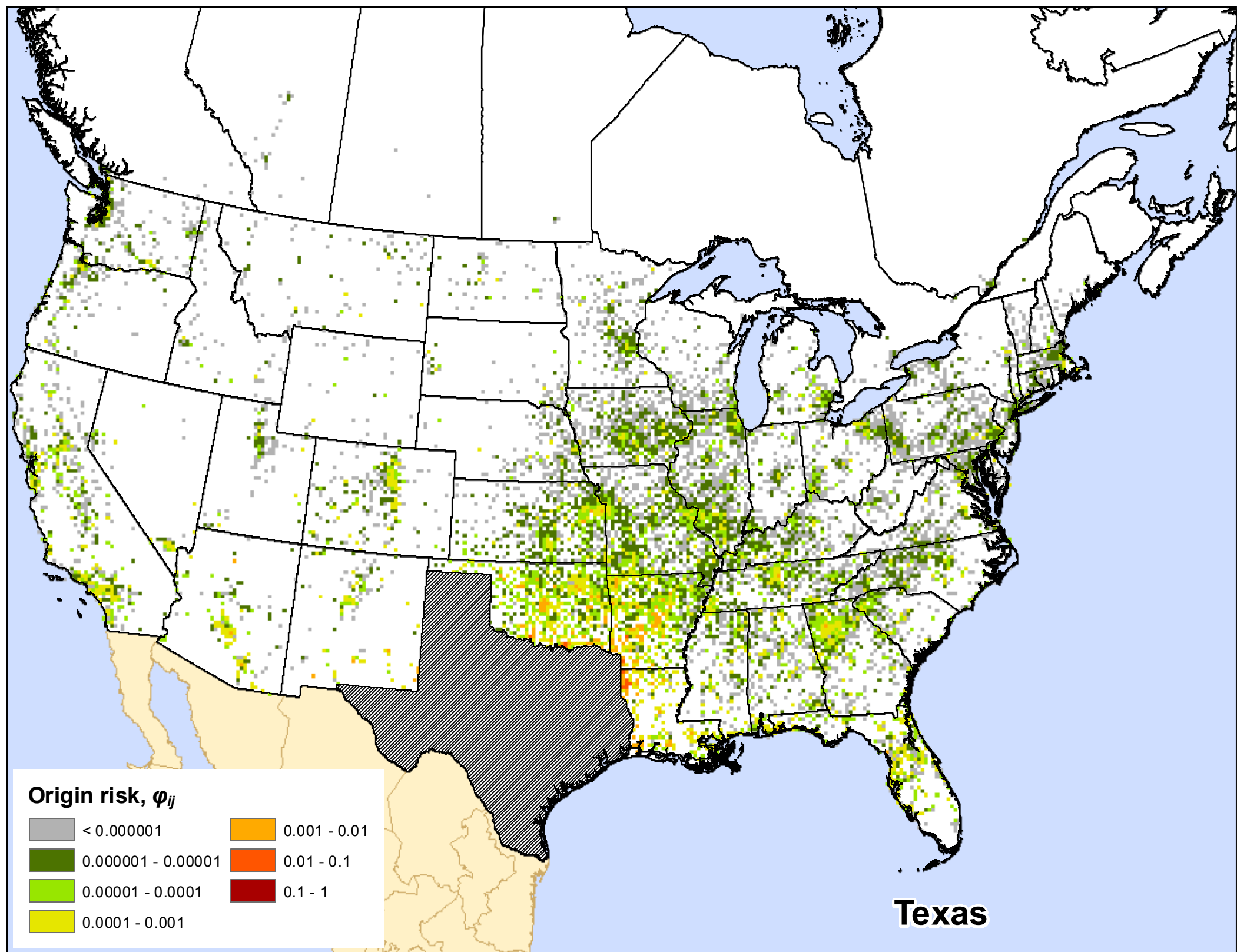

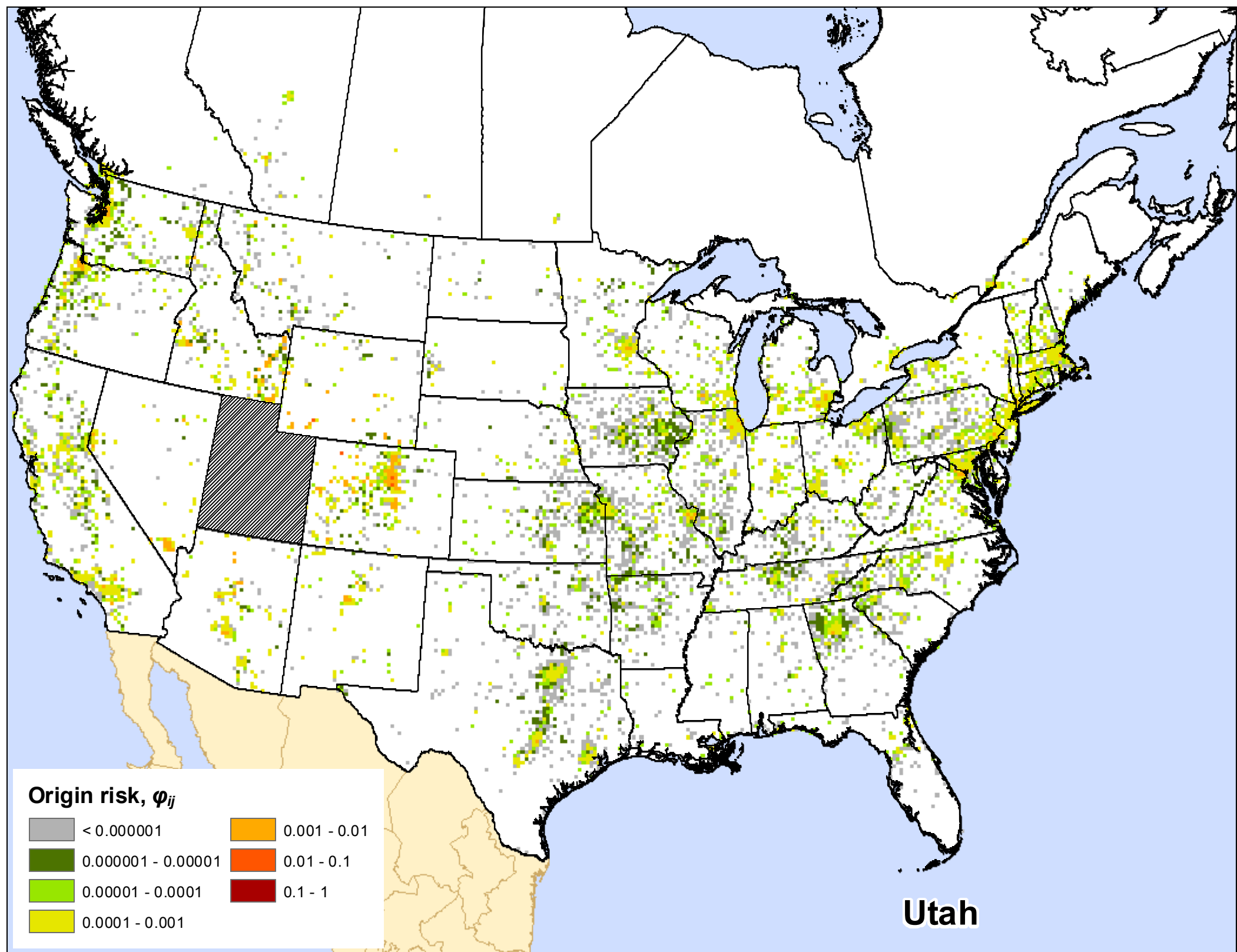

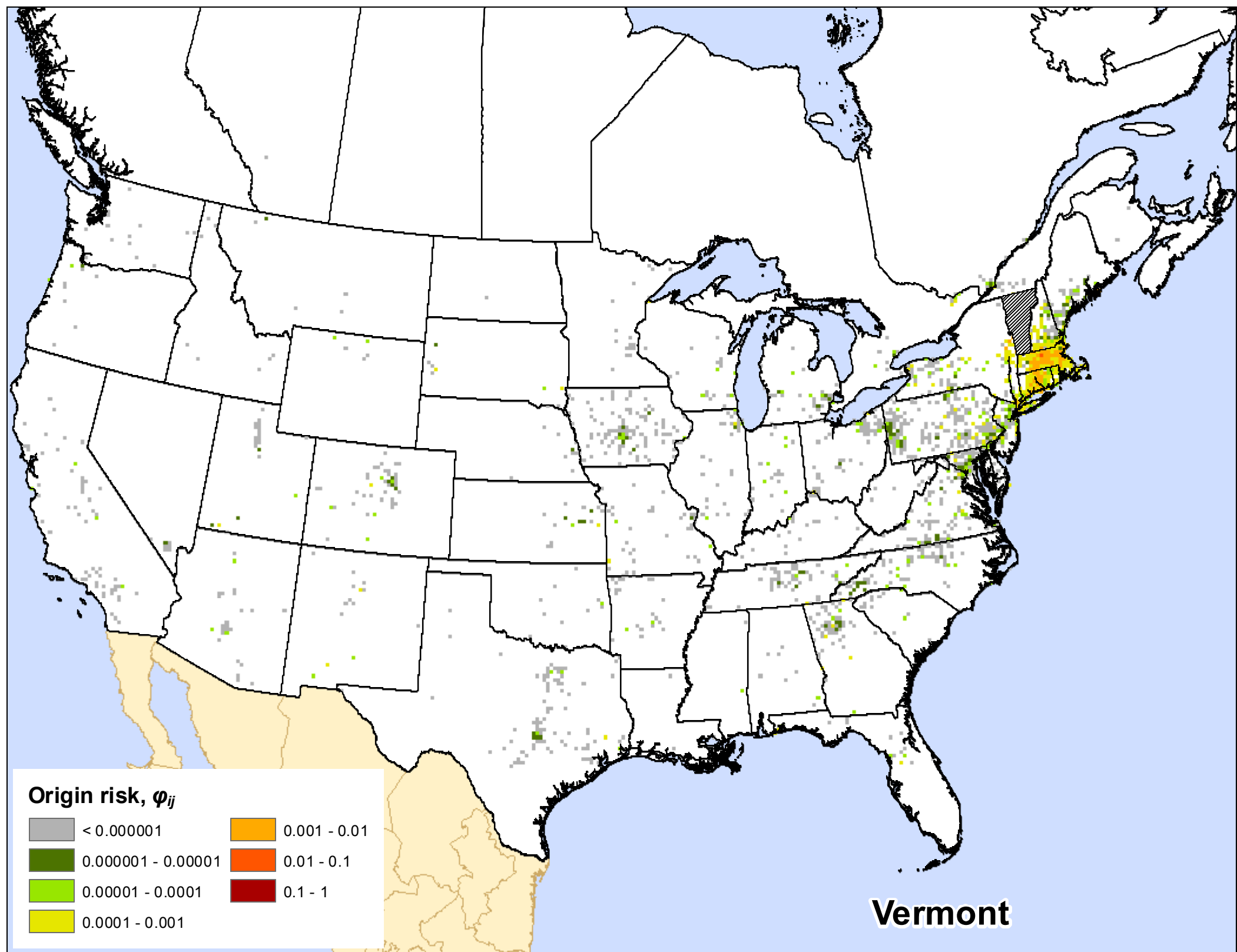

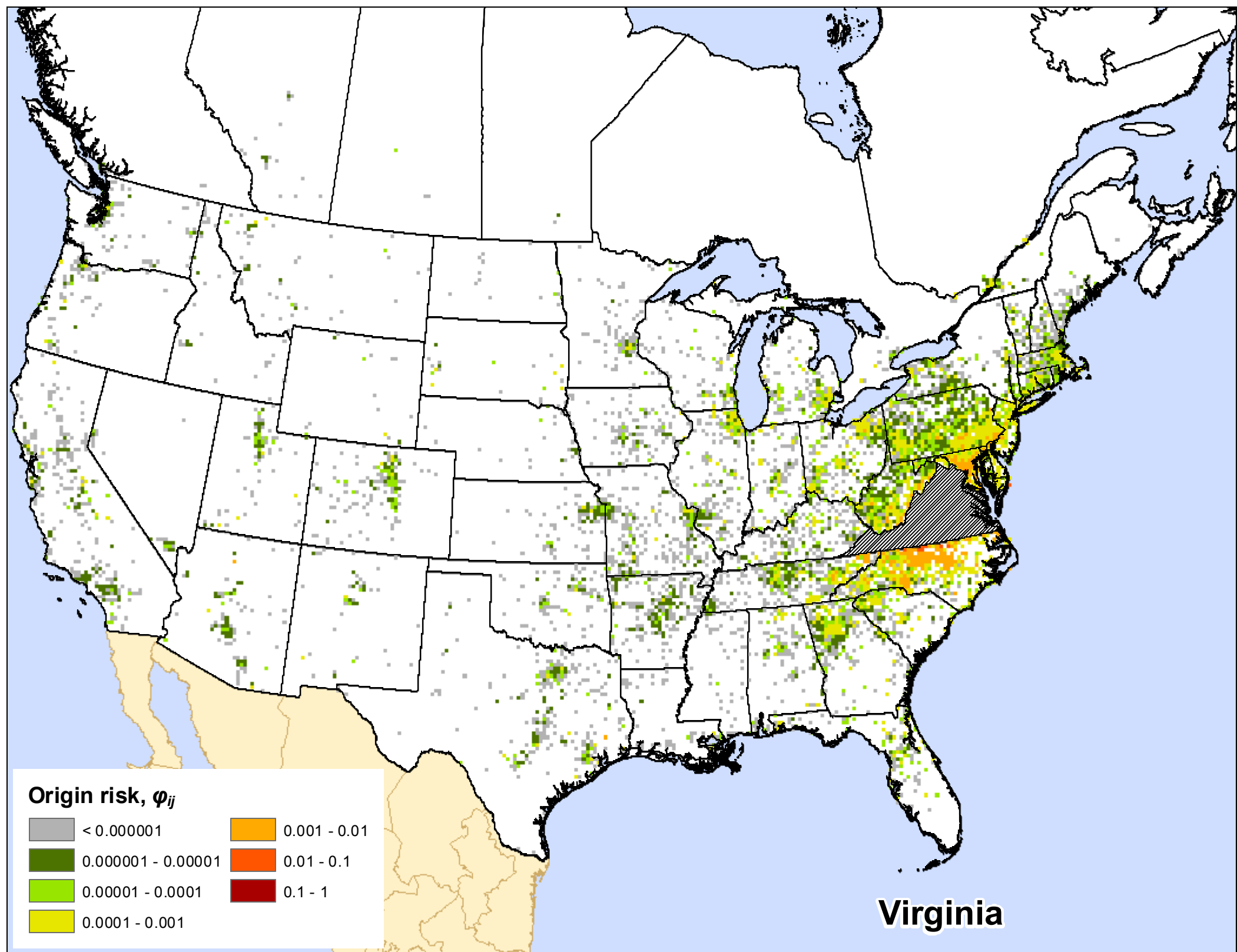

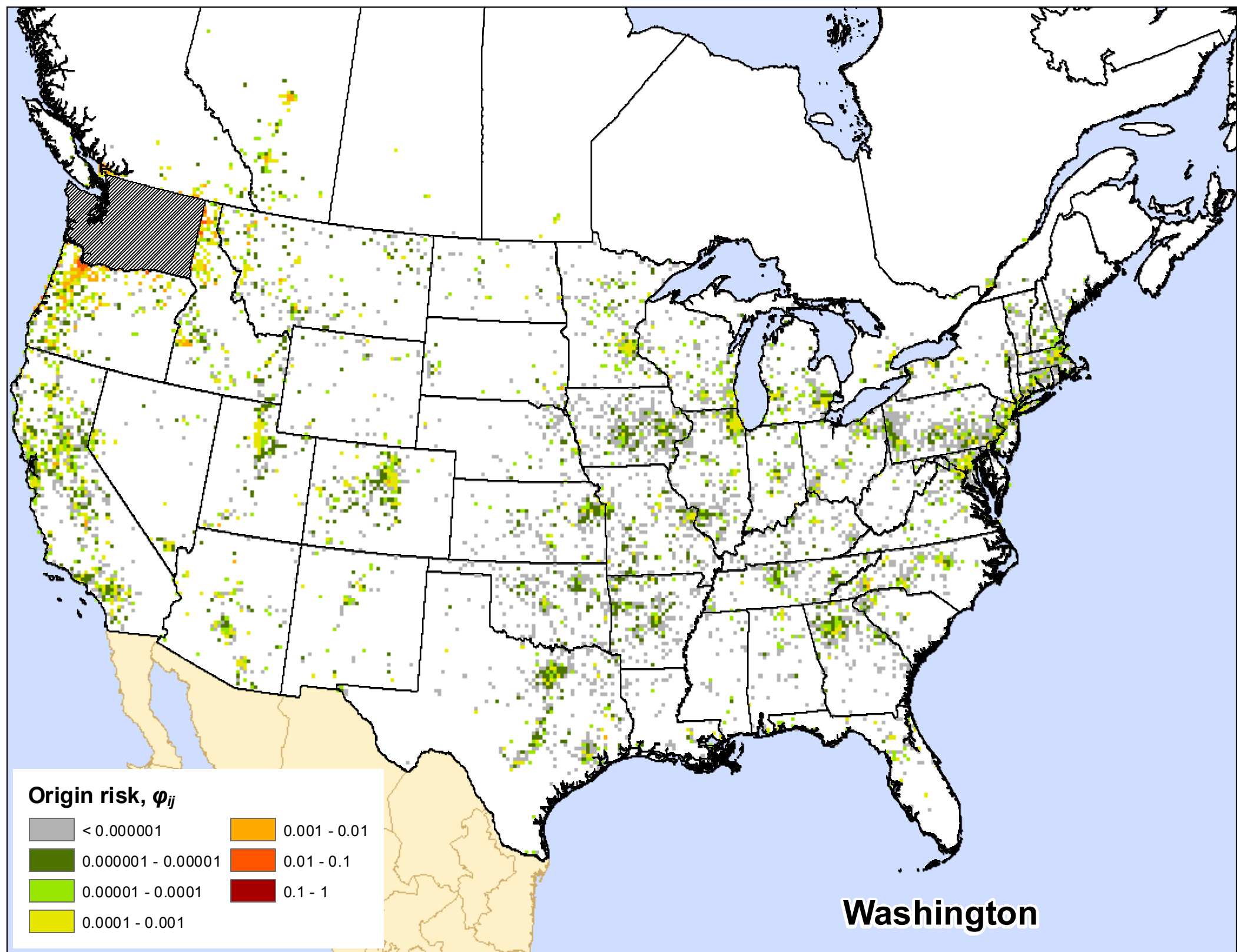

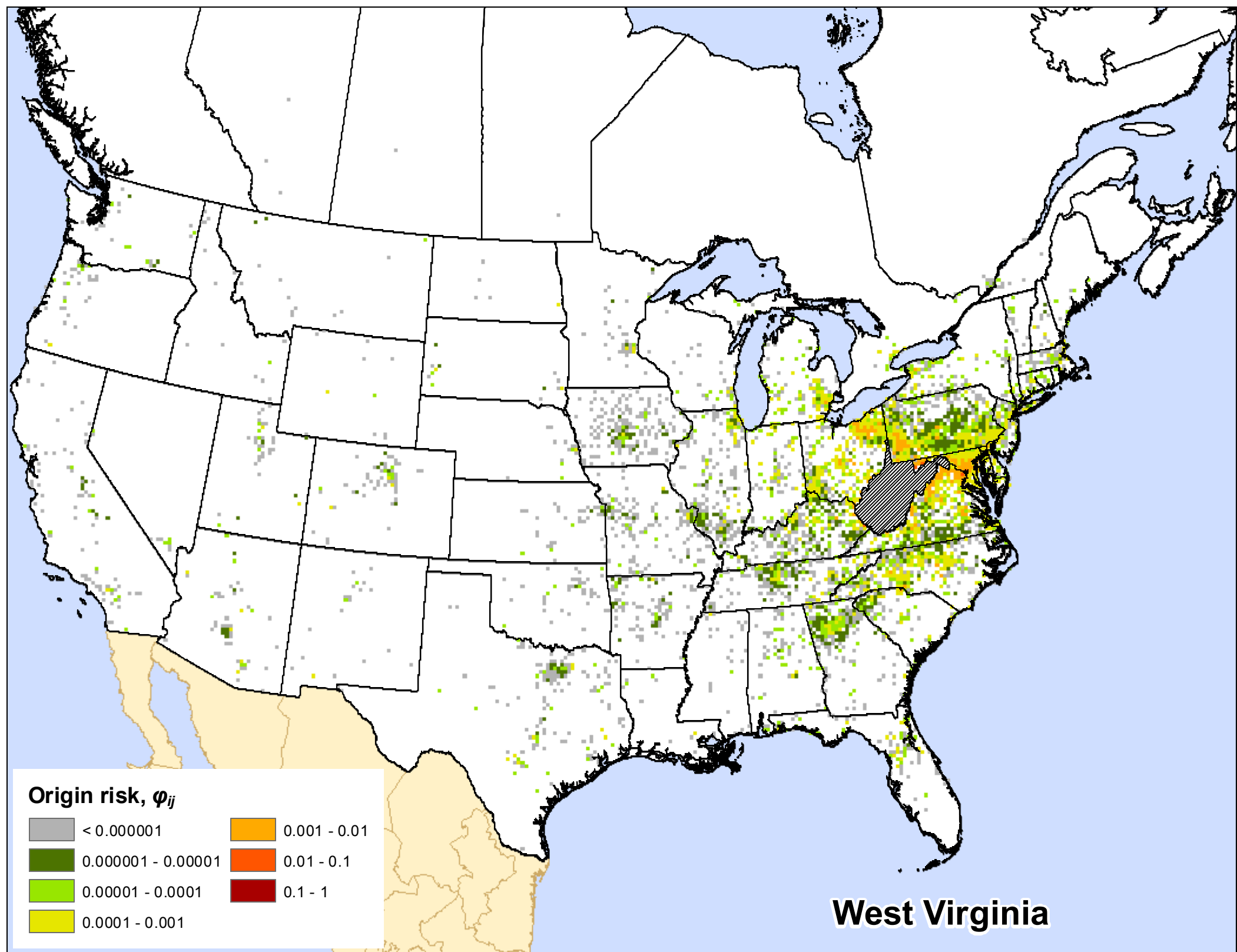

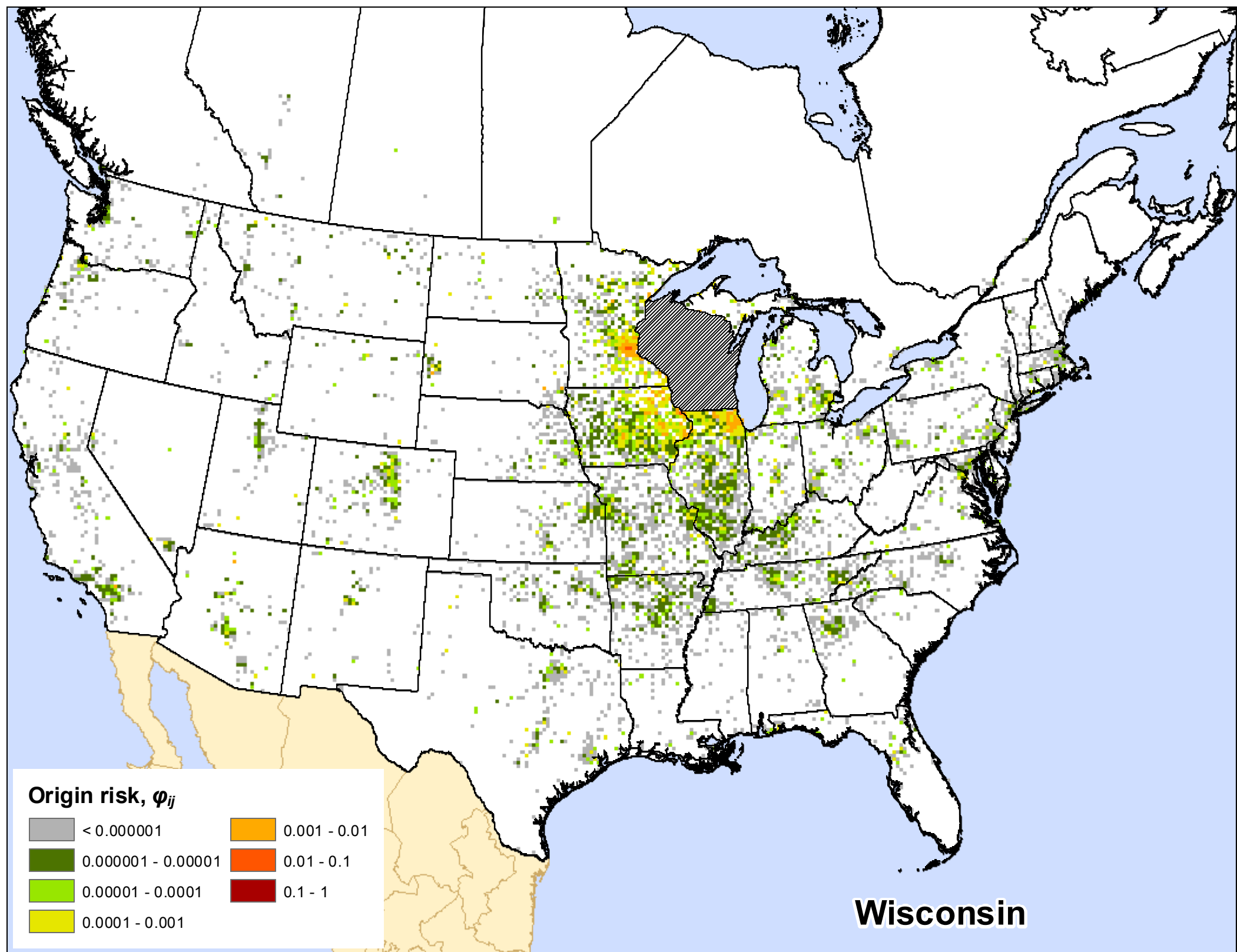

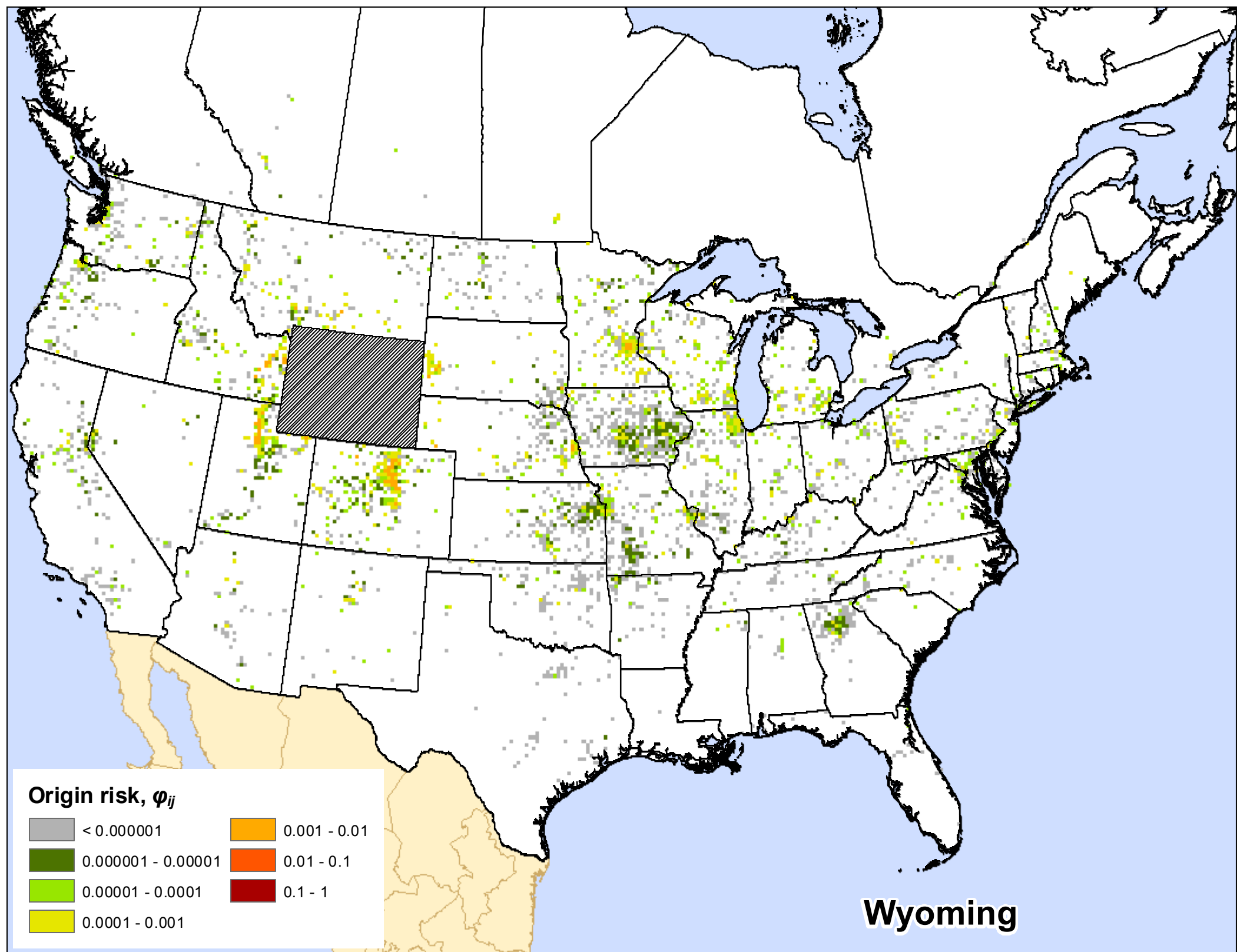

Supplement: Appendix S4 — Out-of-state origin risk maps for 24 US states: Nebraska – Wyoming. (PDF) [file pone.0102105.s004.pdf]

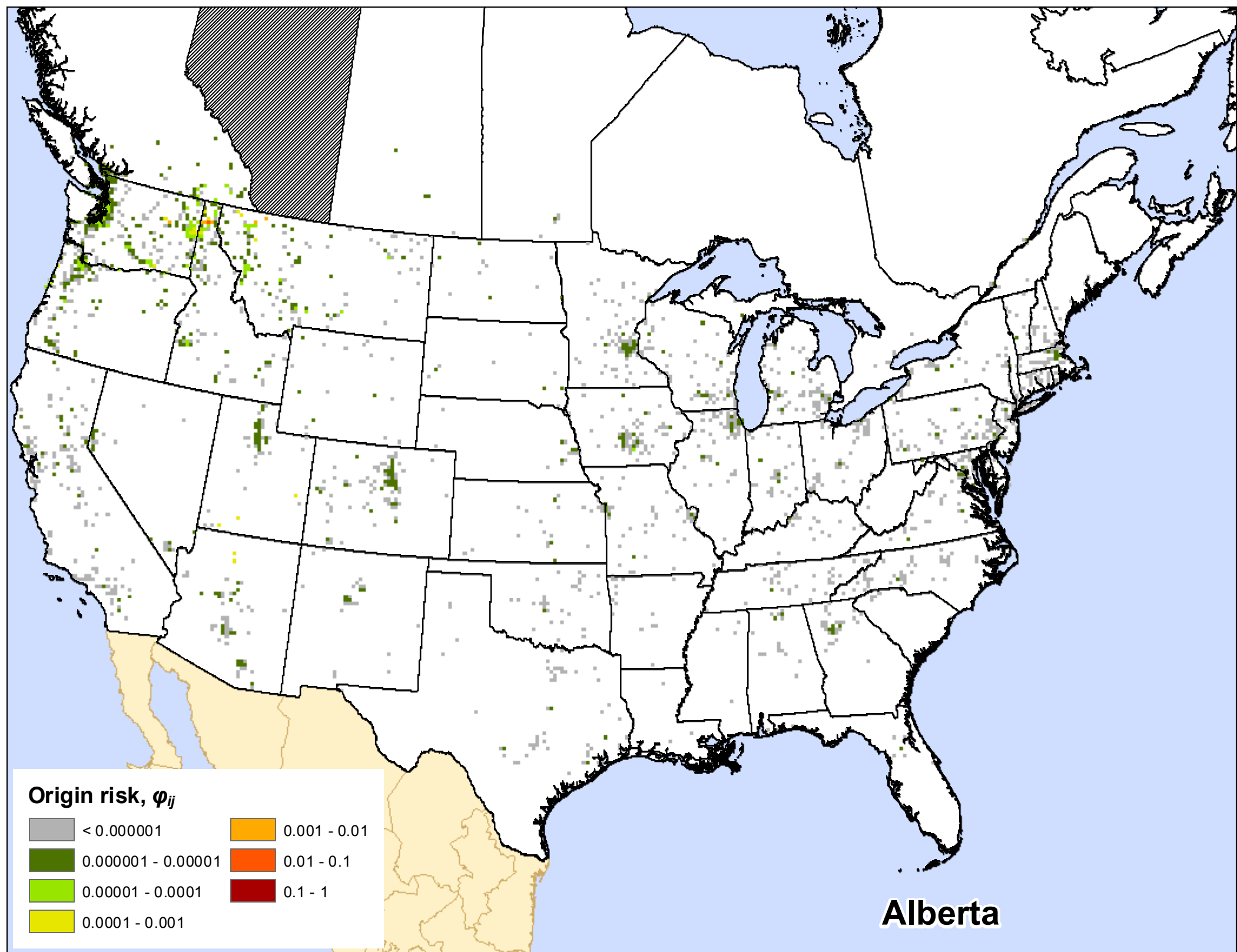

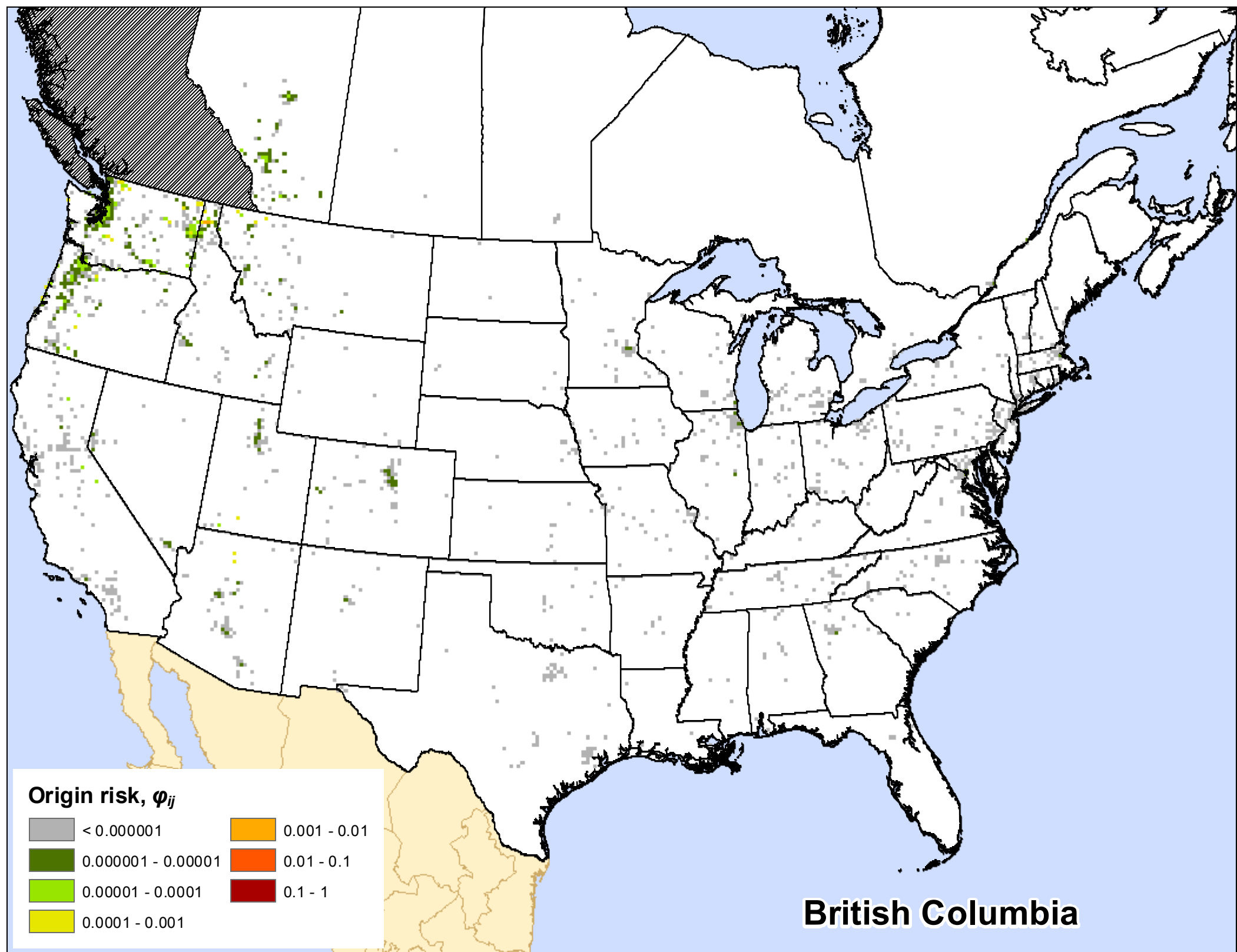

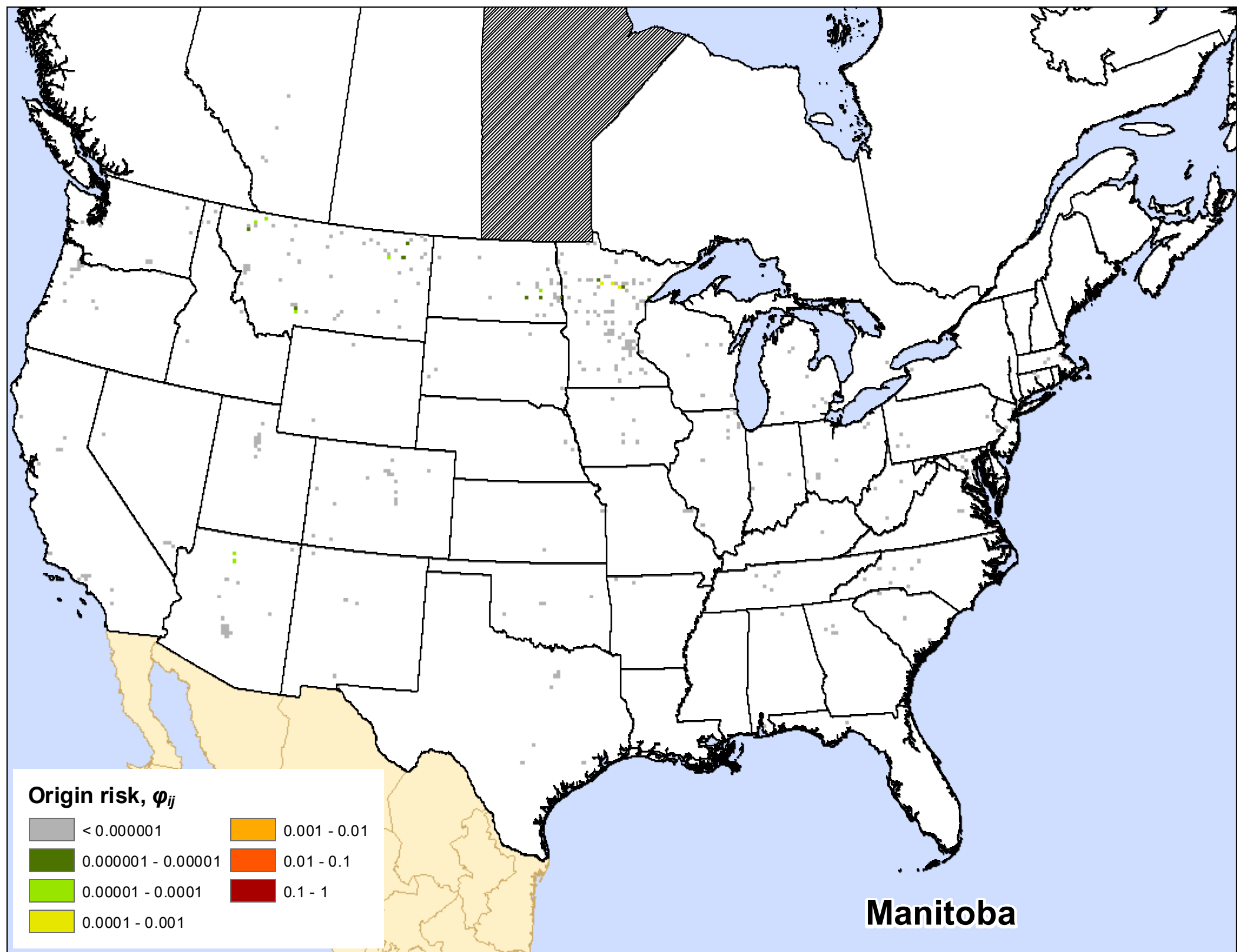

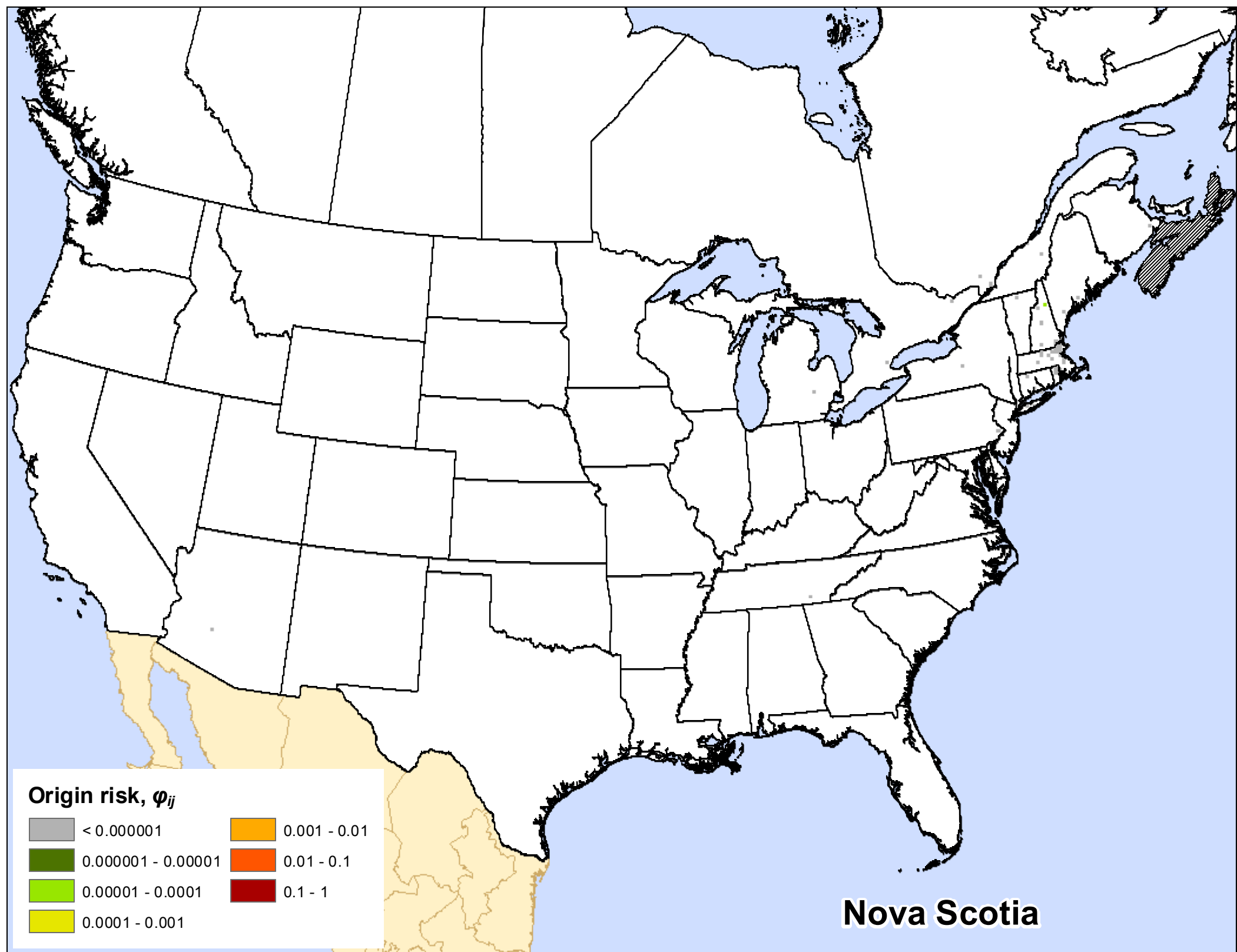

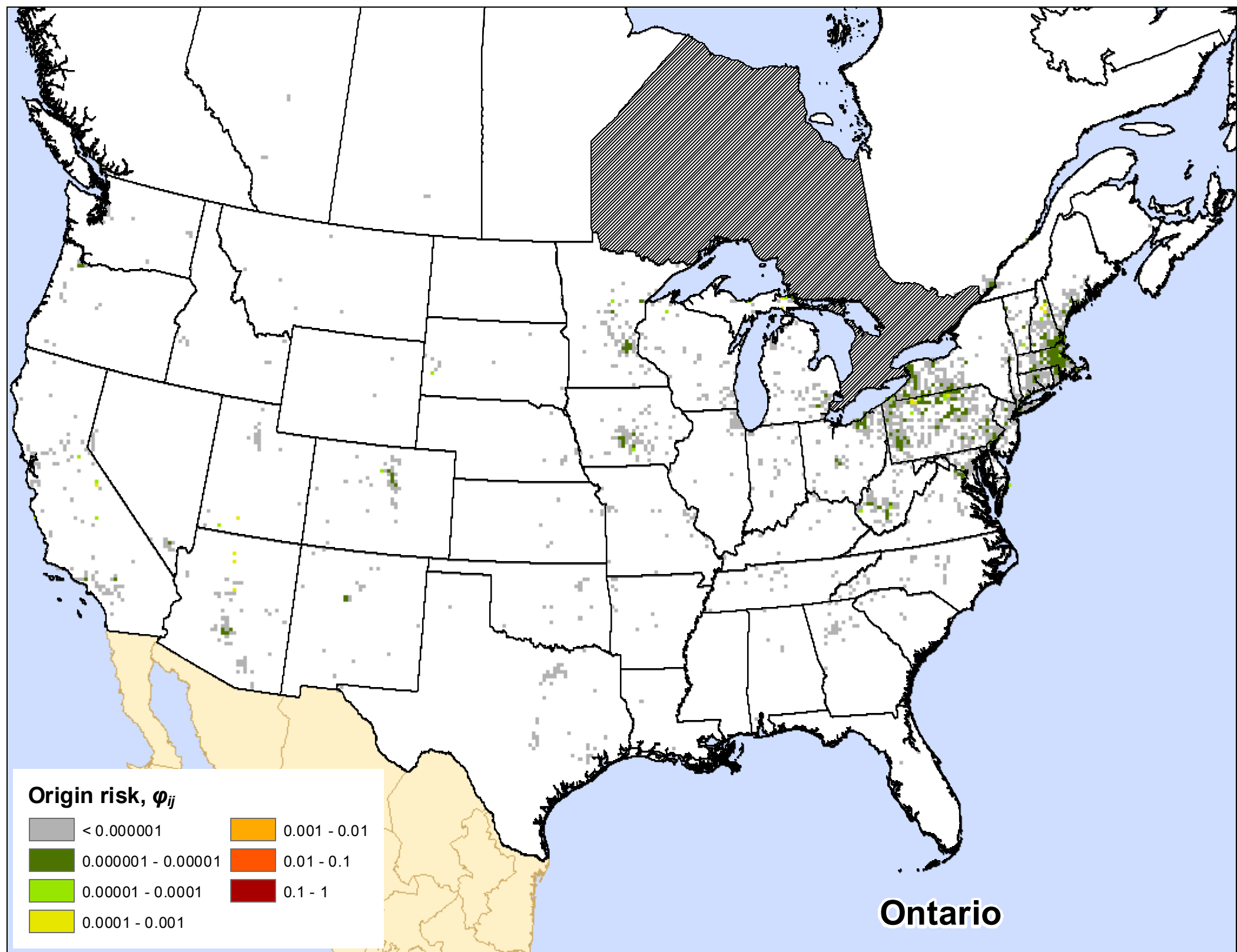

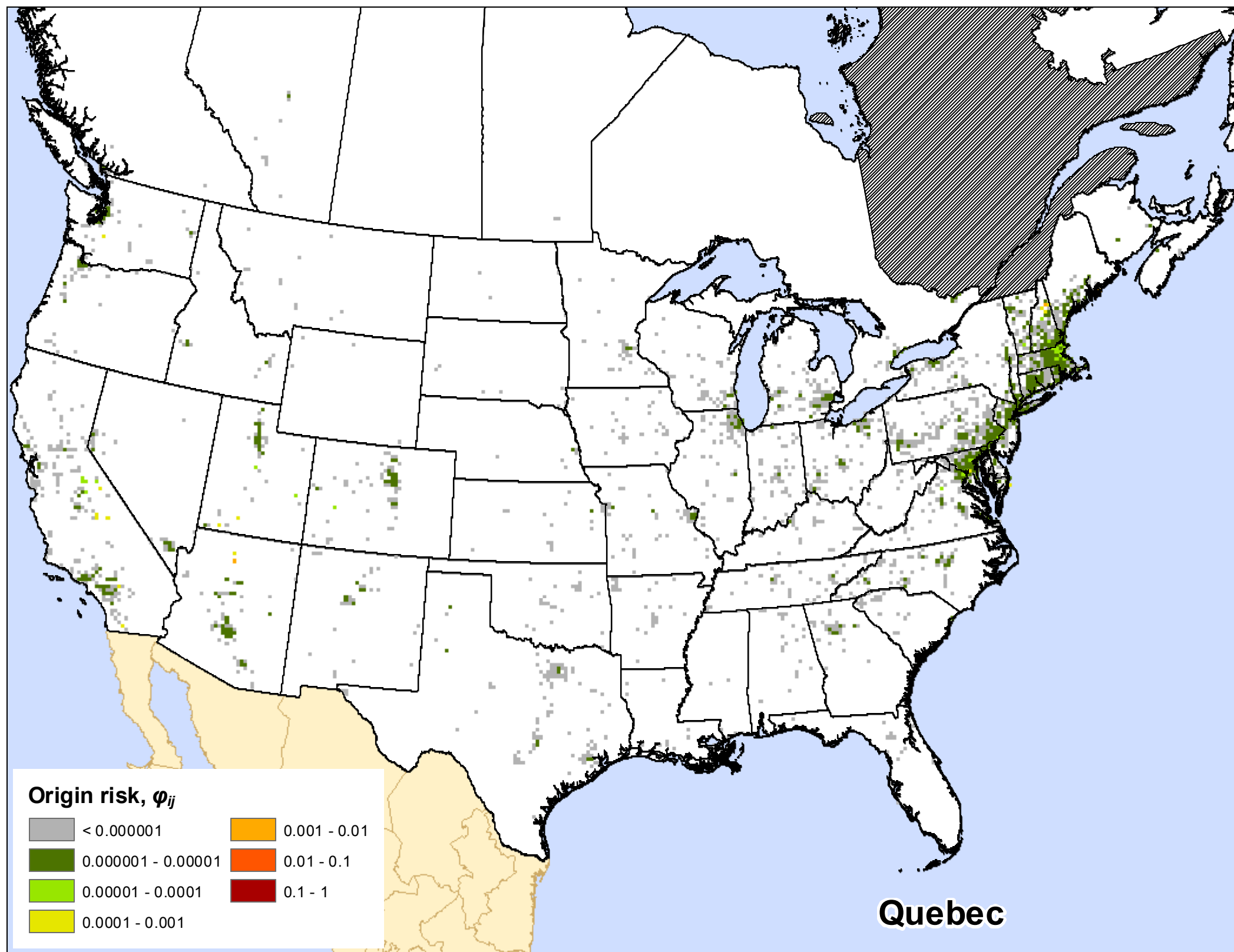

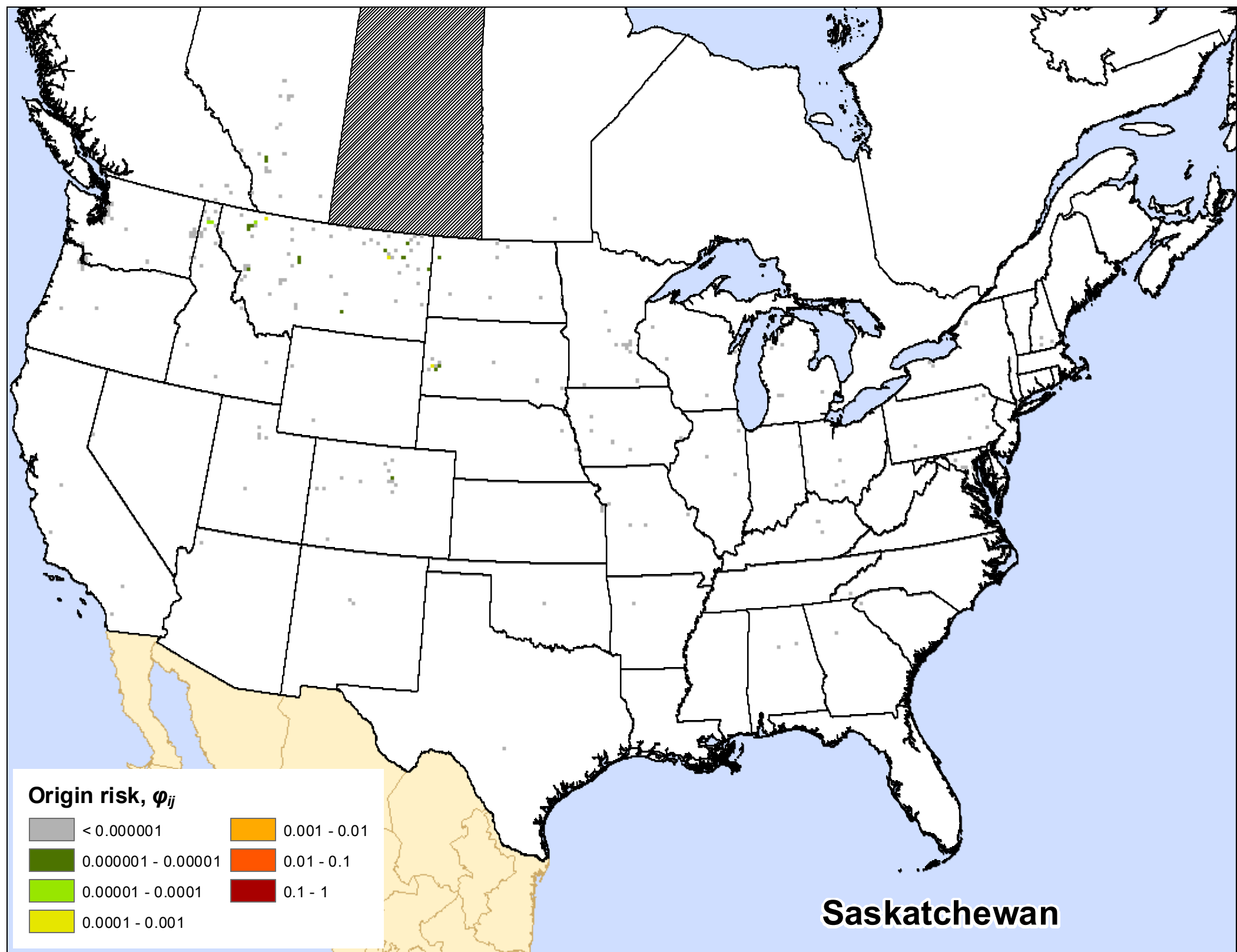

Supplement: Appendix S5 — Out-of-province origin risk maps for seven Canadian provinces: Alberta, British Columbia, Manitoba, Nova Scotia, Ontario, Quebec, and Saskatchewan. (PDF) [file pone.0102105.s005.pdf]
